# Iron-Catalyzed Acceptorless Dehydrogenative Coupling of Alcohols With Aromatic Diamines: Selective Synthesis of 1,2-Disubstituted Benzimidazoles

**DOI:** 10.3389/fchem.2020.00429

**Published:** 2020-06-19

**Authors:** Ramachandra Reddy Putta, Simin Chun, Seok Beom Lee, Dong-Chan Oh, Suckchang Hong

**Affiliations:** ^1^BK21 PLUS Project, College of Pharmacy, Seoul National University, Seoul, South Korea; ^2^Research Institute of Pharmaceutical Sciences, College of Pharmacy, Seoul National University, Seoul, South Korea; ^3^Natural Products Research Institute, College of Pharmacy, Seoul National University, Seoul, South Korea

**Keywords:** iron catalysis, dehydrogenative coupling, borrowing hydrogen, alcohol, benzimidazoles

## Abstract

Benzimidazoles are important *N*-heteroaromatic compounds with various biological activities and pharmacological applications. Herein, we present the first iron-catalyzed selective synthesis of 1,2-disubstituted benzimidazoles *via* acceptorless dehydrogenative coupling of primary alcohols with aromatic diamines. The tricarbonyl (η^4^-cyclopentadienone) iron complex catalyzed dehydrogenative cyclization, releasing water and hydrogen gas as by-products. The earth abundance and low toxicity of iron metal enable the provision of an eco-friendly and efficient catalytic method for the synthesis of benzimidazoles.

## Introduction

**Figure 1:**
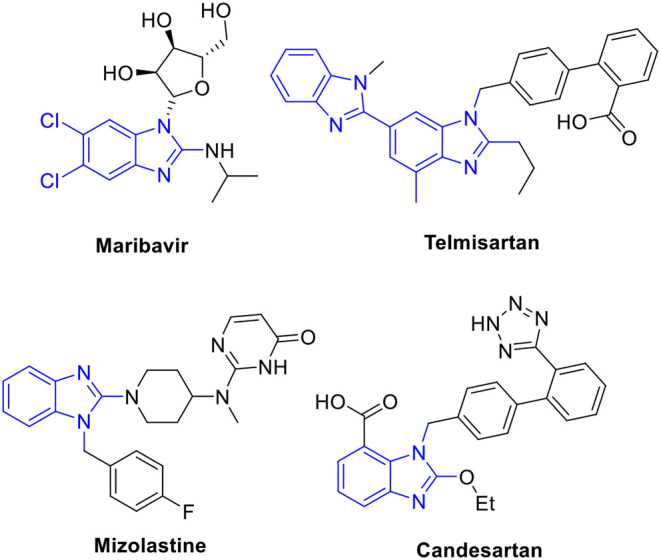
Representative drugs containing 1,2-disubstituted benzimidazole.

Benzimidazoles, which have been found in pharmaceuticals and natural products, are important *N*-heteroaromatic structural motifs because of their biological activities (Bansal and Silakari, 2012; Chandrika et al., 2016; Suk et al., 2019). Of these, the 1,2-disubstituted benzimidazole is considered a privileged scaffold in drug discovery. As shown in Figure 1, many drugs contain this moiety in their structures, for example, maribavir (antiviral), mizolastine (antihistamine), and telmisartan and candesartan (antihypertensive). Furthermore, 1,2-disubstituted benzimidazoles show various biological activities, such as anticancer (Zimmermann et al., 2013, 2014) antibacterial (Bandyopadhyay et al., 2011; Göker et al., 2016), antiallergic (Nakano et al., 2000), and anti-HIV (Morningstar et al., 2007) traits along with cannabinoid-1 (CB1) and cannabinoid-2 (CB2) receptors (Watson et al., 2011; Nanda et al., 2014). Based on their attractive biological profiles, the synthesis of 1,2-disubstituted benzimidazoles has gained the interest of synthetic chemists.

**Scheme 1:**
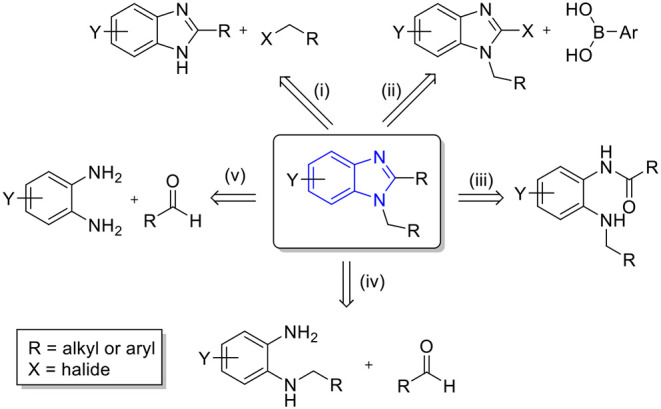
Diverse synthetic strategies for 1,2-disubstituted benzimidazoles.

Diverse synthetic approaches have been reported for the synthesis of 1,2-disubstituted benzimidazoles (Scheme 1). The first is the respective substitution on the *C*-1 or *N*-2 position of the preformed benzimidazoles, (i) *N*-alkylation of 2-substituted benzimidazoles (Ramla et al., 2007; Martin et al., 2015) and (ii) Suzuki coupling of aryl boronic acids with 1-halo-2-alkylbenzimidazoles (Wang and Smith, 2003; Martin et al., 2015). Another approach is the classic cyclocondensation of (iii) *N*-alkyl-*N*-acyl-1,2-diaminobenzene (Smith and Krchnák, 1999; Takeuchi et al., 2000) or (iv) *N*-alkyl-1,2-diaminobenzene with aldehyde (Smith and Krchnák, 1999; Özden et al., 2005). In addition, a large number of (v) direct one-pot cyclocondensations of 1,2-diaminobenzene with aldehydes have been reported (Chebolu et al., 2012; Girish et al., 2015; Sharma et al., 2015). This appears to be a straightforward approach; however, selectivity control between 2-substituted and 1,2-disubstituted benzimidazoles is often problematic.

**Figure 2:**
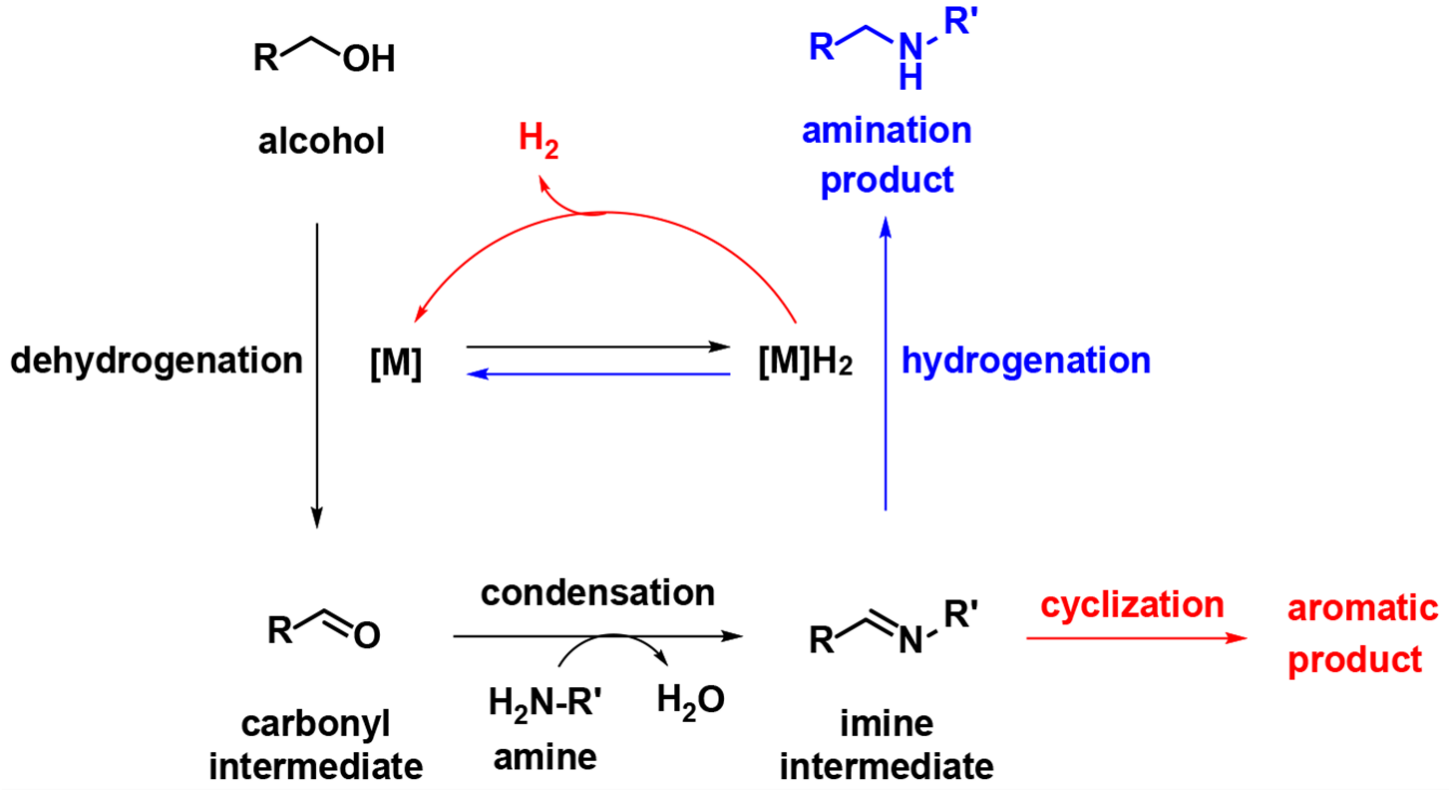
The general mechanism of the BH and ADC in the coupling of alcohol with amine.

**Scheme 2:**
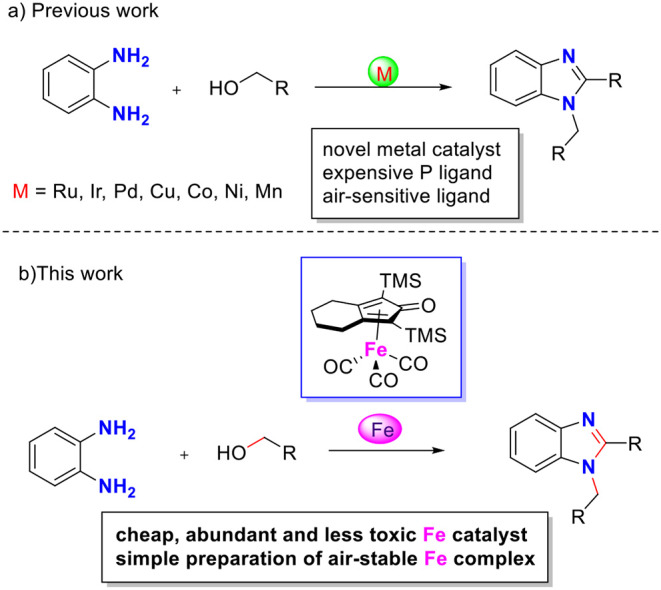
Transition metal catalyzed 1,2-disubstituted benzimidazole synthesis *via* dehydrogenative coupling of alcohol.

During the past decade, the borrowing hydrogen (BH) strategy has become a powerful tool for the benign and sustainable construction of C–N bonds using abundant alcohols as coupling reagents (Mutti et al., 2015; Yang et al., 2015). C–N bond couplings through BH usually proceed in the following sequence (Figure 2, blue): dehydrogenation of an alcohol to the corresponding carbonyl compound, followed by condensation and reduction of imine using the borrowed H_2_ from the alcohol. If the imine participates in the aromatic system, the last hydrogenation step is suppressed, and the hydrogen gas is liberated (Figure 2, red), so it is called acceptorless dehydrogenative coupling (ADC). ADCs are highly efficient and environmentally benign methods to construct *N*-heteroaromatic structures since water and hydrogen gas are the only valuable by-products (Gunanathan and Milstein, 2013; Michlik and Kempe, 2013; Nandakumar et al., 2015). In recent years, considerable progress has been directed toward the synthesis of benzimidazole involving dehydrogenative coupling (Scheme 2); however, most of these methods use precious noble metals, such as Ru- (Blacker et al., 2009; Li et al., 2018), Ir- (Hille et al., 2014; Sharma et al., 2017), and Pd-based catalysts (Mori et al., 2019). The replacement of noble-metal catalysts by inexpensive and environmentally friendly earth-abundant base metals is an important task for organic chemists. Among the base metals, Cu- (Xu et al., 2017, 2018), Co- (Daw et al., 2017), Ni- (Bera et al., 2019), and Mn-based catalysts (Das et al., 2018; Zhang et al., 2019) have been well-applied in the condensation of alcohols with 1,2-diaminobenzene to benzimidazoles. However, many of these metal complexes utilize quite expensive or labile ligands to achieve higher product yields, which is a major concern in comparison to the advantages of base metals. Iron is the second most earth-abundant and highly desirable metal catalyst in the synthesis of pharmaceuticals due to its low toxicity (Bauer and Knölker, 2015; Fürstner, 2016). The tricarbonyl (η^4^-cyclopentadienone) iron complexes were initially described by Knölker (Knölker et al., 1999), and they have a core bifunctional structure to mediate the BH process consisting of both a proton-donor site (ligand) and a hydride-donor site (metal center) (Ikariya and Blacker, 2007). Since it was first developed, Knölker's complex has been widely applied in C–N or C–C bond formation through a BH strategy (Yan et al., 2014; Brown et al., 2017; Reed-Berendt et al., 2019). Based on the previous results, we envisioned the possibility of iron-catalyzed direct benzimidazole formation starting from 1,2-diaminobenzene and alcohol *via* the ADC strategy. To the best of our knowledge, the synthesis of benzimidazoles directly from 1,2-diaminobenzene and alcohol catalyzed by iron has not been reported. Herein, we describe a selective method to synthesize 1,2-disubstituted benzimidazoles using Knölker-type iron complexes as a catalyst.

## Materials and Methods

All catalytic reactions were carried out under nitrogen atmosphere using a Schlenk flask. Fe complexes **cat. I**–**V** (Moulin et al., 2013) and **cat. VI** (Dambatta et al., 2019) were prepared according to the literature. All commercially available reagents and solvents (purchased from Sigma-Aldrich, TCI, Alfa-Aesar and Acros) were used without further purification unless otherwise noted. Reactions were monitored by thin-layer chromatography on silica gel 60 F254 plate using UV illumination at 254 nm. Column chromatography was performed on silica gel (230–400 mesh), using a mixture of hexane and ethyl acetate as eluents. Nuclear magnetic resonance (^1^H-NMR and ^13^C-NMR) spectra were measured on JEOL JNM-ECZ400s [400 MHz (^1^H), 100 MHz (^13^C)], using CDCl_3_ as solvent. It was reported in ppm relative to CDCl_3_ (δ 7.26) for ^1^H-NMR and relative to the central CDCl_3_ (δ 77.16) for ^13^C-NMR. Coupling constants (*J*) in ^1^H-NMR and ^13^C-NMR are in hertz. All high-resolution mass spectra (HR-MS) were acquired under fast atom bombardment (FAB) condition on a JMS-700 MStation mass spectrometer. Melting points were measured on a Büchi B-540 melting point apparatus and were not corrected. X-ray diffraction studies were carried out in a Super Nova, Dual, Mo at home/near, Atlas S2 diffractometer.

### General Procedure for the Synthesis of 1-Benzyl-2-aryl-1*H*-benzo[*d*]imidazoles (3)

In a 15-ml Schlenk flask, a mixture of 1,2-diaminobenzene (**1a**, 54.05 mg, 0.5 mmol), alcohol (**2**, 1.5 mmol), ^*t*^BuOK (84.16 mg, 0.75 mmol), **cat. I** (8.36 mg, 0.02 mmol), and TMAO (3.0 mg, 0.04 mmol) was stirred at 150°C in xylene (2 ml) for 24 h under a nitrogen atmosphere. Then, the reaction mixture was cooled to room temperature and diluted with dichloromethane. After removing the solvent, the resulting residue was further purified by column chromatography on silica gel using 10–30% ethyl acetate in hexane as an eluent to obtain the desired benzimidazoles.

#### 1-Benzyl-2-phenyl-1*H*-benzo[*d*]imidazole (3a)

Following the general procedure with **1a** and **2a**, **3a** was obtained as white solid (121 mg, 85% yield). m.p. 128–130°C. ^1^H-NMR (400 MHz, CDCl_3_) δ 7.88 (d, *J* = 7.8 Hz, 1H), 7.69 (dd, *J* = 7.5, 1.6 Hz, 2H), 7.46 (dd, *J* = 13.0, 5.7 Hz, 3H), 7.29–7.33 (m, 4H), 7.23 (q, *J* = 7.5 Hz, 2H), 7.11 (d, *J* = 6.9 Hz, 2H), 5.46 (s, 2H). ^13^C-NMR (100 MHz, CDCl_3_) δ 154.24, 143.27, 136.46, 136.14, 130.15, 129.99, 129.31, 129.12, 128.83, 127.84, 126.02, 123.10, 122.74, 120.06, 110.61, 48.43. HRMS (FAB^+^) *m*/*z* calcd for C_20_H_17_N_2_ [M+H]^+^: 285.1392, found: 285.1382.

## Results and Discussion

In a preliminary study, we explored the feasibility of benzimidazole formation between 1,2-diaminobenzene **1a** and benzyl alcohol **2a** using standard Knölker complex **cat. I** (Table 1). The reaction was carried out in toluene, and trimethylamine *N*-oxide (TMAO) was used to activate **cat. I** and liberate a vacant site *in situ*. In the first trial, no benzimidazole products were formed in the absence of a base (entry 1). Based on previous reports (Xu et al., 2017; Das et al., 2018), we expected that a stoichiometric amount of base is required for benzimidazole formation. Various kinds of bases were screened in the reaction system, and ^*t*^BuOK was found to be a more effective base than KOH and K_2_CO_3_ for the formation of 1,2-disubstituted benzimidazoles **3a** (entries 2–4). Surprisingly, we could not detect any 2-*mono*-substituted benzimidazole product in the reaction. Higher conversion was achieved when the reaction temperature was increased from 130 to 150°C (entries 4 and 5). Next, we examined the efficacy of different solvents and neat conditions, and the best yield of **3a** (85%) was obtained in xylene (entries 5–8). The control experiment was also performed, and it was revealed that no desired product was obtained in the absence of a catalyst, demonstrating a crucial role of iron complex in the dehydrogenative coupling (entry 9). Additionally, we tried to reduce the amount of alcohol **2a** and base in the reaction; however, slightly lower yields were observed (entries 10 and 11). To investigate a feasibility for the selective synthesis of 2-*mono*-substituted benzimidazole, 1.0 equivalent of **2a** was reacted with **1a**. Unfortunately, 1,2-disubstituted and 2-substituted benzimidazoles were obtained in 17 and 6% yields, respectively (entry 12).

**Table 1:** Optimization of the reaction conditions^a^.

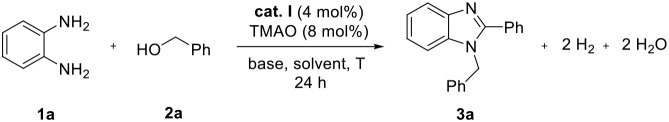					
**Entry**	**Alcohol (eq)**	**base (eq)**	**Solvent**	**T (°C)**	**Yield**^**B**^ **(%)**
1	3	–	Toluene	130	–
2	3	K_2_CO_3_ (1.5)	Toluene	130	Trace
3	3	KOH (1.5)	Toluene	130	21
4	3	*^*t*^*BuOK (1.5)	Toluene	130	42
5	3	*^*t*^*BuOK (1.5)	Toluene	150	61
6	3	*^*t*^*BuOK (1.5)	Dioxane	150	31
**7**	**3**	*^***t***^***BuOK (1.5)**	**Xylene**	**150**	**85**
8	3	*^*t*^*BuOK (1.5)	Neat	150	53
9^c^	3	*^*t*^*BuOK (1.5)	Xylene	150	–
10	3	*^*t*^*BuOK (1.2)	Xylene	150	81
11	2.5	*^*t*^*BuOK (1.5)	Xylene	150	80
12^d^	1	*^*t*^*BuOK (1.5)	Xylene	150	17

a *Reaction conditions: **1a** (0.5 mmol), **2a** (0.5–1.5 mmol), base (0.6–0.75 mmol), **cat. I** (0.02 mmol), TMAO (0.04 mmol), and solvent (2 ml) in a Schlenk flask under N_2_, 24 h*.

b *Isolated yield*.

c *No catalyst loading*.

d *2-Mono-substituted benzimidazole product was obtained with **3a** in 6% yield*.

As we were optimizing the reaction conditions, the effect of the amount of catalysts was also investigated (Table 2). Decreasing the loading of catalyst from 4 to 3 mol% resulted in 80% yield of desired product **3a**, and a small amount of diamine substrate **1a** remained. Interestingly, when we increased the catalyst loading to 5 mol%, a significantly decreased yield of **3a** and increased formation of *N, N*-dibenzylbenzene-1,2-diamine **4** were observed. We supposed that a large amount of catalyst accelerated imine reduction competitively with the annulation process. Various Knölker-type complexes were also explored to estimate their activity in the reaction, and the results are shown in Table 2. The **cat. VI** gave desired product **3a** in good yield (80%), similar to that of **cat. I**. However, the **cat. II** and **IV** showed moderate efficiency and **cat. III** and **V** resulted in low efficiency. Based on the above results, we choose the optimal dehydrogenative coupling conditions as diamine **1** (1.0 equiv.), alcohol **2** (3.0 equiv.), **cat. I** (4 mol%), TMAO (8 mol%), and ^*t*^BuOK (1.5 equiv.) in xylene (2 ml) at 150°C under N_2_ for 24 h.

**Table 2:** Catalyst screening^a^^,^^b^.

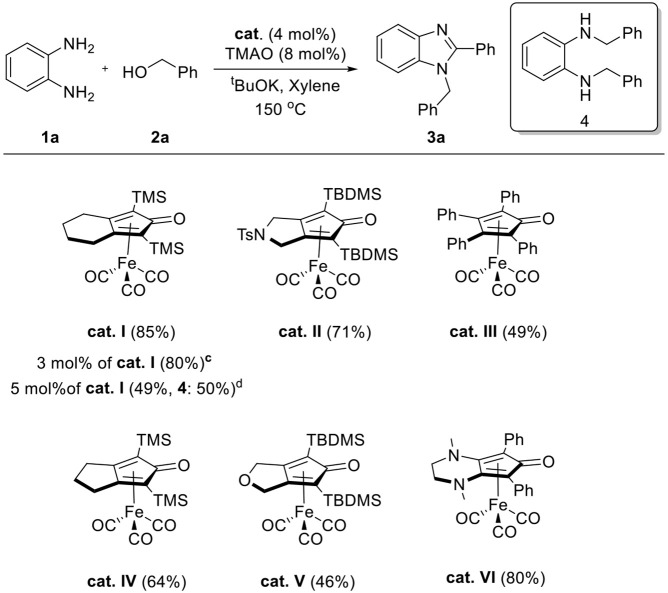

a *Reaction conditions: **1a** (0.5 mmol), **2a** (1.5 mmol), ^t^BuOK (0.75 mmol), **cat. I** (0.02 mmol), TMAO (0.04 mmol), and xylene (2 ml) in Schlenk flasks under N_2_, 24 h, 150°C*.

b *Isolated yield in parentheses*.

*^c^**Cat. I** (0.015 mmol) and TMAO (0.03 mmol)*.

*^d^**Cat. I** (0.025 mmol) and TMAO (0.05 mmol)*.

We applied the optimized conditions on a variety of diamines **1** and alcohols **2** to explore the reaction scope. First, a wide range of alcohol **2** was employed for annulation with **1a** (Table 3). Benzyl alcohols containing electron-donating groups in the phenyl ring showed good yields (**3b**–**f**, 75–83%). The steric effect slightly influenced the formation of the desired product, depending on the position of the substituent. Substrates with substituents at the *ortho* position showed slightly lower yields than those of *meta-* and *para*-substituted analogs (yield sequence order: *para* > *meta* > *ortho*). 4-Chlorobenzyl alcohol afforded excellent yield for the corresponding product (**3g**, 92%), whereas low yield was obtained in the case of bromo- and iodo-substituted analogs with loss of one halogen atom (**3h** and **3i**, 52–59%). This partial dehalogenation might be involved in the hydrogenative activity of the hydrogenated iron complex, which could be formed *in situ*. The molecular structure of **3i** was confirmed by X-ray crystal structure as shown in Table 3. XRD data showed that the *N*-substituted benzyl group has iodine and *C*-2-substituted phenyl ring loose iodine. A series of alcohols containing heterocycles, such as furan, thiophene, and pyridine, were well-applied and afforded the desired products in good yields (**3j**–**l**, 72–82%). In the case of 1,3-benzodioxole-5-methanol and 4-trifluoromethyl benzyl alcohol, the desired products were obtained in moderate yield even if a longer reaction time is needed for full conversion (**3m** and **3n**, 67–68%). Additionally, 1-naphthalene methanol was also applied in the reaction system and gave the corresponding product in high yield (**3o**, 85%). For further expansion of the alcohol scope, aliphatic alcohols such as 1-hexanol and 3-phenyl propanol were also investigated. Aliphatic alcohols could participate in dehydrogenative coupling; however, desired products were obtained in low yields (**3p**–**q**, 38–40%). After the screening of alcohols, the scope of diamine **1** was also investigated (Table 4). Under the same conditions, the reaction of 4,5-dimethyl-1,2-diaminobenzene with **2a** proceeded smoothly and afforded the product inwas also investigated good yield (**5a**, 81%). On the other hand, 3,4-diaminotoluene and 4-chloro-1,2-diaminobenzene gave a mixture of 1-benzyl-2-phenyl-benzimidazole products (**5b** and **5c**). To explore the possibility of imidazole formation, we employed 1,2-diphenyl-1,2-ethylenediamine as a substrate. Unfortunately, the corresponding imidazole product was obtained in very low yield (**5d**, 20%).

**Table 3:** Scope of alcohols^a^^,^^b^.

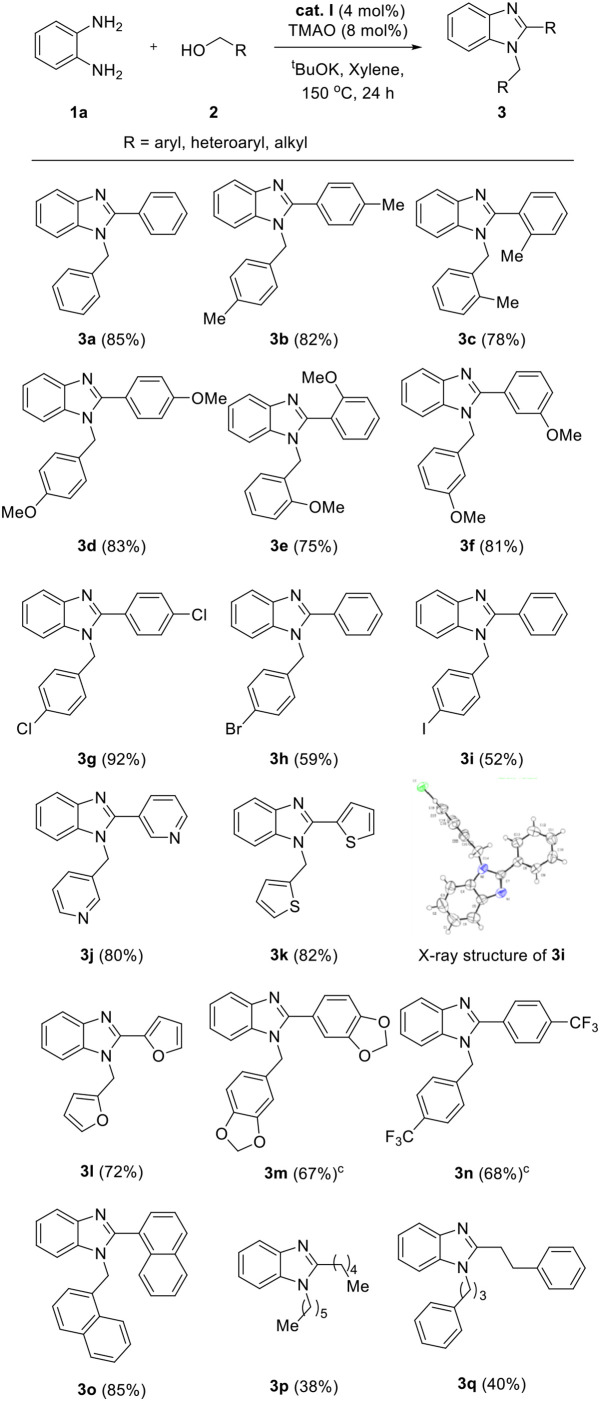

a *Reaction conditions: **1a** (0.5 mmol), **2** (1.5 mmol), ^t^BuOK (0.75 mmol), **cat. I** (0.02 mmol), TMAO (0.04 mmol), and xylene (2 ml) in a Schlenk flask under N_2_, 24 h, 150°C*.

b *Isolated yield in parentheses*.

*^c^Reaction time: 30 h*.

**Table 4:** Scope of diamines^a^^,^^b^.

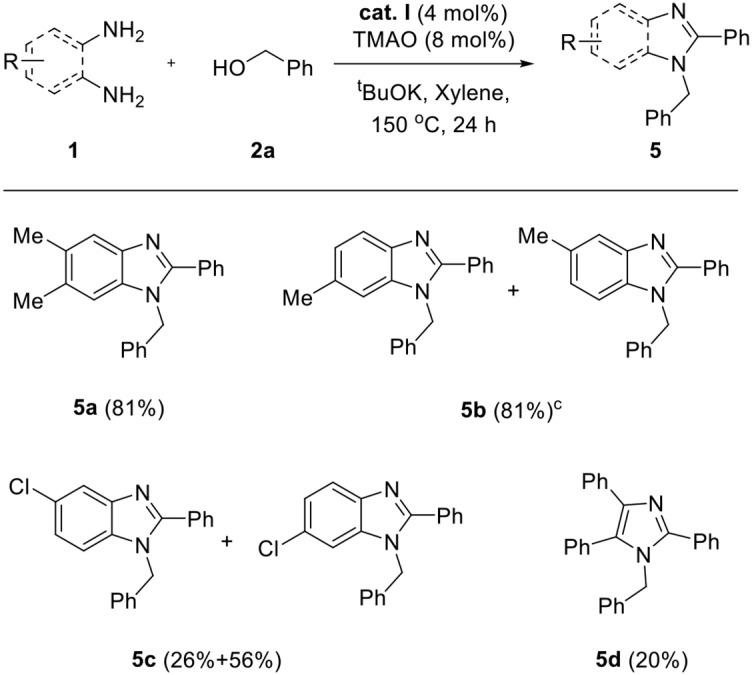

a *Reaction conditions: **1** (0.5 mmol), **2a** (1.5 mmol), ^t^BuOK (0.75 mmol), **cat. I** (0.02 mmol), TMAO (0.04 mmol), and xylene (2 ml) in Schlenk flasks under N_2_, 24 h, 150°C*.

b *Isolated yield in parentheses*.

*^c^Mixture could not be isolated*.

**Scheme 3:**
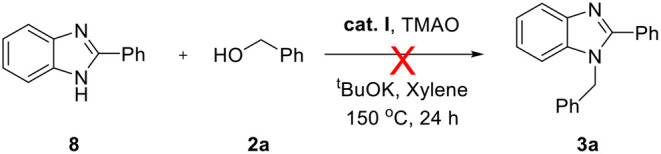
Failure on *N*-alkylation of benzimidazole.

**Scheme 4:**
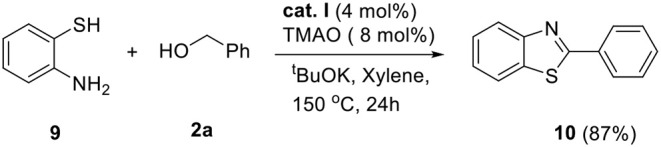
Synthesis of 2-phenylbenzo[*d*]thiazole. Reaction conditions: **9** (0.5 mmol), **2a** (1.5 mmol), ^*t*^BuOK (0.75 mmol), cat. I (0.02 mmol), TMAO (0.04 mmol), and xylene (2 ml) in Schlenk flasks under N_2_, 24 h, 150°C.

The above successful results led us to further investigate the reaction generality. *N*-Benzyl-1,2-diaminobenzene **6** was designed for the selective introduction of a substituent on the *N*-1 or *C*-2 position of benzimidazole. As shown in Table 5, benzylic alcohol **2** usually participates in the annulation process and is located on *C*-2 and its substituents on the benzimidazole product (**7a**–**e**). In contrast, five-membered heteroaromatic methyl alcohols gave *N*-1-heteroarylmethyl-*C*-2-phenyl-benzoimidazole products (**7f** and **7g**). This opposite selectivity is expected to depend on the electron density of the aromatic group. Furthermore, we applied the iron complex to achieve the direct *N*-alkylation of benzimidazole **8** with **2a** (Scheme 3). Unfortunately, no desired product was observed; however, this result suggests that the reaction mechanism did not proceed through benzimidazole as an intermediate. Besides benzimidazole, 2-phenyl benzothiazole **10** was also successfully synthesized in high yield (87%) using 2-aminobenzenethiol **9** under optimized reaction conditions (Scheme 4).

**Table 5:** Synthesis of 1,2-disubstituted benzimidazoles from **6**^a^^,^^b^.

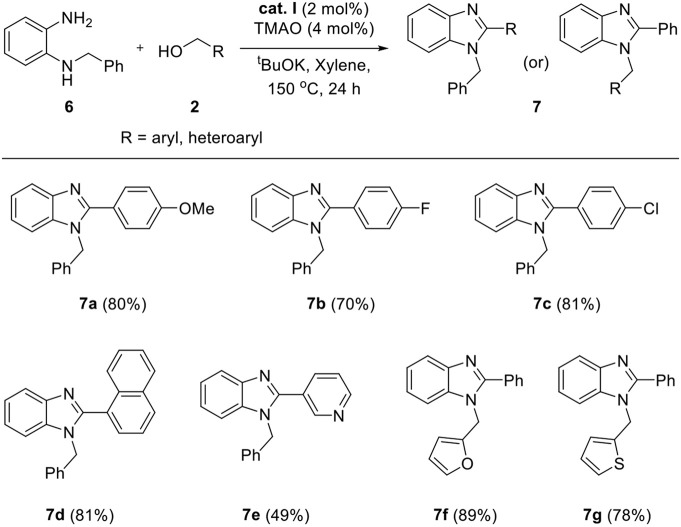

a *Reaction conditions: **6** (0.25 mmol), **2** (0.325 mmol), ^t^BuOK (0.25 mmol), **cat. I** (0.005 mmol), TMAO (0.01 mmol), and xylene (2 ml) in a Schlenk flask under N_2_, 24 h, 150°C*.

b *Isolated yield in parentheses*.

**Scheme 5:**
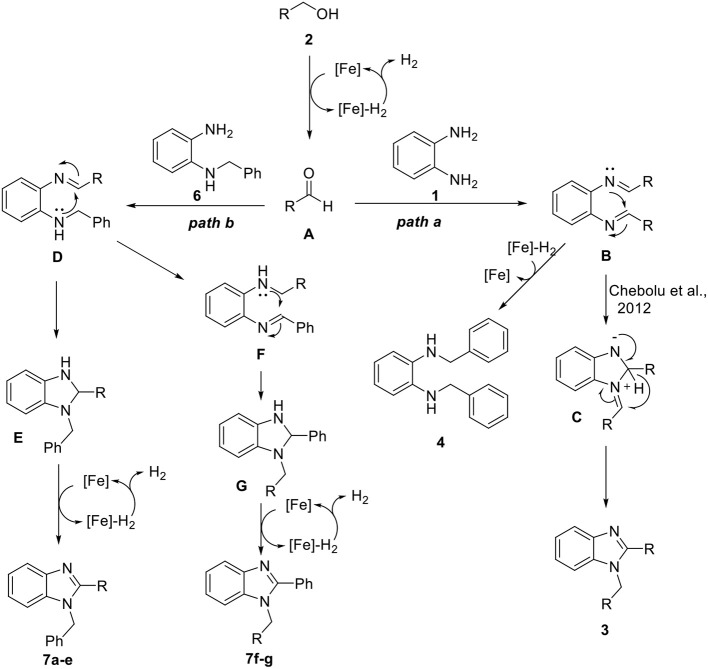
Plausible mechanism for the synthesis of 1,2-disubstituted benzimidazoles.

Based on the above observations and previous reports (Xu et al., 2017, 2018; Das et al., 2018), we proposed a plausible mechanism as shown in Scheme 5. Initially, aldehyde **A** was generated from alcohol *via* iron-catalyzed dehydrogenation. Then, the formation of bisimine intermediate **B** took place through the condensation of diamine **1** with aldehyde **A**. Bisimine **B** underwent intramolecular cyclization, followed by rearrangement to give 1,2-disubstituted benzimidazole **3** (Path a) (Chebolu et al., 2012). As mentioned in Table 2, we also identified diamine **4** as a side product, which might be generated from bisimine **B** through Fe-H_2_-mediated hydrogenation. On the other hand, *N*-benzyl-1,2-diaminobenzene **6** also reacted with the aldehyde **A** and generated imine intermediate **D** (Path b). Cyclic intermediate **E** was formed by an intramolecular nucleophilic attack, followed by iron-catalyzed dehydrogenation and aromatization, leading to the formation of 1,2-disubstituted benzimidazoles (**7a**–**e**). In the case of alcohols substituted with electron-rich heteroaromatic groups, such as furan and thiophene, an intramolecular nucleophilic attack of **D** might be less favored. Thus, it is rearranged quickly to intermediate **F**, and a regioisomer (**7f** and **7g**) was produced following a similar process.

## Conclusion

In conclusion, we have reported the first iron-catalyzed synthesis of 1,2-disubstituted benzimidazoles using alcohol oxidation-level substrates *via* the ADC strategy. The Knölker-type catalysts, tricarbonyl (η^4^-cyclopentadienone) iron complexes, were successfully employed in the dehydrogenative coupling of alcohol with 1,2-diaminobenzene, followed by annulation to give the 1,2-disubstituted benzimidazole products in good yields. Under the developed conditions, the reaction of *N*-benzyl-1,2-diaminobenzene with alcohols also provided 1,2-disubstituted benzimidazoles, and the regioselectivity of the substituents depends on the electron density of the alcohol substrate. In addition to benzoimidazole, benzothiazole was also synthesized well using the developed method. Iron is an earth-abundant and low-toxicity metal, and water and hydrogen gas are liberated as by-products in the reaction. Therefore, this methodology provides an eco-friendly alternative for the selective synthesis of 1,2-disubstituted benzimidazoles. Further extension using the Knölker-type complex to access other types of *N*-heterocycles is under investigation in our research group.

## Data Availability Statement

All datasets generated for this study are included in the article/Supplementary Material.

## Author Contributions

RP conducted the most experiments and wrote the manuscript. SC prepared iron complex. SL conducted the initial experiments. D-CO reviewed and edited the manuscript. SH directed the project and co-wrote the manuscript.

## Conflict of Interest

The authors declare that the research was conducted in the absence of any commercial or financial relationships that could be construed as a potential conflict of interest.

## Supplementary Material

The supporting information and X-ray data for this article can be found online at: https://www.frontiersin.org/articles/10.3389/fchem.2020.00429/full#supplementary-material
