# Supplementary material for: Iron-Catalyzed Acceptorless Dehydrogenative Coupling of Alcohols With Aromatic Diamines: Selective Synthesis of 1,2-Disubstituted Benzimidazoles
Source: Front Chem. 2020 Jun 19;8:429. doi: 10.3389/fchem.2020.00429 (PMC7317090; doi:10.3389/fchem.2020.00429)

Iron-catalyzed acceptorless dehydrogenative coupling of alcohols with aromatic diamines: Selective synthesis of 1,2-disubstituted benzimidazoles

Ramachandra Reddy Putta^1^, Simin Chun^1,2^, Seok Beom Lee^1,2^, Dong-Chan Oh^1,3^ and Suckchang Hong^1,2*^

^1^BK 21 plus project, College of Pharmacy, Seoul National University, Seoul, Republic of Korea

^2^Research Institute of Pharmaceutical Sciences, College of Pharmacy, Seoul National University, Seoul, Republic of Korea

^3^Natural Products Research Institute, College of Pharmacy, Seoul National University, Seoul, Republic of Korea

*** Correspondence:**Suckchang Hong - schong17@snu.ac.kr

**Supporting Information**

**Table of contents**

I**.** List of substrates S2

II**.** NMR Data of 1-benzyl-2-aryl-1*H*-benzo[*d*]imidazoles (**3**) S3

III. General procedure for the synthesis of *N*-benzyl-1,2-diaminobenzene (**6**) S16

IV. General procedure for the synthesis of benzimidazoles (**7**) from **6** S17

V. NMR Data of 2-phenylbenzo[*d*]thiazole (**10**) S21

VI. References S22

VII. X-Ray data of 1-(4-Iodobenzyl)-2-phenyl-1*H*-benzo[*d*]imidazole (**3i)** S23

VIII. NMR spectra S25

**I. List of substrates**

Compound **6** was prepared according to literature **(**Chattopadhyay et al., 2006), remaining all substrates are commercially available.

**II. NMR Data of 1-benzyl-2-aryl-1*H*-benzo[*d*]imidazoles (3)**

1-Benzyl-2-phenyl-1*H*-benzo[*d*]imidazole (**3a**)

Following the general procedure with **1a** and **2a**, **3a** was obtained as white solid (121 mg, 85% yield). m.p. 128-130 ^o^C.^1^H-NMR (400 MHz, CDCl_3_) δ 7.88 (d, *J* = 7.8 Hz, 1H), 7.69 (dd, *J* = 7.5, 1.6 Hz, 2H), 7.46 (dd, *J* = 13.0, 5.7 Hz, 3H), 7.29-7.33 (m, 4H), 7.23 (q, *J* = 7.5 Hz, 2H), 7.11 (d, *J* = 6.9 Hz, 2H), 5.46 (s, 2H).^13^C-NMR (100 MHz, CDCl_3_) δ 154.24, 143.27, 136.46, 136.14, 130.15, 129.99, 129.31, 129.12, 128.83, 127.84, 126.02, 123.10, 122.74, 120.06, 110.61, 48.43. HRMS (FAB^+^) m/z calcd for C_20_H_17_N_2_ [M+H]^+^: 285.1392, found: 285.1382

1-(4-Methylbenzyl)-2-(p-tolyl)-1*H*-benzo[*d*]imidazole (**3b**)

Following the general procedure with **1a** and **2b**, **3b** was obtained as white solid (128 mg, 82% yield) m.p. 117-119.^1^H-NMR (400 MHz, CDCl_3_) δ ^1^H-NMR (401 MHz, CDCl_3_) δ 7.86 (d, *J* = 8.3 Hz, 1H), 7.58-7.60 (m, 2H), 7.25-7.32 (m, 3H), 7.21 (dd, *J* = 6.0, 0.9 Hz, 2H), 7.13 (d, *J* = 7.8 Hz, 2H), 7.00 (d, *J* = 8.3 Hz, 2H), 5.41 (s, 2H), 2.41 (s, 3H), 2.34 (s, 3H).^13^C-NMR (100 MHz, CDCl_3_) δ 154.45, 143.31, 140.11, 137.53, 136.21, 133.57, 129.80, 129.54, 129.26, 127.29, 125.99, 122.91, 122.63, 119.93, 110.61, 48.28, 21.55, 21.20. HRMS (FAB) m/z calcd for C_22_H_21_N_2_ [M+H]^+^ : 313.1706, found: 313.1705.

1-(2-Methylbenzyl)-2-(o-tolyl)-1*H*-benzo[*d*]imidazole (**3c**)

Following the general procedure with **1a** and **2c**, **3c** was obtained as white solid (122 mg, 78% yield). m.p. 139-141. ^1^H-NMR (400 MHz, CDCl_3_) δ 7.87 (d, *J* = 7.8 Hz, 1H), 7.11-7.37 (m, 9H), 7.03 (td, *J* = 7.1, 2.3 Hz, 1H), 6.64 (d, *J* = 7.3 Hz, 1H), 5.18 (s, 2H), 2.24 (s, 3H), 2.15 (s, 3H). ^13^C-NMR (100 MHz, CDCl_3_) δ 154.04, 143.24, 138.46, 135.14, 134.93, 134.14, 130.69, 130.48, 129.98, 129.93, 127.63, 126.48, 126.11, 125.74, 122.92, 122.46, 120.15, 110.65, 45.86, 19.93, 19.20. HRMS (FAB^+^) m/z calcd for C_22_H_21_N_2_ [M+H]^+^ : 313.1705, found: 313.1716.

1-(4-Methoxybenzyl)-2-(4-methoxyphenyl)-1*H*-benzo[*d*]imidazole (**3d**)

Following the general procedure with **1a** and **2d**, **3d** was obtained as white solid (143 mg, 84% yield). m.p.131-133^o^C^1^H-NMR (400 MHz, CDCl_3_) δ 7.84 (d, *J* = 7.8 Hz, 1H), 7.64 (dd, *J* = 6.6, 2.1 Hz, 2H), 7.27-7.31 (m, 1H), 7.21-7.22 (m, 2H), 7.04 (d, *J* = 8.7 Hz, 2H), 6.97 (dd, *J* = 6.9, 2.3 Hz, 2H), 6.86 (dd, *J* = 6.6, 2.1 Hz, 2H), 5.38 (s, 2H), 3.85 (s, 3H), 3.78 (s, 3H). ^13^C-NMR (100 MHz, CDCl_3_) δ 160.99, 159.20, 154.25, 143.29, 136.21, 130.82, 128.60, 127.32, 122.85, 122.64, 122.55, 119.84, 114.53, 114.30, 110.54, 55.50, 55.42, 48.00. HRMS (FAB^+^) m/z calcd for C_22_H_21_N_2_O_2_ [M+H]^+^ : 345.1603, found: 345.1607.

1-(2-Methoxybenzyl)-2-(2-methoxyphenyl)-1*H*-benzo[*d*]imidazole (**3e**)

Following the general procedure with **1a** and **2e**, **3e** was obtained as white solid (129 mg ,75% yield). m.p. 152-154 ^o^C. ^1^H-NMR (400 MHz, CDCl_3_) δ 7.84 (d, *J* = 7.8 Hz, 1H), 7.53 (dd, *J* = 7.3, 1.8 Hz, 1H), 7.44 (t, *J* = 7.8 Hz, 1H), 7.23-7.27 (m, 2H), 7.16-7.20 (m, 2H), 7.04 (t, *J* = 7.3 Hz, 1H), 6.95 (d, *J* = 8.2 Hz, 1H), 6.83 (d, *J* = 8.2 Hz, 1H), 6.76 (t, *J* = 7.5 Hz, 1H), 6.68 (d, *J* = 7.3 Hz, 1H), 5.23 (s, 2H), 3.78 (s, 3H), 3.58 (s, 3H). ^13^C-NMR (100 MHz, CDCl_3_) δ 157.69, 156.58, 152.57, 143.46, 135.64, 132.49, 131.51, 128.47, 127.82, 124.65, 122.55, 122.03, 120.88, 120.48, 119.92, 110.90, 110.87, 110.00, 55.31, 55.23, 43.64. HRMS (FAB^+^) m/z calcd for C_22_H_21_N_2_O_2_ [M+H]^+^ : 345.1603, found: 345.1610.

1-(3-Methoxybenzyl)-2-(3-methoxyphenyl)-1*H*-benzo[*d*]imidazole (**3f**)

Following the general procedure with **1a** and **2f**, **3f** was obtained as white solid (139 mg, 81% yield). ^1^H-NMR (400 MHz, CDCl_3_) δ 7.84-7.86 (m, 1H), 7.28-7.35 (m, 2H), 7.21-7.25 (m, 5H), 6.98-7.01 (dq, *J* = 8.2, 1.2 Hz, 1H), 6.81 (dd, *J* = 8.0, 2.1 Hz, 1H), 6.67-6.69 (m, 1H), 6.65 (d, *J* = 1.8 Hz, 1H), 5.42 (s, 2H), 3.71 (d, *J* = 1.4 Hz, 6H). ^13^C-NMR (100 MHz, CDCl_3_) δ 160.26, 159.84, 154.13, 143.15, 138.25, 136.26, 131.31, 130.29, 129.91, 123.24, 122.83, 121.53, 120.08, 118.27, 116.68, 114.08, 112.98, 111.94, 110.58, 55.35, 55.32, 48.43. HRMS (FAB^+^) m/z calcd for C_22_H_21_N_2_O_2_ [M+H]^+^ : 345.1603, found: 345.1598.

1-(4-Chlorobenzyl)-2-(4-chlorophenyl)-1*H*-benzo[*d*]imidazole (**3g**)

Following the general procedure with **1a** and **2g**, **3g** was obtained as white solid (162 mg, 92% yield). m. p. 138-140 ^o^C.^1^H-NMR (400 MHz, CDCl_3_) δ 7.87 (d, *J* = 8.2 Hz, 1H), 7.59 (dd, *J* = 6.9, 1.8 Hz, 2H), 7.44 (dd, *J* = 8.5, 2.1 Hz, 2H), 7.30-7.34 (m, 3H), 7.26-7.27 (m, 1H), 7.20 (d, *J* = 7.8 Hz, 1H), 7.02 (d, *J* = 8.2 Hz, 2H), 5.40 (s, 2H). ^13^C-NMR (100 MHz, CDCl_3_) δ 153.02, 143.24, 136.47, 136.06, 134.77, 133.97, 130.56, 129.52, 129.29, 128.49, 127.37, 123.60, 123.17, 120.34, 110.43, 47.92. HRMS (FAB^+^) m/z calcd for C_20_H_15_N_2_Cl_2_ [M+H]^+^: 353.0612, found: 353.0606.

1-(4-Bromobenzyl)-2-phenyl-1*H*-benzo[*d*]imidazole (**3h**)

Following the general procedure with **1a** and **2h**, **3h** was obtained as white solid (107 mg, 59% yield). m.p. 157-169^o^C.^1^H-NMR (400 MHz, CDCl_3_) δ 7.88 (d, *J* = 7.8 Hz, 1H), 7.66 (dd, *J* = 7.8, 1.8 Hz, 2H), 7.45-7.49 (m, 4H), 7.30-7.32 (m, 1H), 7.23-7.26 (m, 2H), 7.18 (d, *J* = 8.2 Hz, 1H), 6.98 (d, *J* = 8.2 Hz, 2H), 5.40 (s, 2H). ^13^C-NMR (100 MHz, CDCl_3_) δ 154.23, 143.34, 135.97, 135.55, 132.37, 130.18, 130.05, 129.33, 128.98, 127.82, 123.34, 123.00, 121.87, 120.29, 110.44, 47.99. HRMS (FAB^+^) m/z calcd for C_20_H_16_N_2_Br [M+H]^+^: 363.0497, found: 363.0493.

1-(4-Iodobenzyl)-2-phenyl-1*H*-benzo[*d*]imidazole (**3i**)

Following the general procedure with **1a** and **2i**, **3i** was obtained as white solid (107 mg, 52% yield). 181-183 ^o^C.^1^H-NMR (400 MHz, CDCl_3_) δ 7.87 (d, *J* = 7.8 Hz, 1H), 7.64-7.67 (m, 4H), 7.45-7.48 (m, 3H), 7.32 (td, *J* = 7.6, 1.2 Hz, 1H), 7.25-7.26 (m, 1H), 7.18 (d, *J* = 7.8 Hz, 1H), 6.85 (d, *J* = 8.7 Hz, 2H), 5.39 (s, 2H). ^13^C-NMR (100 MHz, CDCl_3_) δ 154.23, 143.31, 138.29, 136.23, 135.96, 130.18, 130.01, 129.30, 128.97, 128.01, 123.33, 122.99, 120.26, 110.44, 93.36, 48.05. HRMS (FAB^+^) m/z calcd for C_20_H_16_IN_2_ [M+H]^+^: 411.0358, found: 411.0360.

2-(Pyridin-3-yl)-1-(pyridin-3-ylmethyl)-1*H*-benzo[*d*]imidazole (**3j**) (Samanta et al., 2018)

Following the general procedure with **1a** and **2j**, **3j** was obtained as yellow solid (Column chromatography: 1% of methanol in dichloromethane) (115 mg, 80% yield). m.p. 128-130 ^o^C.^1^H-NMR (400 MHz, CDCl_3_) δ 8.91 (s, 1H), 8.75 (d, *J* = 4.1 Hz, 1H), 8.57 (d, *J* = 3.7 Hz, 1H), 8.47 (s, 1H), 8.01 (dt, *J* = 7.8, 1.8 Hz, 1H), 7.90 (d, *J* = 7.8 Hz, 1H), 7.44 (q, *J* = 4.1 Hz, 1H), 7.24-7.37 (m, 5H), 5.50 (s, 2H).^13^C-NMR (100 MHz, CDCl_3_) δ 151.19, 151.04, 149.79, 149.65, 147.92, 143.38, 136.85, 135.86, 133.70, 131.66, 126.40, 124.10, 124.05, 123.85, 123.47, 120.61, 110.38, 46.27. HRMS (FAB^+^) m/z calcd for C_18_H_15_N_4_ [M+H]^+^: 287.1297, found: 287.1303.

2-(Thiophen-2-yl)-1-(thiophen-2-ylmethyl)-1*H*-benzo[*d*]imidazole (**3k**)

Following the general procedure with **1a** and **2k**, **3k** was obtained as white solid (122 mg, 82% yield). m.p. 151-153^o^C. ^1^H-NMR (400 MHz, CDCl_3_) δ 7.84 (dd, *J* = 6.7, 2.5 Hz, 1H), 7.53 (dd, *J* = 5.1, 0.9 Hz, 1H), 7.48 (dd, *J* = 3.7, 0.9 Hz, 1H), 7.37-7.39 (m, 1H), 7.28-7.32 (m, 1H), 7.24-7.26 (m, 1H), 7.15 (dd, *J* = 5.1, 3.7 Hz, 1H), 6.95 (dd, *J* = 5.3, 3.4 Hz, 1H), 6.87-6.88 (m, 1H), 5.72 (s, 1H). ^13^C-NMR (100 MHz, CDCl_3_) δ 147.68, 143.04, 138.91, 135.96, 131.87, 129.05, 128.15, 128.03, 127.34, 125.59, 125.53, 123.44, 123.12, 120.00, 110.02, 44.18. HRMS (FAB^+^) m/z calcd for C_16_H_13_N_2_S_2_ [M+H]^+^: 297.0520, found: 297.0515.

2-(Furan-2-yl)-1-(furan-2-ylmethyl)-1*H*-benzo[*d*]imidazole (**3l**)

Following the general procedure with **1a** and **2l**, **3l** was obtained as brown solid (95 mg, 72% yield). m.p. 94-96 ^o^C. ^1^H-NMR (400 MHz, CDCl_3_) δ 7.77-7.80 (m, 1H), 7.65 (s, 1H), 7.50-7.52 (m, 1H), 7.29-7.34 (m, 3H), 7.21-7.22 (m, 1H), 6.62 (q, *J* = 1.5 Hz, 1H), 6.29 (t, *J* = 2.5 Hz, 1H), 6.25 (d, J = 2.7 Hz, 1H), 5.65 (s, 2H). ^13^C-NMR (100 MHz, CDCl_3_) δ 149.69, 145.50, 144.08, 144.03, 143.08, 142.77, 135.59, 123.33, 123.02, 119.92, 112.98, 112.18, 110.64, 110.09, 108.48, 41.77. HRMS (FAB^+^) m/z calcd for C_16_H_13_N_2_O_2_ [M+H]^+^: 265.0977, found: 265.0980.

1-(3',4'-Methylenedioxybenzyl)-2-(3'',4''-methylenedioxyphenyl)-1*H*-benzimidazole (**3m**)

Following the general procedure with **1a** and **2m**, **3m** was obtained as white solid (125 mg, 67% yield). m.p.171-173. ^1^H-NMR (400 MHz, CDCl_3_) δ 7.83 (d, J = 7.8 Hz, 1H), 7.27-7.32 (m, 1H), 7.15-7.23 (m, 4H), 6.88 (d, *J* = 7.8 Hz, 1H), 6.75 (d, *J* = 8.7 Hz, 1H), 6.56-6.57 (m, 2H), 6.03 (s, 2H), 5.94 (s, 2H), 5.35 (s, 2H). ^13^C-NMR (100 MHz, CDCl_3_) δ 153.90, 149.23, 148.51, 148.13, 147.34, 143.15, 136.12, 130.25, 123.86, 123.70, 123.09, 122.81, 119.96, 119.37, 110.56, 109.76, 108.82, 108.72, 106.64, 101.68, 101.41, 48.29. HRMS (FAB^+^) m/z calcd for C_22_H_17_N_2_O_4_ [M+H]^+^: 373.1188, found: 373.1199.

1-(4-(Trifluoromethyl)benzyl)-2-(4-(trifluoromethyl)phenyl)-1*H*-benzo[*d*]imidazole (**3n**)

Following the general procedure with **1a** and **2n**, **3n** was obtained as white solid (141 mg, 68% yield). m.p. 145-147 ^o^C. ^1^H-NMR (400 MHz, CDCl_3_) δ 7.91 (d, *J* = 7.8 Hz, 1H), 7.78 (d, *J* = 8.2 Hz, 2H), 7.73 (d, *J* = 8.2 Hz, 2H), 7.62 (d, *J* = 8.2 Hz, 2H), 7.35-7.37 (m, 1H), 7.31 (td, *J* = 7.7, 1.1 Hz, 1H), 7.21 (dd, *J* = 7.3, 6.4 Hz, 3H), 5.51 (s, 2H). ^13^C-NMR (10 MHz, CDCl_3_) δ 152.53, 143.25, 140.12, 136.06, 133.43, 129.65, 126.45 (d, *J*_C, F_ = 3.6 Hz, 1C), 126.31, 126.03 (d, *J*_C, F_ = 3.8 Hz, 1C), 124.07, 123.52, 120.62, 110.44, 48.17. HRMS (FAB^+^) m/z calcd for C_22_H_15_F_6_N_2_ [M+H]^+^: 421.1139, found: 421.1132.

2-(Naphthalen-1-yl)-1-(naphthalen-1-ylmethyl)-1*H*-benzo[*d*]imidazole (**3o**)

Following the general procedure with **1a** and **2o**, **3o** was obtained as white solid (163 mg, 85% yield). m.p.148-150 ^o^C. ^1^H-NMR (400 MHz, CDCl_3_) δ 7.99 (t, *J* = 8.0 Hz, 2H), 7.85-7.93 (m, 3H), 7.75 (q, *J* = 4.0 Hz, 2H), 7.44-7.56 (m, 4H), 7.38 (t, *J* = 7.5 Hz, 2H), 7.23-7.30 (m, 4H), 6.84 (d, *J* = 6.9 Hz, 1H), 5.73 (s, 2H). ^13^C-NMR (100 MHz, CDCl_3_) δ 153.26, 143.51, 135.50, 133.76, 133.66, 132.38, 131.32, 130.51, 130.23, 129.07, 128.46, 128.30, 127.49, 127.36, 126.60, 126.53, 126.11, 125.70, 125.56, 124.95, 123.63, 123.24, 122.77, 122.18, 120.37, 110.89, 46.25. HRMS (FAB^+^) m/z calcd for C_28_H_21_N_2_ [M+H]^+^: 385.1705, found: 385.1708.

1-Hexyl-2-pentyl-1*H*-benzo[*d*]imidazole (**3p**)

Following the general procedure with **1a** and **2p**, **3p** was obtained as brown oil (52 mg, 38% yield). ^1^H-NMR (400 MHz, CDCl_3_) δ 7.71-7.73 (m, 1H), 7.25-7.28 (m, 1H), 7.20-7.22 (m, 2H), 4.09 (t, *J* = 7.5 Hz, 2H), 2.85 (t, *J* = 7.8 Hz, 2H), 1.90 (q, *J* = 7.6 Hz, 2H), 1.78 (q, *J* = 7.5 Hz, 2H), 1.31-1.42 (m, 10H), 0.86-0.94 (m, 6H). ^13^C-NMR (100 MHz, CDCl_3_) δ 155.23, 142.86, 135.14, 121.90, 121.71, 119.30, 109.33, 43.79, 31.92, 31.55, 30.00, 27.69, 27.64, 26.78, 22.63, 22.59, 14.13, 14.10. HRMS (FAB^+^) m/z calcd for C_18_H_29_N_2_ [M+H]^+^: 273.2331, found: 273.2329.

2-Phenethyl-1-(3-phenylpropyl)-1*H*-benzo[*d*]imidazole (**3q**)

Following the general procedure with **1a** and **2q**, **3q** was obtained as brown oil (68 mg, 40% yield).^1^H-NMR (400 MHz, CDCl_3_) δ 7.76 (q, *J* = 2.9 Hz, 1H), 7.15-7.30 (m, 13H), 3.98 (t, *J* = 7.5 Hz, 2H), 3.21 (t, *J* = 8.0 Hz, 2H), 3.05 (t, *J* = 8.0 Hz, 2H), 2.66 (t, *J* = 7.3 Hz, 2H), 2.05 (t, *J* = 7.5 Hz, 2H). ^13^C-NMR (100 MHz, CDCl_3_) δ 154.16, 142.83, 140.99, 140.39, 135.00, 128.71, 128.51, 128.37, 126.49, 122.18, 121.96, 119.38, 109.36, 42.86, 34.21, 33.02, 31.02, 29.73.HRMS (FAB^+^) m/z calcd for C_24_H_25_N_2_ [M+H]^+^ : 341.2018, found: 341.2019.

*N*_1_,*N*_2_-Dibenzylbenzene-1,2-diamine (**4**)

Following the general procedure with **1a, 2a**, cat. I (0.025 mmol) and TMAO (0.05 mmol), **4** was obtained as colorless oil (72 mg, 50% yield).^1^H-NMR (400 MHz, CDCl_3_) δ 7.33-7.41 (m, 8H), 7.26-7.30 (m, 2H), 6.78-6.82 (m, 2H), 6.72-6.74 (m, 2H), 4.33 (s, 4H), 3.65 (s, 2H). ^13^C-NMR (100 MHz, CDCl_3_) δ 139.51, 137.25, 128.74, 127.96, 127.39, 119.55, 112.07, 48.92. HRMS (FAB^+^) m/z calcd for C_20_H_20_N_2_ [M+H]^+^: 288.1626, found: 288.1638.

1-Benzyl-5,6-dimethyl-2-phenyl-1*H*-benzo[*d*]imidazole (**5a**)

Following the general procedure with **1b and 2a**, **5a** was obtained as white solid (126 mg, 81% yield). m.p. 191-193 ^o^C.^1^H-NMR (400 MHz, CDCl_3_) δ 7.62-7.67 (m, 3H), 7.42-7.44 (m, 3H), 7.31-7.34 (m, 3H), 7.10-7.12 (m, 2H), 6.97 (s, 1H), 5.41 (s, 2H), 2.39 (s, 3H), 2.33 (s, 3H). ^13^C-NMR (100 MHz, CDCl_3_) δ 153.49, 141.94, 136.85, 134.83, 132.35, 131.67, 130.47, 129.75, 129.29, 129.16, 128.79, 127.76, 126.02, 120.15, 110.68, 48.38, 20.72, 20.46. HRMS (FAB^+^) m/z calcd for C_22_H_21_N_2_ [M+H]^+^ : 313.1705, found: 313.1701.

1-Benzyl-6-methyl-2-phenyl-1*H*-benzo[*d*]imidazole (**5b**) (Bera et al., 2019)

Following the general procedure with **1c and 2a**, **5b+5b`** was obtained as white solid (121 mg, 81% yield). m.p. 188-190 ^o^C. ^1^H-NMR (400 MHz, CDCl_3_) δ 7.74 (d, *J* = 8.2 Hz, 1H), 7.67 (td, *J* = 3.8, 2.1 Hz, 2H), 7.42-7.45 (m, 3H), 7.30-7.34 (m, 3H), 7.12 (t, *J* = 8.2 Hz, 3H), 7.00 (s, 1H), 5.42 (s, 2H), 2.44 (s, 3H). ^13^C-NMR (100 MHz, CDCl_3_) δ 153.87, 141.45, 136.73, 136.51, 133.22, 130.37, 129.87, 129.37, 129.32, 129.20, 128.83, 127.83, 126.10, 126.05, 124.62, 124.42, 119.92, 119.65, 110.42, 110.16, 48.51, 48.38, 22.00, 21.74. HRMS (FAB^+^) m/z calcd for C_21_H_19_N_2_ [M+H]^+^ : 299.1548, found: 299.1551.

1-Benzyl-5-chloro-2-phenyl-1*H*-benzo[*d*]imidazole (**5c**) (Kommi et al., 2012).

Following the general procedure with **1d and 2a**, **5c** was obtained as white solid (42 mg, 26% yield). m.p. 178-180^o^C. ^1^H-NMR (400 MHz, CDCl_3_) δ 7.83 (d, J = 1.8 Hz, 1H), 7.66-7.68 (m, 2H), 7.47 (dd, *J* = 7.4, 1.4 Hz, 3H), 7.32 (q, *J* = 7.5 Hz, 3H), 7.19 (dd, *J* = 8.7, 1.8 Hz, 1H), 7.09 (t, *J* = 9.0 Hz, 3H), 5.44 (s, 2H). ^13^C-NMR (100 MHz, CDCl_3_) δ 155.13, 141.96, 136.84, 135.95, 130.29, 129.76, 129.34, 129.32, 128.97, 128.83, 128.13, 126.00, 123.54, 120.99, 110.67, 48.61. HRMS (FAB^+^) m/z calcd for C_20_H_16_ClN_2_ [M+H]^+^: 319.1002, found: 319.0998.

1-Benzyl-6-chloro-2-phenyl-1*H*-benzo[*d*]imidazole (**5c`**) (Bera et al., 2019)

Following the general procedure with **1d and 2a**, **5c`** was obtained as white solid (90 mg, 56% yield). m.p. 168-170 ^o^C. ^1^H-NMR (400 MHz, CDCl_3_) δ 7.75 (d, J = 8.7 Hz, 1H), 7.64-7.67 (m, 2H), 7.44-7.46 (m, 3H), 7.33-7.34 (m, 3H), 7.26 (dd, J = 8.9, 2.1 Hz, 2H), 7.18 (d, J = 1.8 Hz, 1H), 7.06-7.08 (m, 2H), 5.41 (s, 2H).^13^C-NMR (101 MHz, CDCl_3_) δ 155.50, 144.14, 136.06, 134.77, 130.34, 129.78, 129.36, 129.28, 128.99, 128.39, 128.10, 126.03, 123.57, 119.90, 111.46, 48.65. HRMS (FAB^+^) m/z calcd for C_20_H_16_ClN_2_ [M+H]^+^: 319.1002, found: 319.1003.

1-Benzyl-2,4,5-triphenyl-1*H*-imidazole (**5d**) (Sun et al., 2019)

Following the general procedure with **1e and 2a**, **5d** was obtained as white solid (93 mg, 20% yield). m.p. 158-160 ^o^C. ^1^H-NMR (400 MHz, CDCl_3_) δ 7.64-7.66 (m, 2H), 7.56-7.58 (m, 2H), 7.39 (td, *J* = 5.4, 3.4 Hz, 3H), 7.31-7.35 (m, 3H), 7.13-7.22 (m, 8H), 6.79-6.81 (m, 2H), 5.11 (s, 2H). ^13^C-NMR (100MHz, CDCl_3_) δ 148.19, 138.16, 137.67, 134.55, 131.21, 131.14, 130.18, 129.21, 129.06, 128.93, 128.76, 128.74, 128.71, 128.21, 127.48, 126.91, 126.51, 126.14, 48.41. HRMS (FAB^+^) m/z calcd for C_28_H_23_N_2_ [M+H]^+^: 387.1861, found: 387.1855.

**III. General procedure for the synthesis of *N*-benzyl 1,2-diaminobenzene** (**6)**. **(**Chattopadhyay et al., 2006).

In a round bottomed flask, 1,2-diaminobenzene **1a** (15 mmol) and K_2_CO_3_ (30.0 mmol) were dissolved with methanol (50 mL). To this, benzyl bromide (10.0 mmol) was added dropwise. The resulting mixture was stirred at room temperature for 4 h and then diluted with ethyl acetate (50 mL). The reaction solution was washed with saturated NaHCO_3_ and saturated NaCl, dried over anhydrous Na_2_SO_4_, and filtered, and the filtrate was concentrated in vacuo. The residual oil was purified by silica gel column chromatography.

***N*-Benzyl-1,2-diaminobenzene** (**6)**

Brown oil. ^1^H-NMR (400 MHz, CDCl_3_) δ 7.27-7.38 (m, 5H), 6.69-6.82 (m, 4H), 4.30 (s, 2H), 3.69 (br, 1H), 3.34 (br, 2H).

**IV. General procedure for the synthesis of benzimidazoles (7) from 6**

In a 15 mL Schlenk flask, a mixture of N-benzyl-1,2-diaminobenzene **6** (0.25 mmol.), alcohol **2** (0.325 mmol), ^t^BuOK (0.25 mmol), cat. I (0.005 mmol) and TMAO (0.01 mmol) was stirred at 150 ^o^C in xylene (2 mL) for 24 h, under nitrogen atmosphere. After cooling, dichloromethane was added to dilute, and the solvent was removed under reduced pressure. The crude product was purified by column chromatography on silica gel using 10−30% ethyl acetate in hexane as an eluent.

1-Benzyl-2-(4-methoxyphenyl)-1*H*-benzo[*d*]imidazole (**7a**) (Das et al., 2018)

Following the general procedure with **6** and **2d**, **7a** was obtained as white solid (63 mg, 80% yield). m.p. 131-133^o^C. ^1^H-NMR (400 MHz, CDCl_3_) δ 7.85 (d, *J* = 7.8 Hz, 1H), 7.63 (dd, *J* = 6.9, 1.8 Hz, 2H), 7.30-7.34 (m, 4H), 7.20-7.22 (m, 2H), 7.12 (d, *J* = 6.9 Hz, 2H), 6.96 (dd, *J* = 6.9, 2.3 Hz, 2H), 5.45 (s, 2H), 3.84 (s, 3H). ^13^C-NMR (100 MHz, CDCl_3_) δ 161.00, 154.29, 143.31, 136.63, 136.23, 130.78, 129.18, 127.84, 126.04, 122.87, 122.67, 122.48, 119.86, 114.29, 110.46, 55.46, 48.47. HRMS (FAB^+^) m/z calcd for C_21_H_19_N_2_O [M+H]^+^: 315.1497, found: 315.1499.

1-Benzyl-2-(4-fluorophenyl)-1*H*-benzo[*d*]imidazole (**7b**) (Xie et al., 2017)

Following the general procedure with **6** and **2r**, **7b** was obtained as white solid (53 mg, 70% yield). m.p. 119-121^o^C. ^1^H-NMR (400 MHz, CDCl_3_) δ 7.86 (d, *J* = 7.8 Hz, 1H), 7.64-7.68 (m, 2H), 7.32-7.34 (m, 4H), 7.23-7.26 (m, 2H), 7.10-7.16 (m, 4H), 5.44 (s, 2H).^13^C-NMR (100 MHz, CDCl_3_) δ 165.11 (d, ^1^*J*_C, F_ = 248.6 Hz, 1C), 153.32, 143.20, 136.38, 136.22, 131.39 (d, ^3^*J*_C, F_ = 8.6 Hz, 1C), 129.28, 128.02, 126.38 (d, ^4^*J*_C, F_ = 2.8 Hz, 1C), 126.02, 123.32, 122.94, 120.13, 116.09 (d, ^2^*J*_C, F_ = 21.9 Hz, 1C), 110.61, 48.48. HRMS (FAB^+^) m/z calcd for C_20_H_16_FN_2_ [M+H]^+^ : 303.1298, found: 303.1296.

1-Benzyl-2-(4-chlorophenyl)-1*H*-benzo[*d*]imidazole (**7c**) (Xie et al., 2017)

Following the general procedure with **6** and **2g**, **7c** was obtained as white solid (65 mg, 81% yield). m.p. 135-137^o^C. ^1^H-NMR (400 MHz, CDCl_3_) δ 7.87 (d, *J* = 8.3 Hz, 1H), 7.61-7.63 (m, 2H), 7.41-7.43 (m, 2H), 7.30-7.34 (m, 4H), 7.23-7.24 (m, 2H), 7.08-7.10 (m, 2H), 5.44 (s, 2H). ^13^C-NMR (100 MHz, CDCl_3_) δ 153.09, 143.19, 136.35, 136.30, 130.65, 129.29, 129.21, 128.64, 128.05, 125.98, 123.46, 123.02, 120.18, 110.63, 48.51. HRMS (FAB^+^) m/z calcd for C_20_H_16_ClN_2_ [M+H]^+^: 319.1002, found: 319.1015.

1-Benzyl-2-(naphthalen-1-yl)-1*H*-benzo[*d*]imidazole (**7d**) (Lin et al., 2014)

Following the general procedure with **6** and **2o**, **7d** was obtained as white solid (68 mg, 81% yield). m.p. 109-111^o^C. ^1^H-NMR (400 MHz, CDCl_3_) δ 7.96 (d, *J* = 7.8 Hz, 1H), 7.90 (d, *J* = 7.8 Hz, 2H), 7.78 (d, *J* = 8.2 Hz, 1H), 7.56 (d, *J* = 6.9 Hz, 1H), 7.47 (td, *J* = 16.1, 7.6 Hz, 3H), 7.22-7.32 (m, 3H), 7.17 (t, *J* = 3.0 Hz, 3H), 6.90 (t, *J* = 3.7 Hz, 2H), 5.19 (s, 2H). ^13^C-NMR (100 MHz, CDCl_3_) δ 153.02, 143.52, 136.21, 135.29, 133.67, 132.41, 130.49, 128.85, 128.81, 128.48, 127.80, 127.34, 126.60, 126.53, 125.58, 125.02, 123.13, 122.65, 120.31, 110.77, 48.38. HRMS (FAB^+^) m/z calcd for C_24_H_19_N_2_ [M+H]^+^ : 335.1548, found: 335.1542.

1-Benzyl-2-(pyridin-3-yl)-1*H*-benzo[*d*]imidazole (**7e**)

Following the general procedure with **6** and **2j**, **7e** was obtained as light-yellow solid (column chromatography eluent: 1% of methanol in dichloromethane) (35 mg, 49% yield). ^1^H-NMR (400 MHz, CDCl_3_) δ 8.56 (d, *J* = 3.7 Hz, 1H), 8.49 (d, *J* = 1.4 Hz, 1H), 7.88 (d, *J* = 7.8 Hz, 1H), 7.65 (dd, *J* = 7.4, 1.8 Hz, 2H), 7.47-7.59 (m, 2H), 7.22-7.33 (m, 6H), 5.48 (s, 2H). ^13^C-NMR (100 MHz, CDCl_3_) δ 154.16, 149.54, 148.11, 143.34, 135.76, 133.89, 132.15, 130.28, 129.97, 129.33, 129.06, 124.02, 123.47, 123.10, 120.39, 110.28, 46.21. HRMS (FAB^+^) m/z calcd for C_19_H_16_N_3_ [M+H]^+^: 286.1344, found: 286.1350.

1-(Furan-2-ylmethyl)-2-phenyl-1*H*-benzo[*d*]imidazole (**7f**)

Following the general procedure with **6** and **2l**, **7f** was obtained as white solid (61 mg, 89% yield). m.p. 128-130 ^o^C. ^1^H-NMR (400 MHz, CDCl_3_) δ 7.83 (d, *J* = 7.8 Hz, 1H), 7.57 (d, *J* = 1.4 Hz, 1H), 7.24-7.32 (m, 6H), 7.14 (d, *J* = 6.4 Hz, 2H), 7.07 (d, *J* = 3.7 Hz, 1H), 6.55 (q, *J* = 1.7 Hz, 1H), 5.71 (s, 2H). ^13^C-NMR (100 MHz, CDCl_3_) δ 145.30, 144.54, 144.13, 143.21, 136.44, 135.84, 128.98, 127.80, 126.31, 123.33, 122.98, 119.98, 112.75, 112.04, 110.06, 48.35. HRMS (FAB^+^) m/z calcd for C_18_H_15_N_2_O [M+H]^+^: 275.1184, found: 275.1185.

2-Phenyl-1-(thiophen-2-ylmethyl)-1*H*-benzo[*d*]imidazole (**7g**)

Following the general procedure with **6** and **2k**, **7g** was obtained as white solid (57 mg, 78% yield). m.p. 132-134 ^o^C. ^1^H-NMR (400 MHz, CDCl_3_) δ 7.86 (d, *J* = 7.8 Hz, 1H), 7.49 (dd, *J* = 5.0, 0.9 Hz, 1H), 7.28-7.34 (m, 4H), 7.25 (d, *J* = 5.0 Hz, 3H), 7.13 (d, *J* = 6.9 Hz, 2H), 7.08 (dd, *J* = 5.0, 3.7 Hz, 1H), 5.61 (s, 2H).^13^C-NMR (100 MHz, CDCl_3_) δ 148.20, 143.16, 136.43, 136.18, 132.23, 129.27, 128.90, 128.00, 127.96, 127.83, 125.94, 123.36, 123.03, 120.00, 110.07, 48.28. HRMS (FAB^+^) m/z calcd for C_18_H_15_N_2_S [M+H]^+^: 291.0956, found: 291.0959.

**V. NMR Data of 2-phenylbenzo[*d*]thiazole (10)**

2-phenylbenzo[*d*]thiazole (**10**)

Following the general procedure with 2-aminobenzenethiol **9** and **2a**, **10** was obtained as white solid (92 mg, 87% yield). m.p. 114-116 ^o^C. ^1^H-NMR (400 MHz, CDCl_3_) δ 8.07-8.11 (m, 3H), 7.90-7.92 (m, 1H), 7.48-7.52 (m, 4H), 7.37-7.41 (m, 1H). ^13^C-NMR (100 MHz, CDCl_3_) δ 168.17, 154.25, 135.17, 133.72, 131.09, 129.13, 127.67, 126.43, 125.30, 123.34, 121.73. HRMS (FAB^+^) m/z calcd for C_13_H_10_NS [M+H]^+^: 212.0534, found: 212.0538.

**VI. References**

Bera, A., Sk, M., Singh, K., and Banerjee, D. (2019). Nickel-catalysed dehydrogenative coupling of aromatic diamines with alcohols: selective synthesis of substituted benzimidazoles and quinoxalines. *Chemical Communications*, *55*(42), 5958-5961. DOI: 10.1039/c9cc02319d.

Chattopadhyay, P., Rai, R., and Pandey, P. S. (2006). Two‐Step Synthetic Route ton10‐Substituted Isoall- oxazines. *Synthetic communications*, *36*(13), 1857-1861. DOI: 10.1080/0039791 0600602552.
Das, K., Mondal, A., and Srimani, D. (2018). Selective synthesis of 2-substituted and 1, 2- disubstituted benzimidazoles directly from aromatic diamines and alcohols catalyzed by molecularly defined nonphosphine manganese (I) complex. *The Journal of organic chemistry*, *83*(16), 9553-9560. DOI: 10.1021/acs.joc.8b01316.

Kommi, D. N., Kumar, D., Bansal, R., Chebolu, R., and Chakraborti, A. K. (2012). “All-water” chemistry of tandem N-alkylation–reduction–condensation for synthesis of N-arylmethyl- 2- substituted benzimidazoles. *Green Chemistry*, *14*(12), 3329-3335. DOI: 10.1039/c2gc36377a.

Lin, J. P., Zhang, F. H., and Long, Y. Q. (2014). Solvent/oxidant-switchable synthesis of multisubstituted quinazolines and benzimidazoles via metal-free selective oxidative annulation of arylamidines. *Organic letters*, *16*(11), 2822-2825. DOI: org/10.1021/ol500864r.

Sun, W., Zhang, M., Li, P., and Li, Y. (2019). One-Pot Synthesis of Polysubstituted Imidazoles Based on Pd(OAc)_2_/Ce(SO_4_)_2_/Bi(NO_3_)_3_ Trimetallic Cascade of Decarboxylation Wacker-Type Oxidation /Debus–Radziszewski Reaction. *Synthesis*, *51*(17), 3221-3230. DOI: 10.1055/s-0037-1611835.

Xie, C., Han, X., Gong, J., Li, D., and Ma, C. (2017). One-pot strategy of copper-catalyzed synthesis of 1,2-disubstituted benzimidazoles. *Organic & biomolecular chemistry*, *15*(27), 5811-5819. DOI: 10.1039/c7ob00945c.

**VII. Crystallographic details of 1-(4-Iodobenzyl)-2-phenyl-1*H*-benzo[*d*]imidazole (3i)**

The single crystal of **3i** was obtained by slow evaporation of DCM. Then, its iodine position on *N*1-substituted benzyl group was unambiguously confirmed by X-ray analysis. Crystal data for **3i** (CCDC no.1997949) is as below:

| Bond precision: | C-C = 0.0054 A | Wavelength=0.71073 |
| --- | --- | --- |

| Cell: | a=9.8712(4) | b=10.0611(4) | c=10.0882(4) |
| --- | --- | --- | --- |
|  | alpha=74.767(3) | beta=61.742(4) | gamma=87.429(3) |
| Temperature: | 294 K |  |  |

|  | Calculated | Reported |
| --- | --- | --- |
| Volume | 847.76(7) | 847.76(7) |
| Space group | P -1 | P -1 |
| Hall group | -P 1 | -P 1 |
| Moiety formula | C20 H15 I N2 | C20 H15 I N2 |
| Sum formula | C20 H15 I N2 | C20 H15 I N2 |
| Mr | 410.24 | 410.24 |
| Dx,g cm-3 | 1.607 | 1.607 |
| Z | 2 | 2 |
| Mu (mm-1) | 1.889 | 1.889 |
| F000 | 404.0 | 404.0 |
| F000' | 403.17 |  |
| h,k,lmax | 12,12,12 | 12,12,12 |
| Nref | 3243 | 3244 |
| Tmin,Tmax | 0.685,0.793 | 0.975,1.000 |
| Tmin' | 0.583 |  |

| Correction method= # Reported T Limits: Tmin=0.975 Tmax=1.000 AbsCorr = MULTI-SCAN | | | | |  | |  |
| --- | --- | --- | --- | --- | --- | --- | --- |
| Data completeness= 1.000 | | Theta(max)= 25.744 | | | |  |  |
| R(reflections)= 0.0304( 2905) | | | wR2(reflections)= 0.0676( 3244) | | | | |
| S = 1.066 | Npar= 208 | | |  |  |  |  |


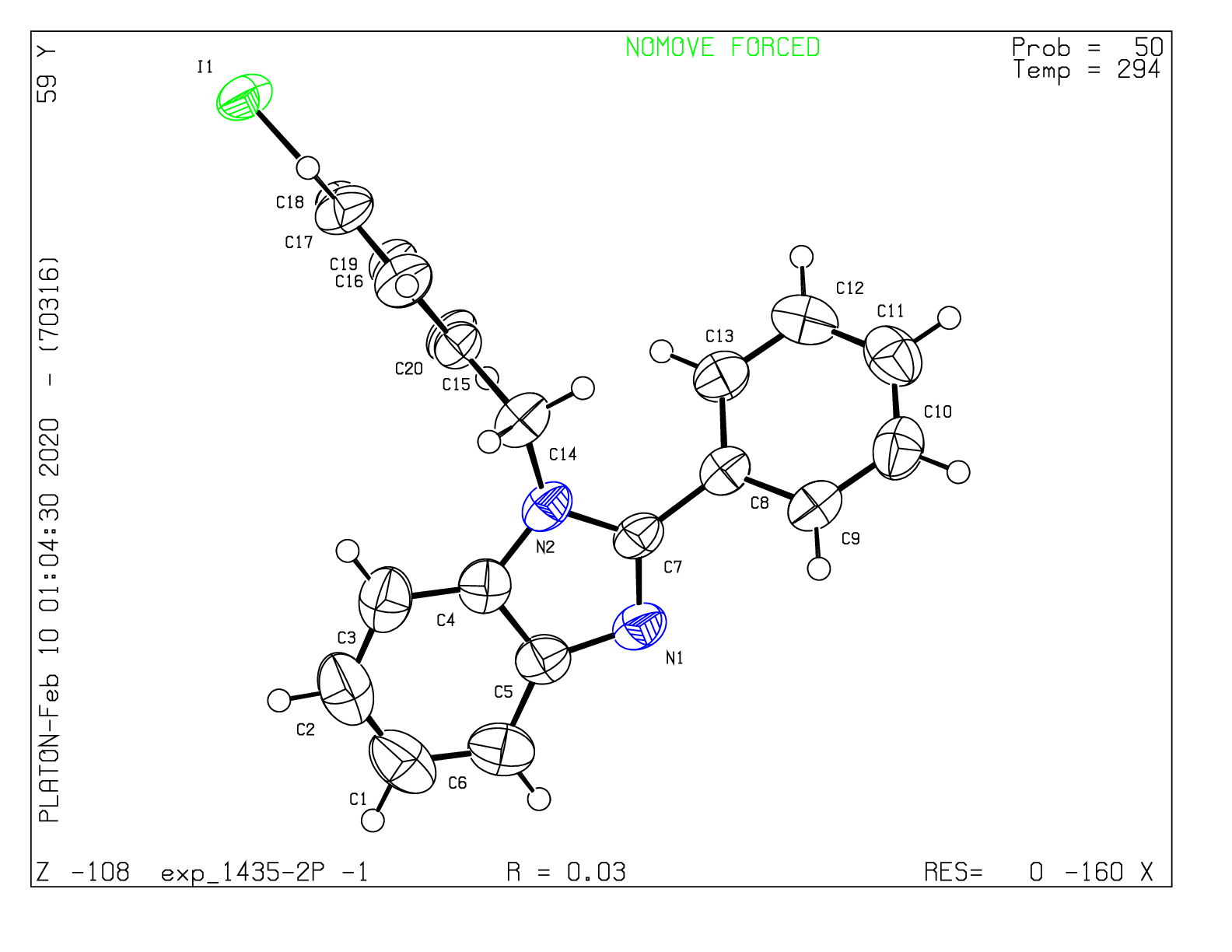


**VIII. NMR spectra**

^1^H-NMR of compound **3a** (400 MHz)

^
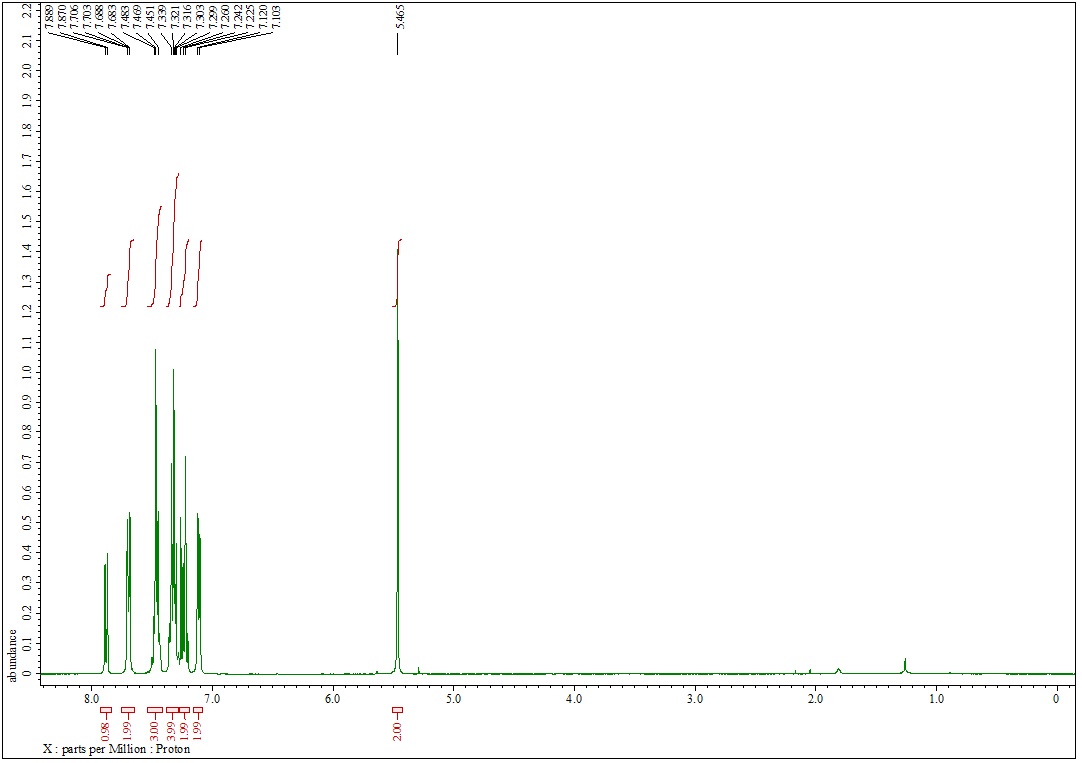
^

^13^C-NMR of compound **3a** (100 MHz)


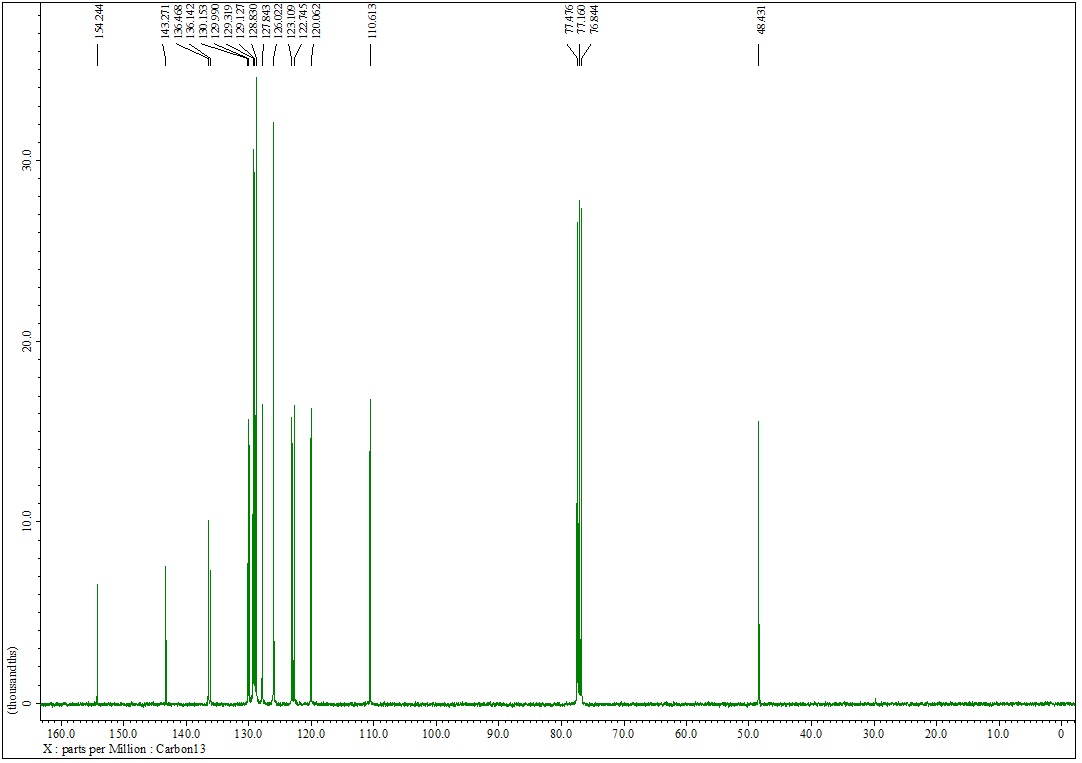


^1^H-NMR of compound **3b** (400 MHz)


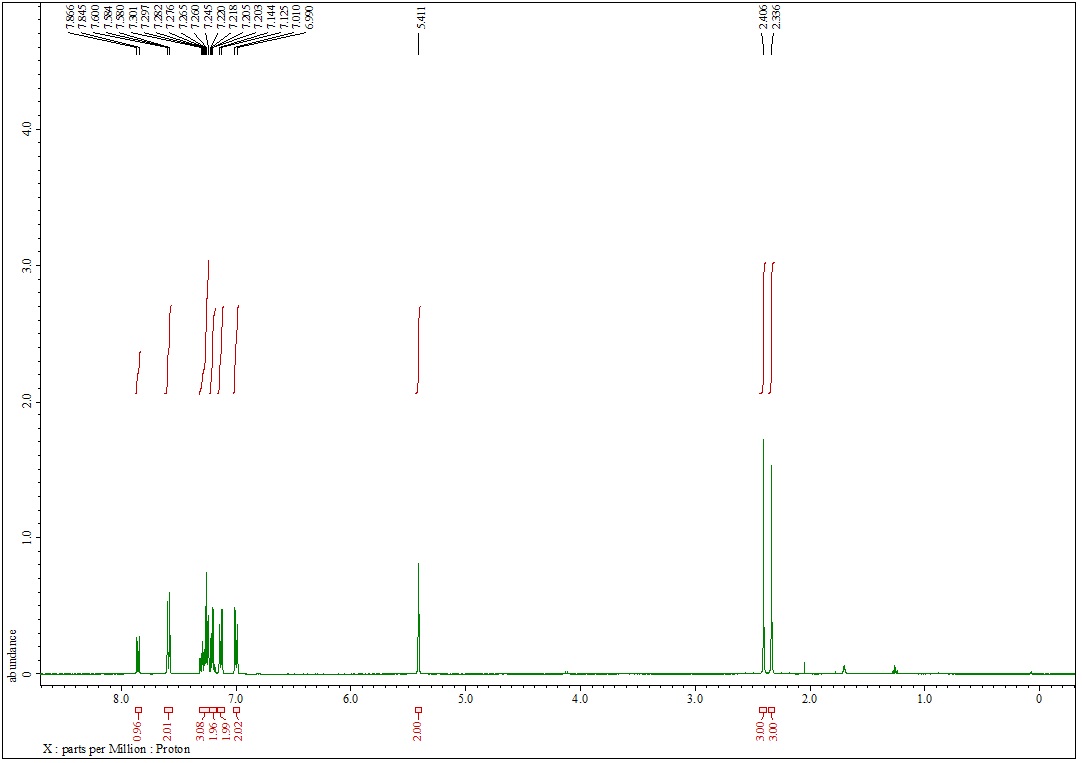


^13^C-NMR of compound **3b** (100 MHz)


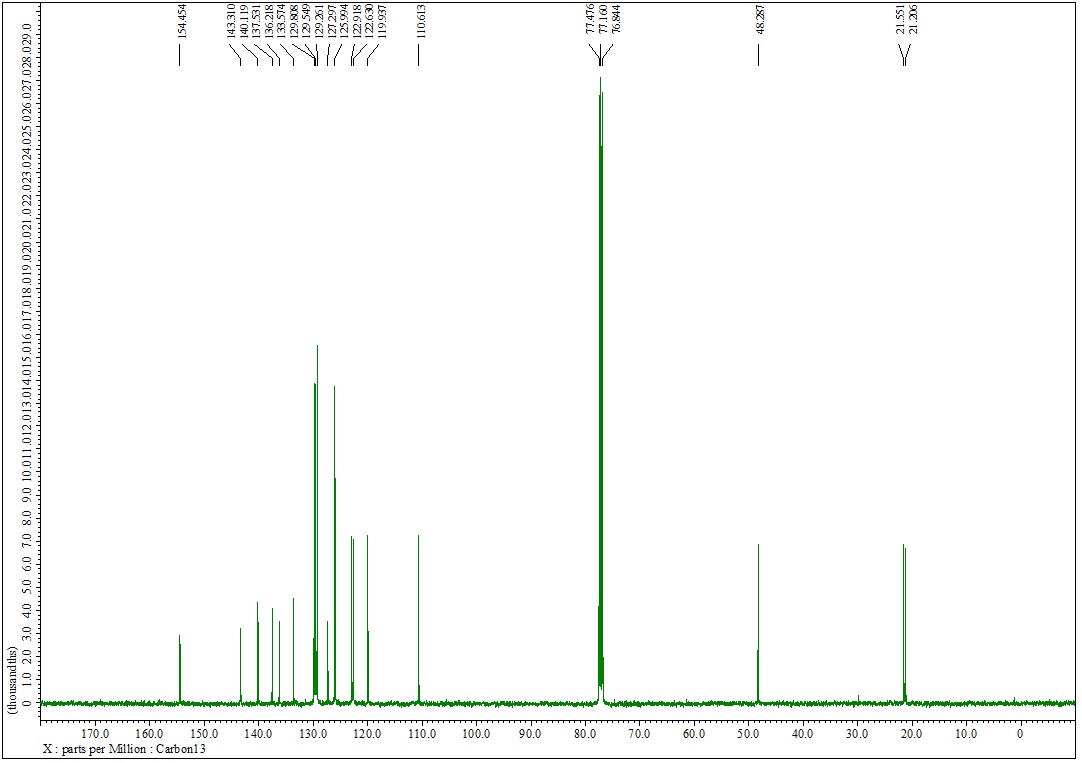


^1^H-NMR of compound **3c** (400 MHz)


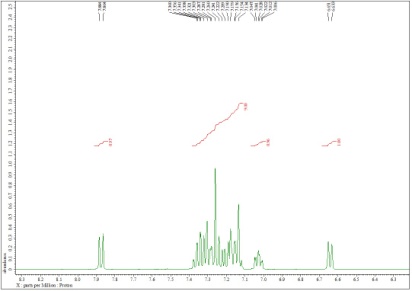

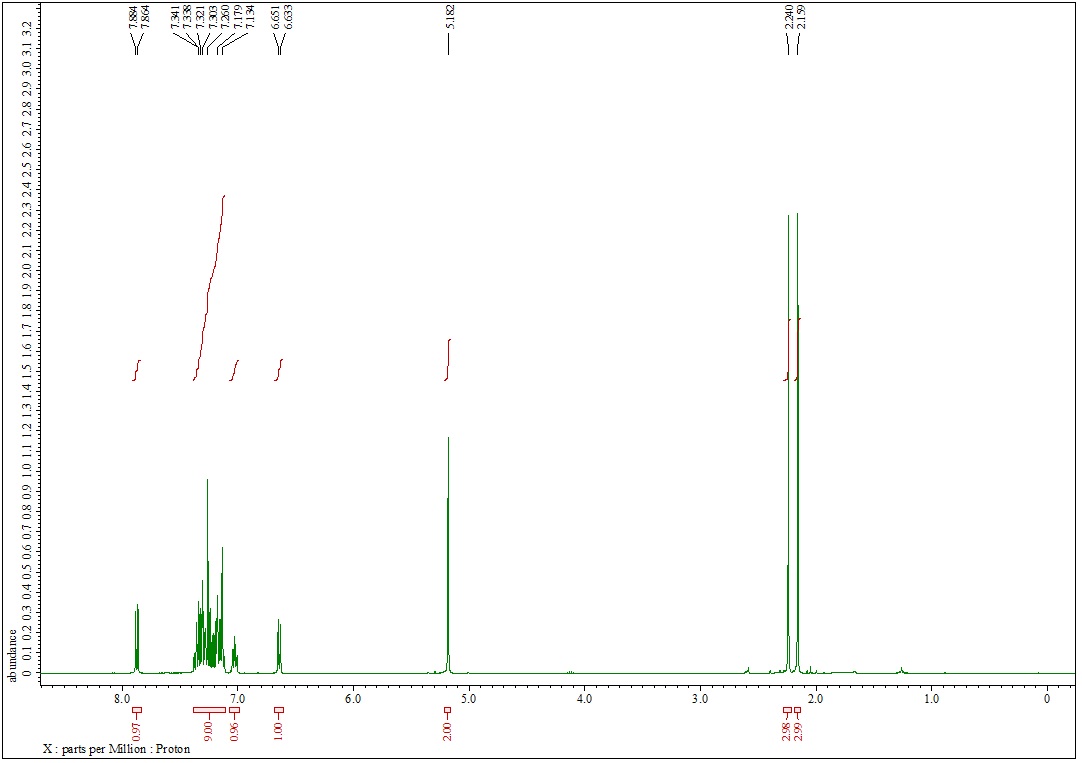


^13^C-NMR of compound **3c** (100 MHz)


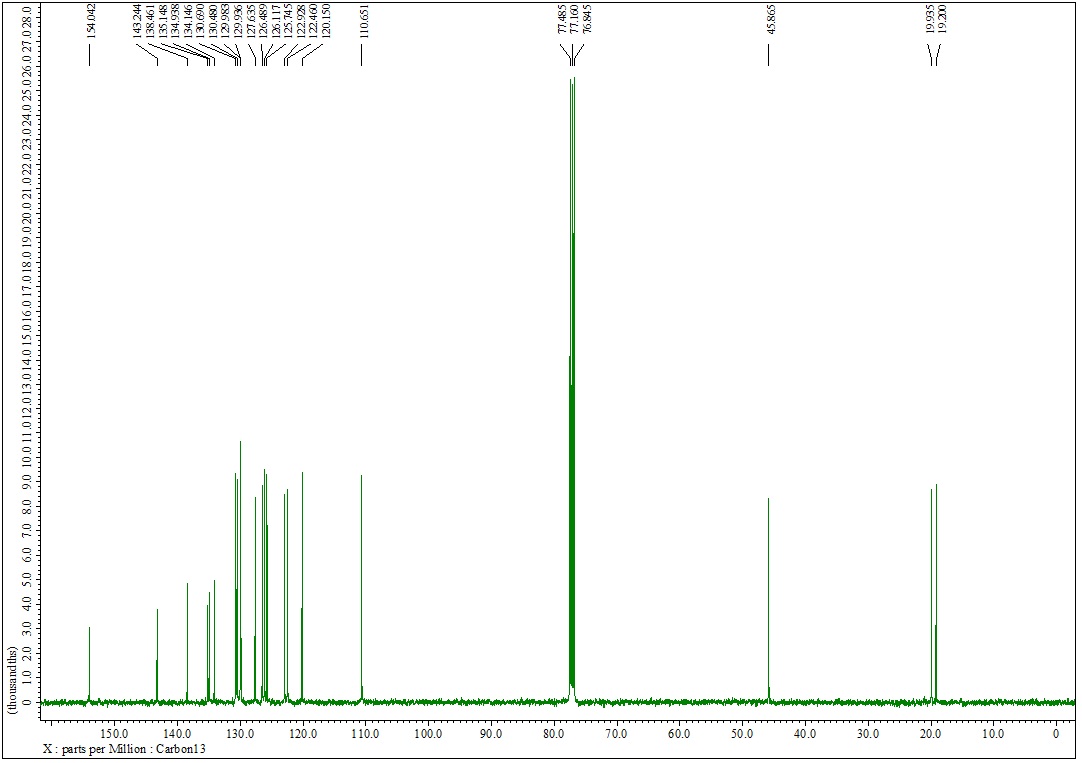


^1^H-NMR of compound **3d** (400 MHz)


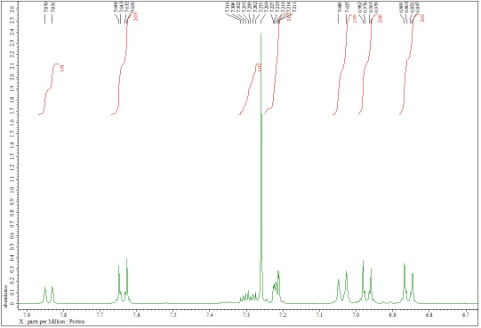

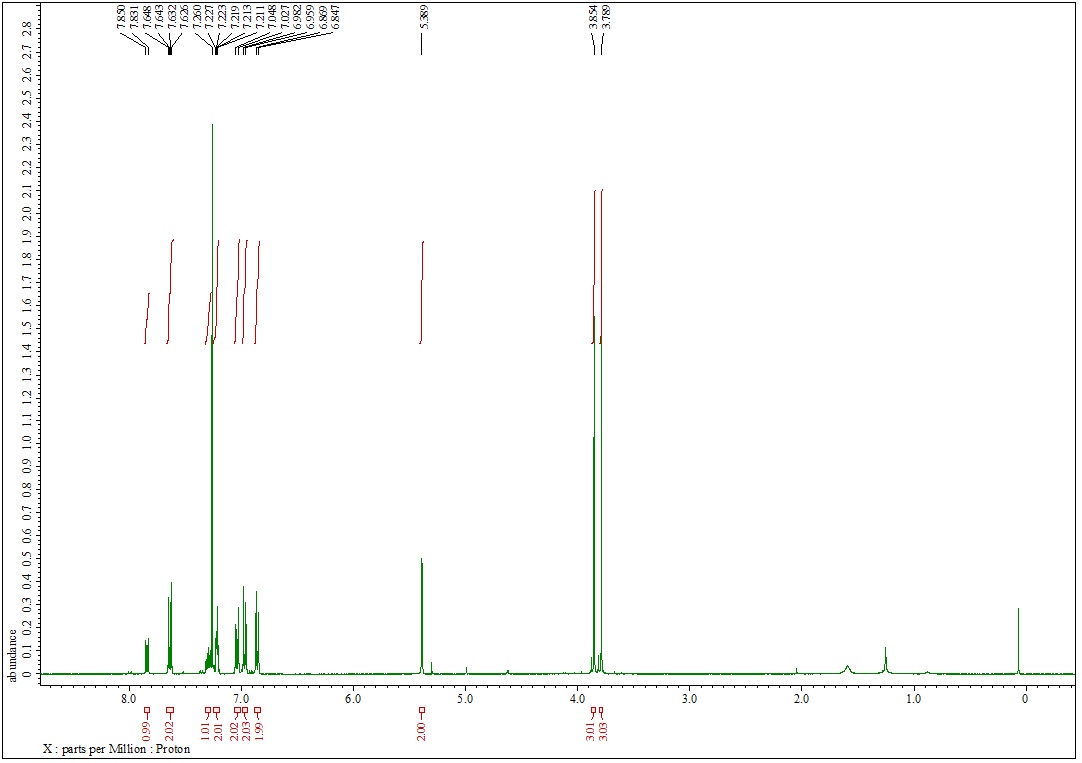


^13^C-NMR of compound **3d** (100 MHz)


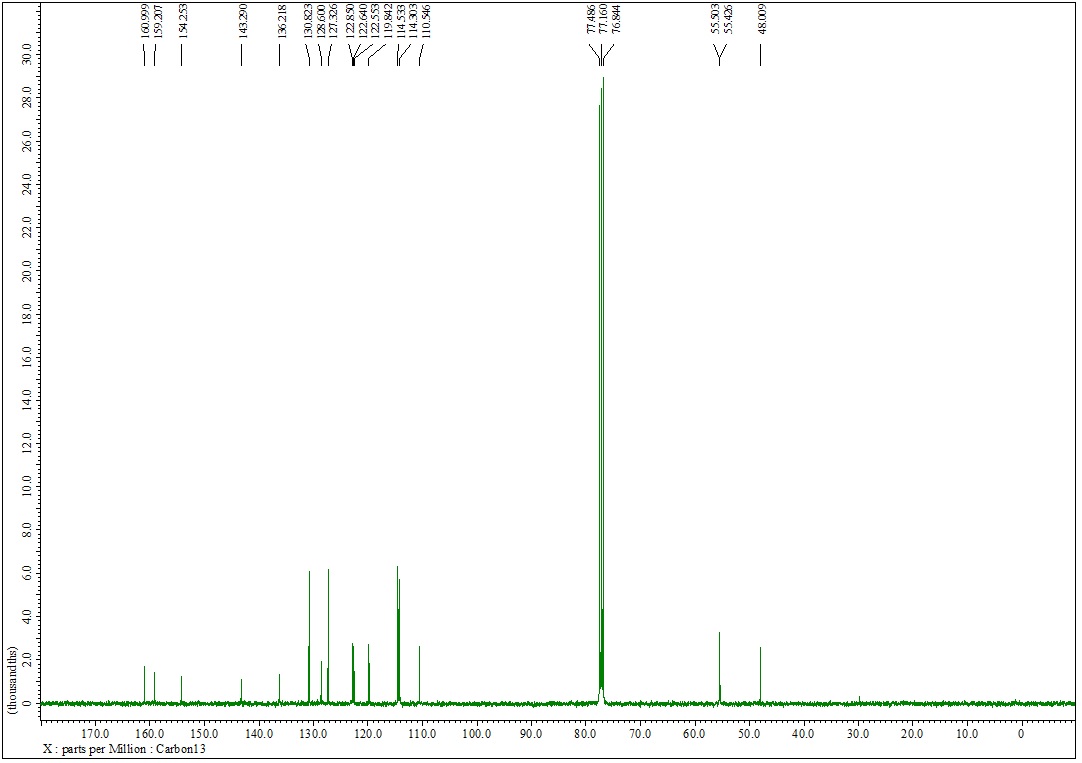


^1^H-NMR of compound **3e** (400 MHz)


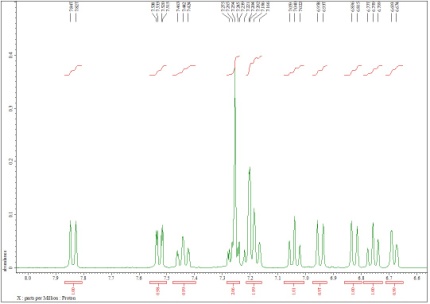

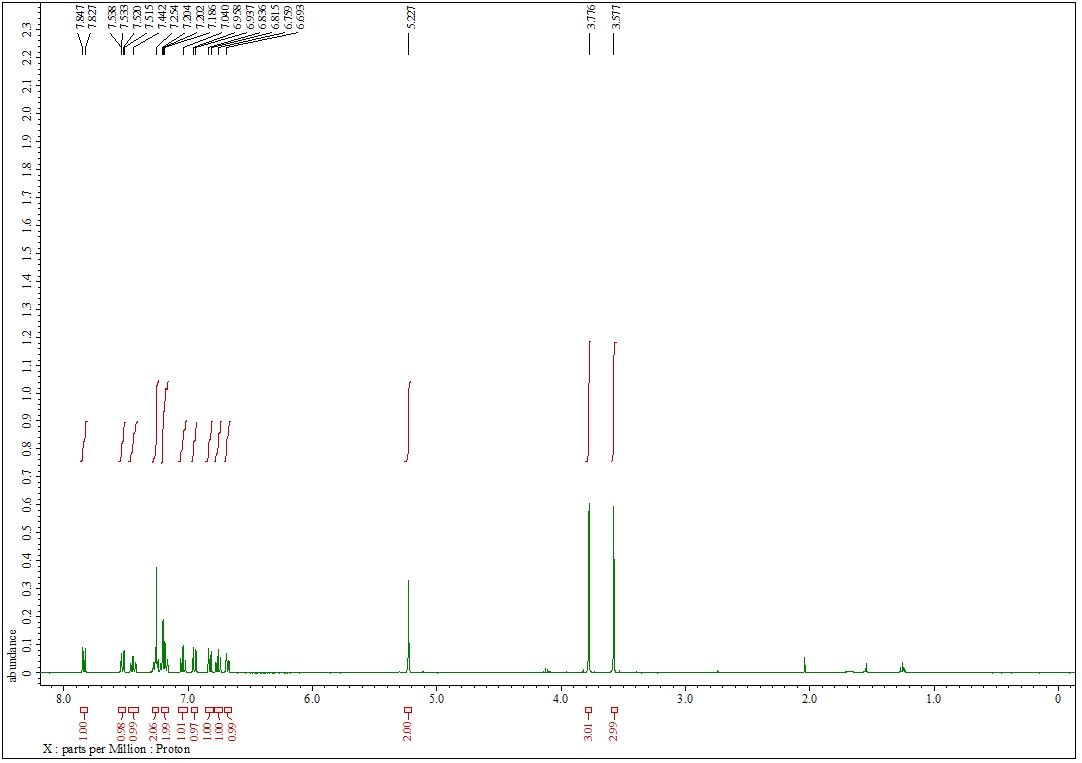


^13^C-NMR of compound **3e** (100 MHz)


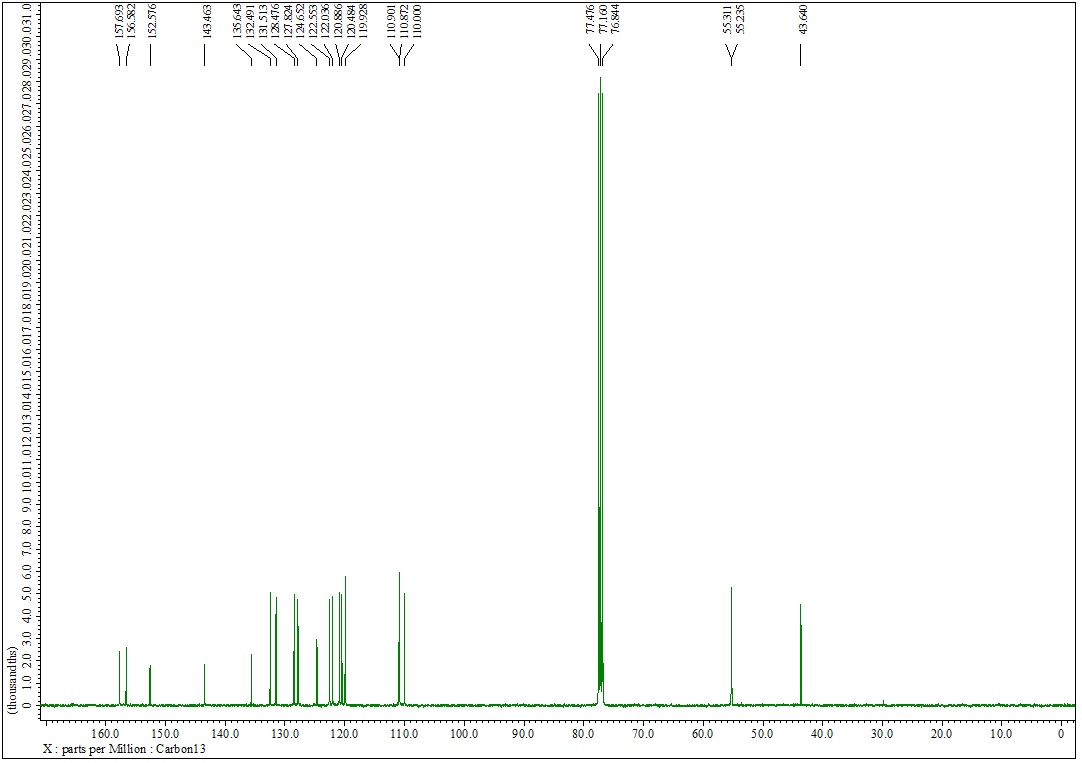


^1^H-NMR of compound **3f** (400 MHz)


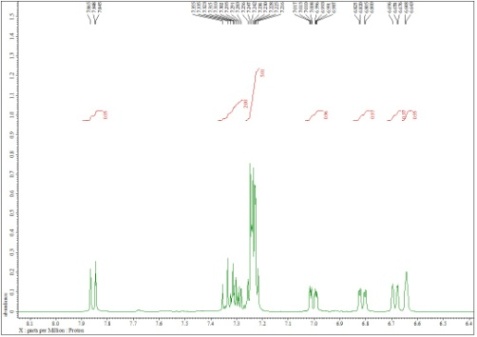

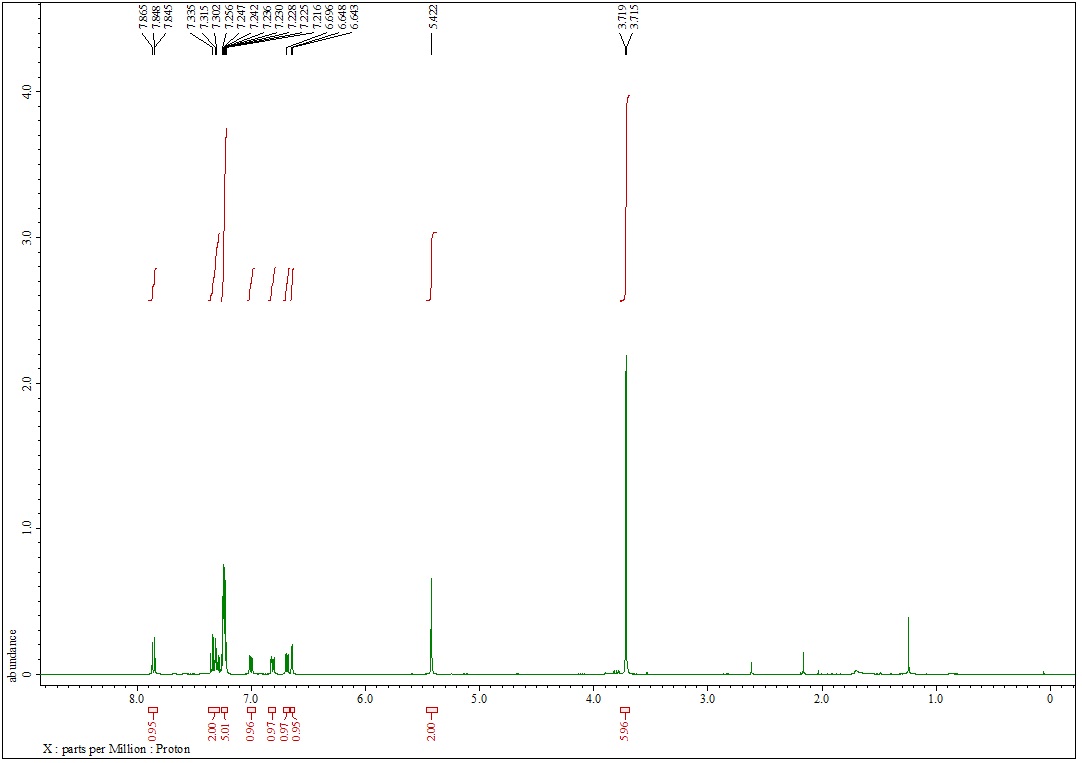


^13^C-NMR of compound **3f** (100 MHz)


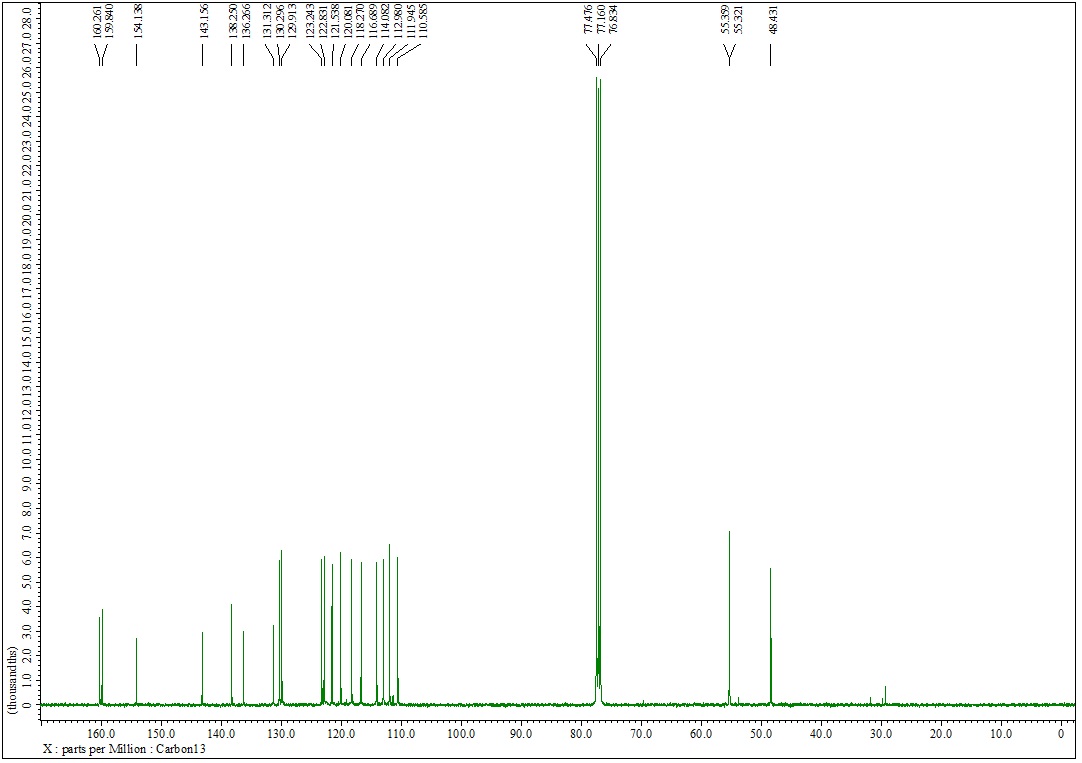


^1^H-NMR of compound **3g** (400 MHz)


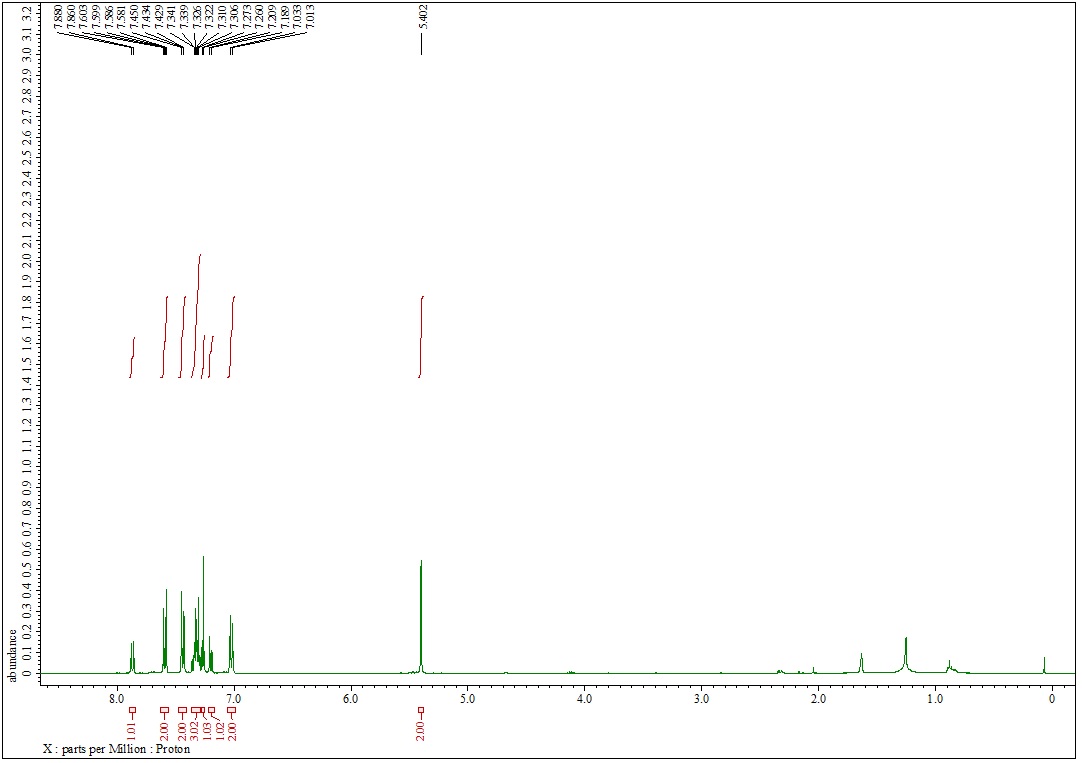


^13^C-NMR of compound **3g** (100 MHz)


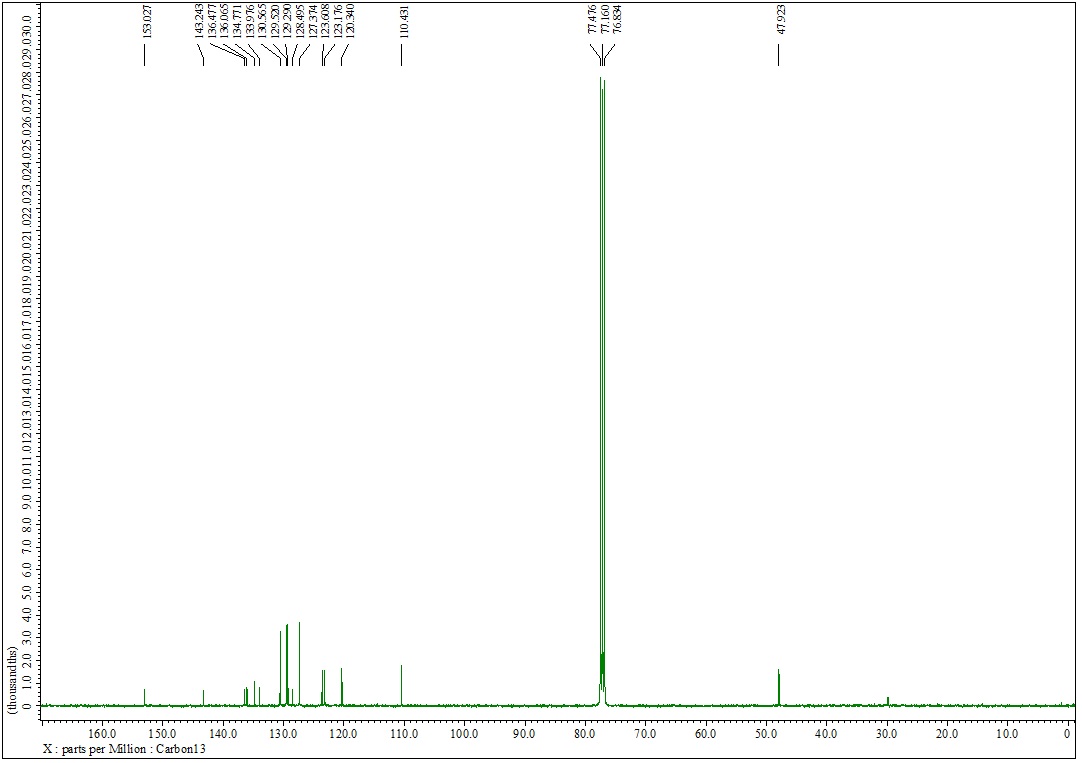


^1^H-NMR of compound **3h** (400 MHz)


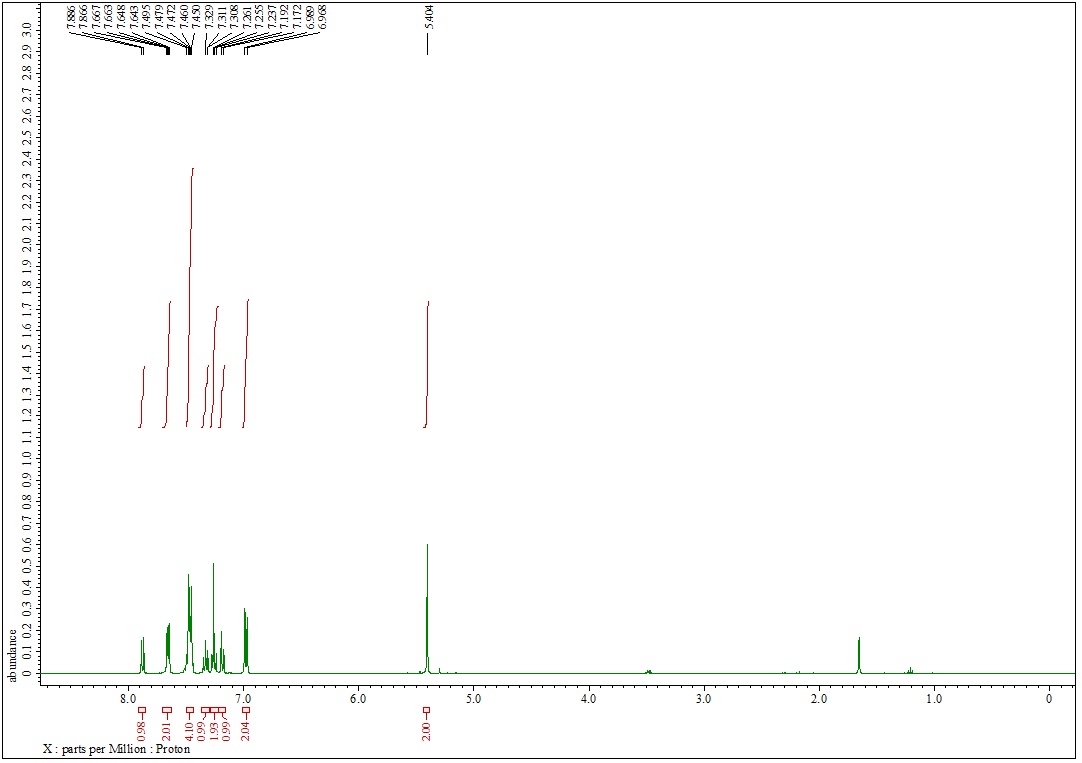


^13^C-NMR of compound **3h** (100 MHz)


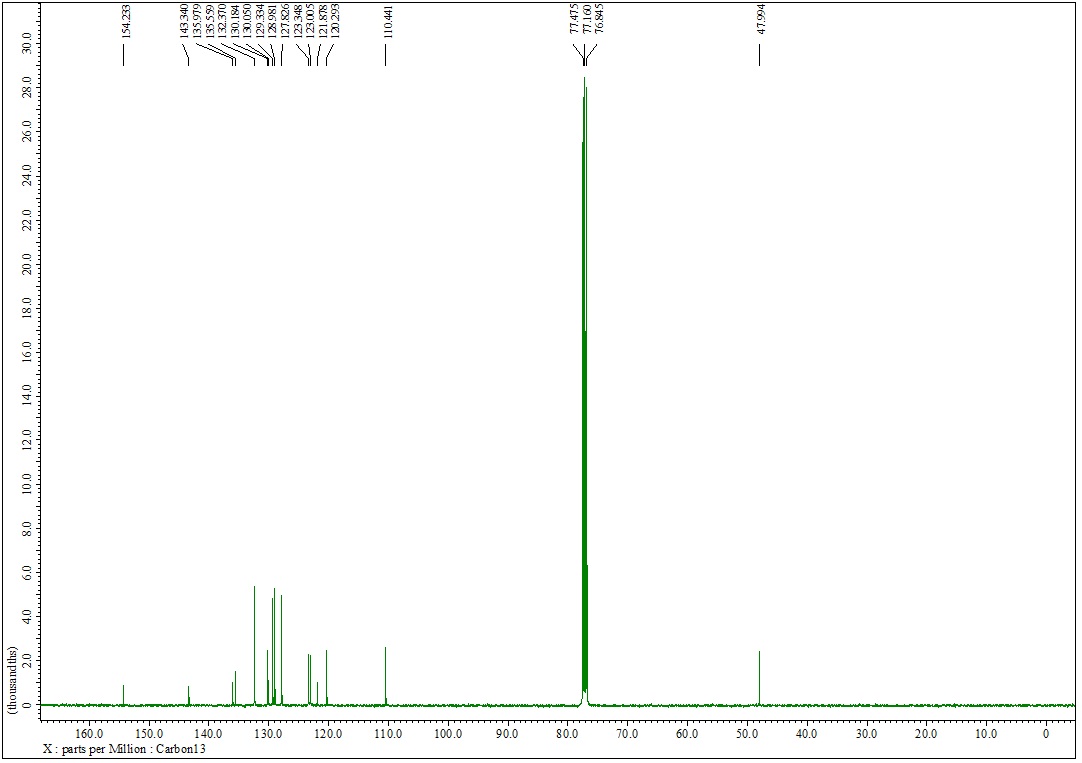


^1^H-NMR of compound **3i** (400 MHz)


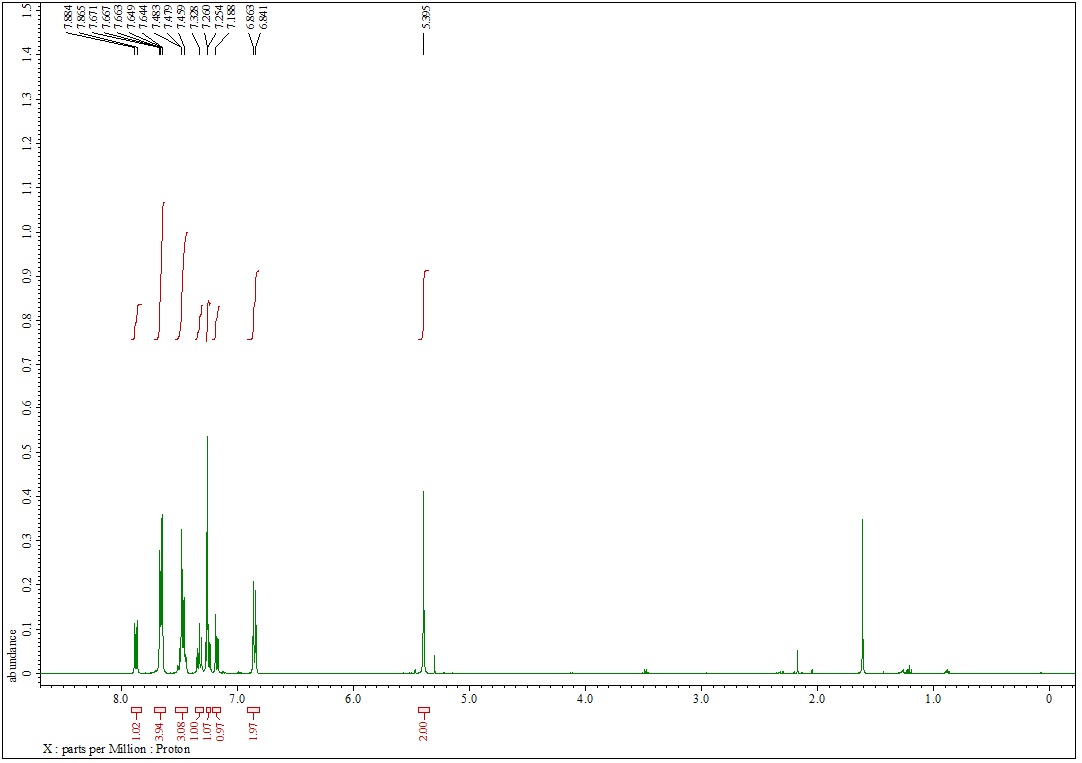


^13^C-NMR of compound **3i** (100 MHz)


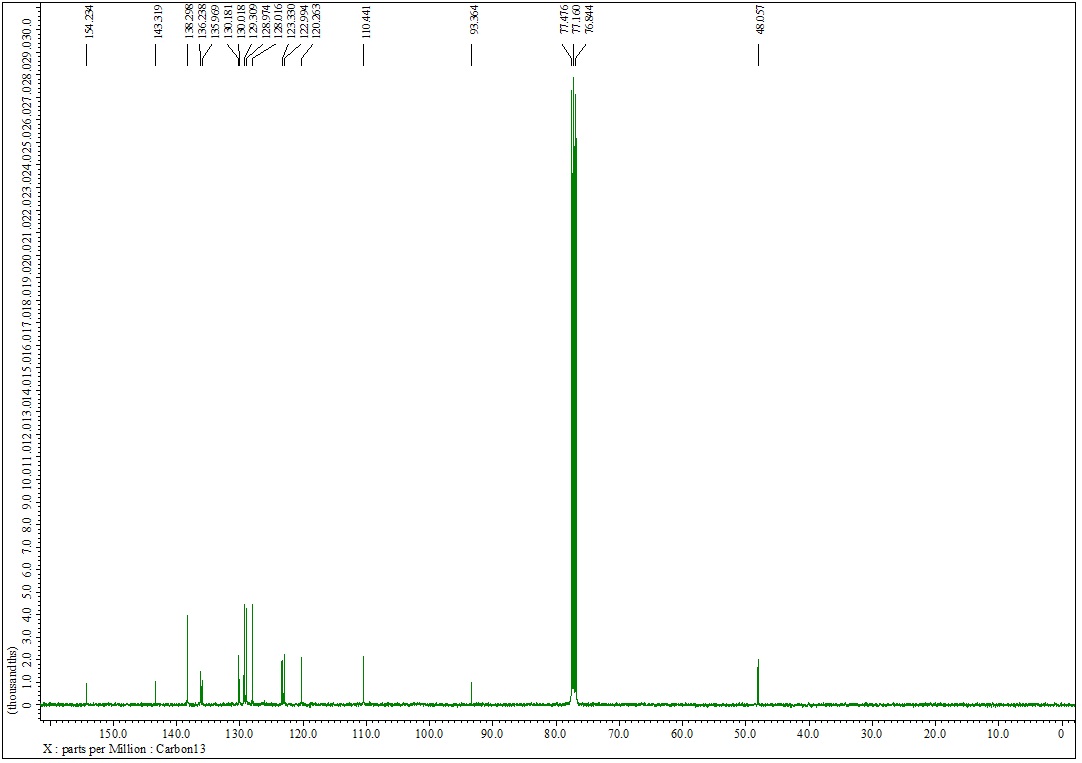


^1^H-NMR of compound **3j** (400 MHz)


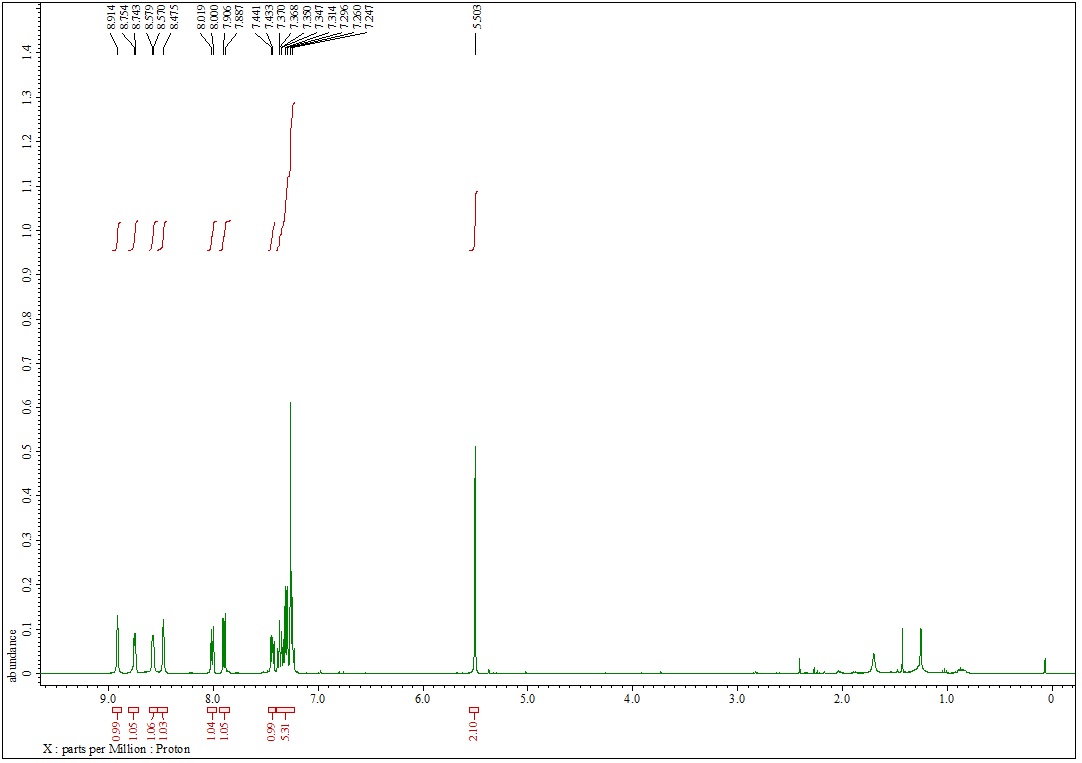


^13^C-NMR of compound **3j** (100 MHz)


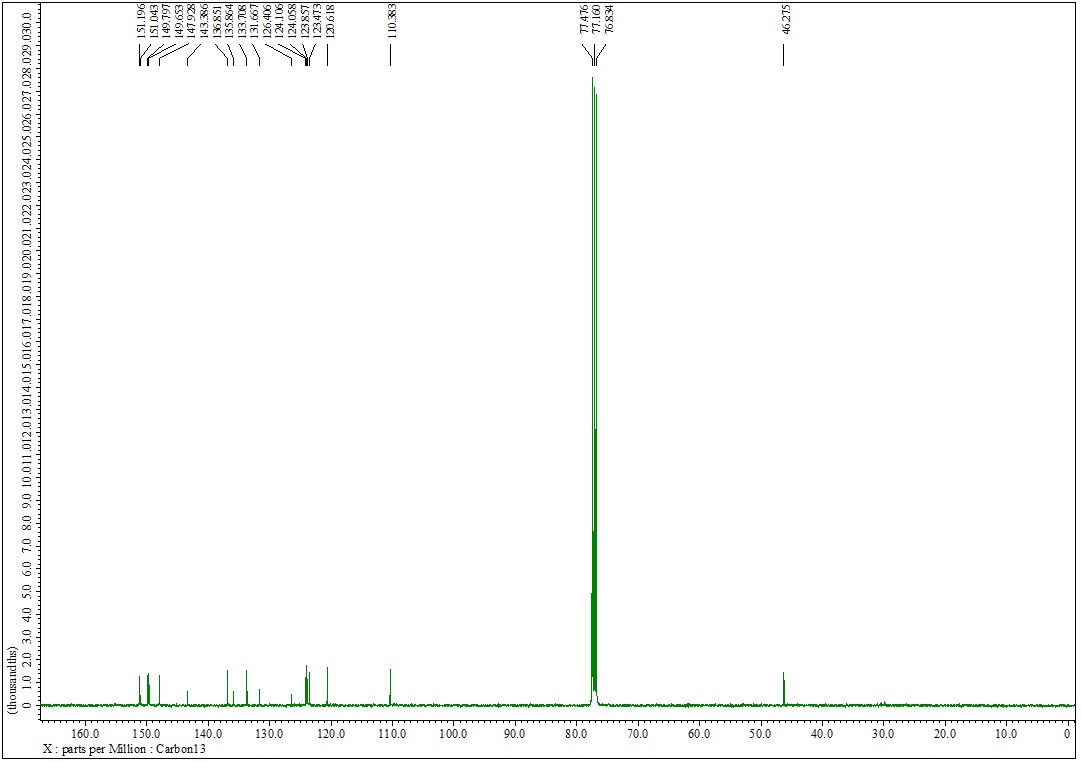


^1^H-NMR of compound **3k** (400 MHz)


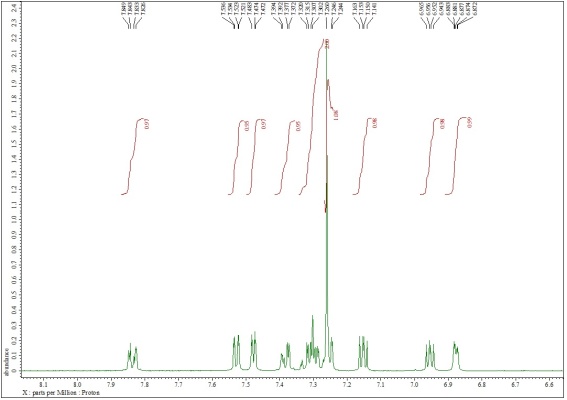

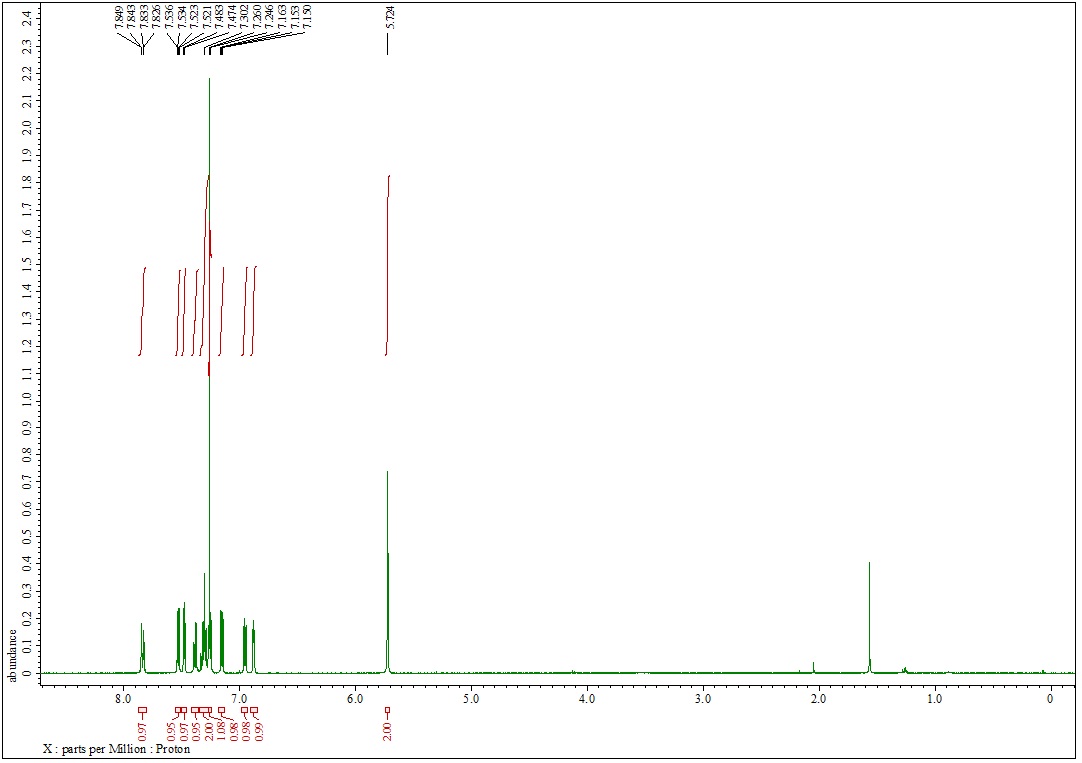


^13^C-NMR of compound **3k** (100 MHz)


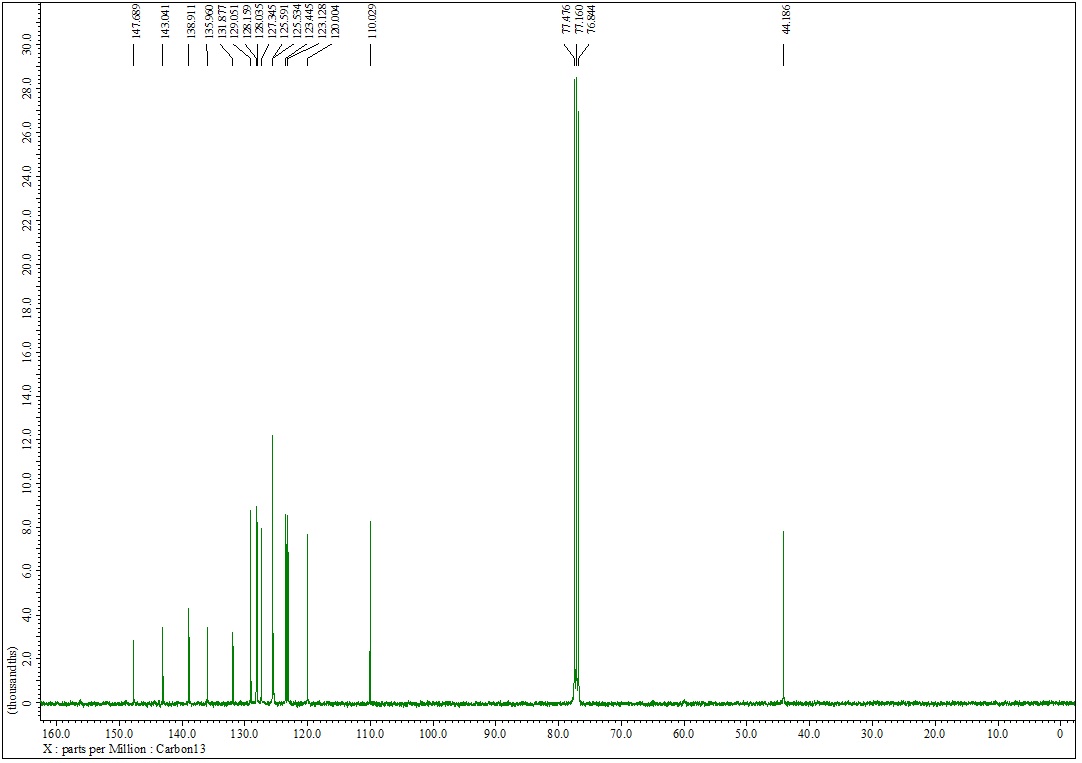


^1^H-NMR of compound **3l** (400 MHz)


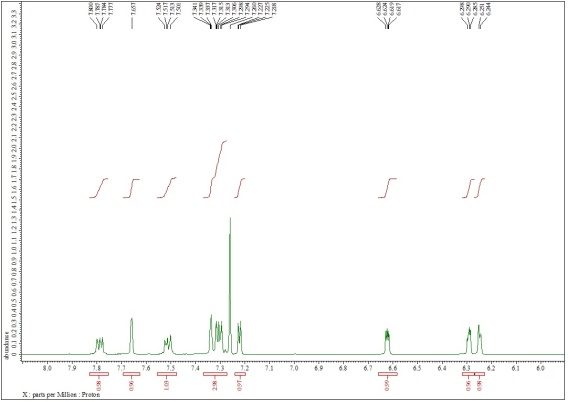

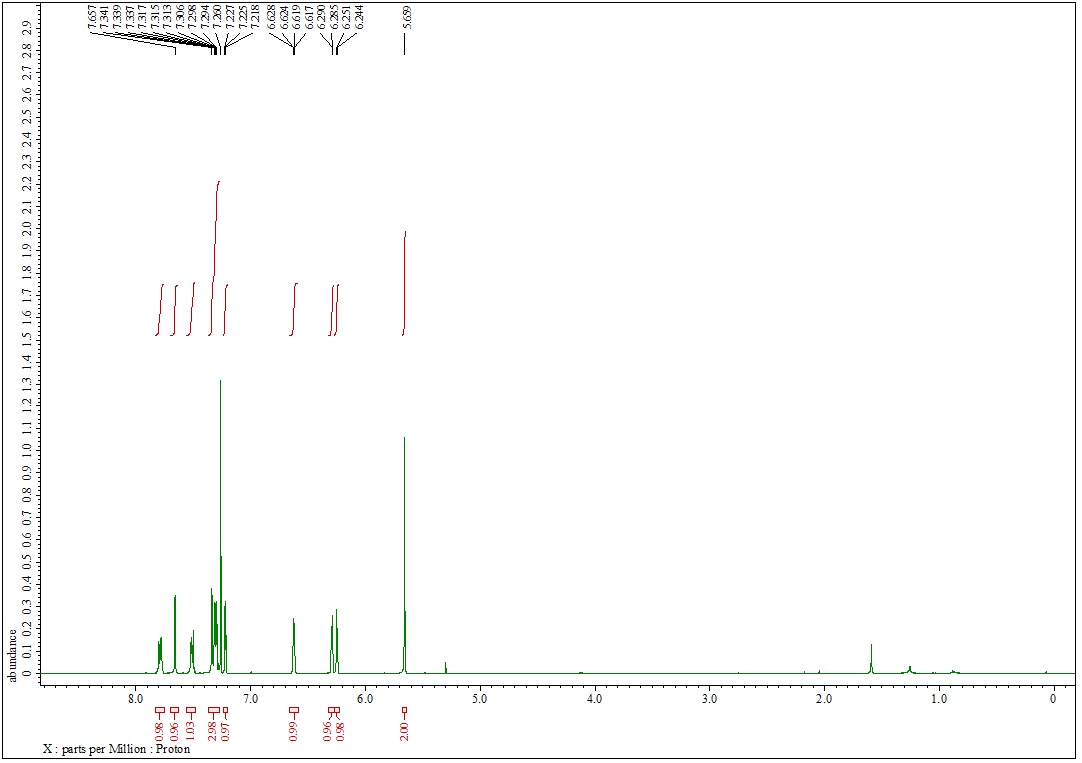


^13^C-NMR of compound **3l** (100 MHz)


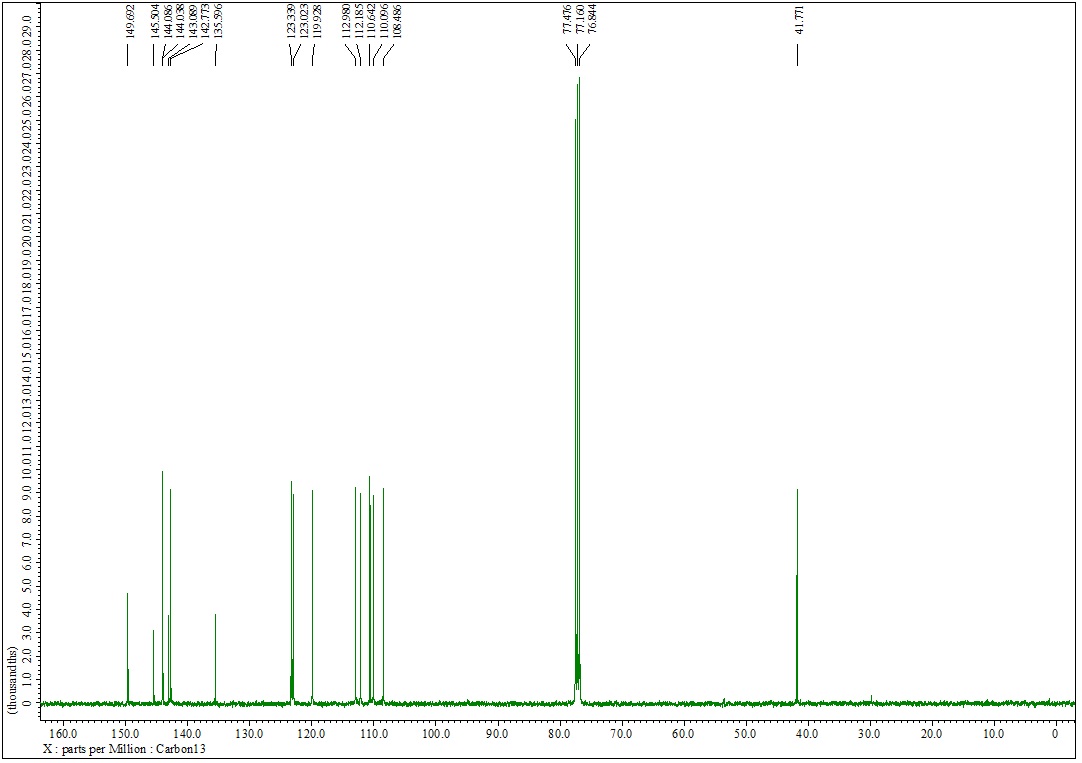


^1^H-NMR of compound **3m** (400 MHz)

**
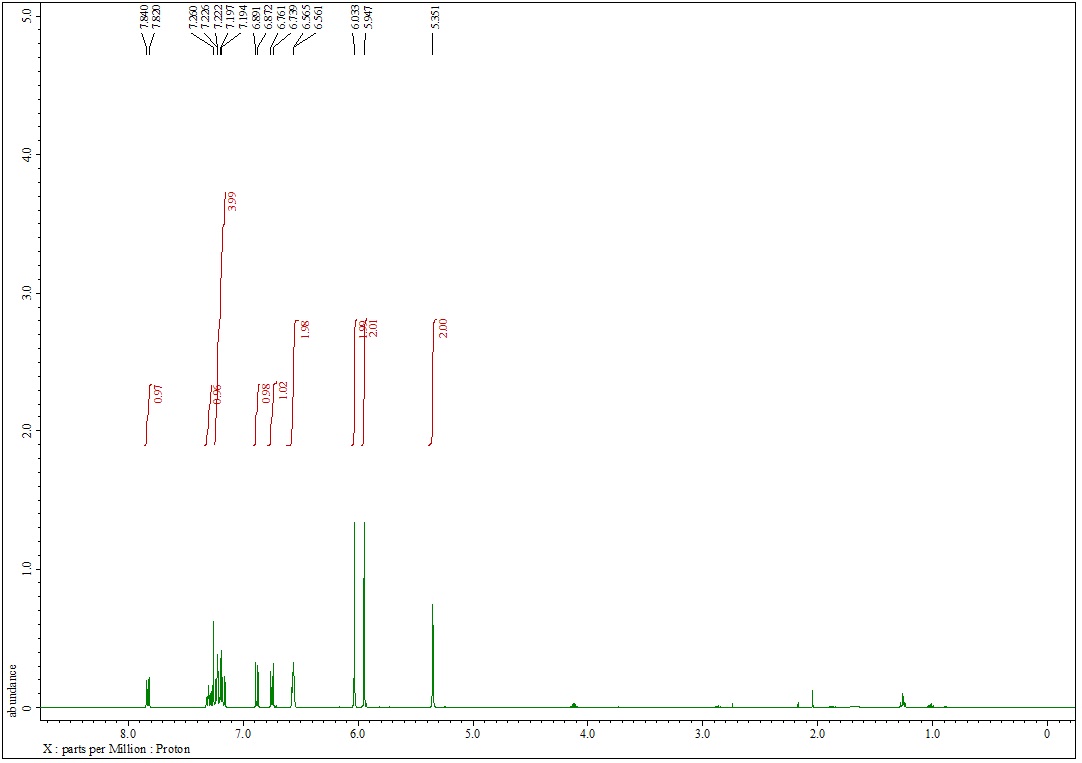
3m** (400 MHz)


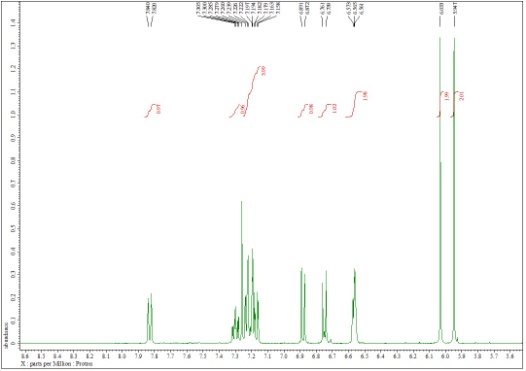


^13^C-NMR of compound **3m** (100 MHz)


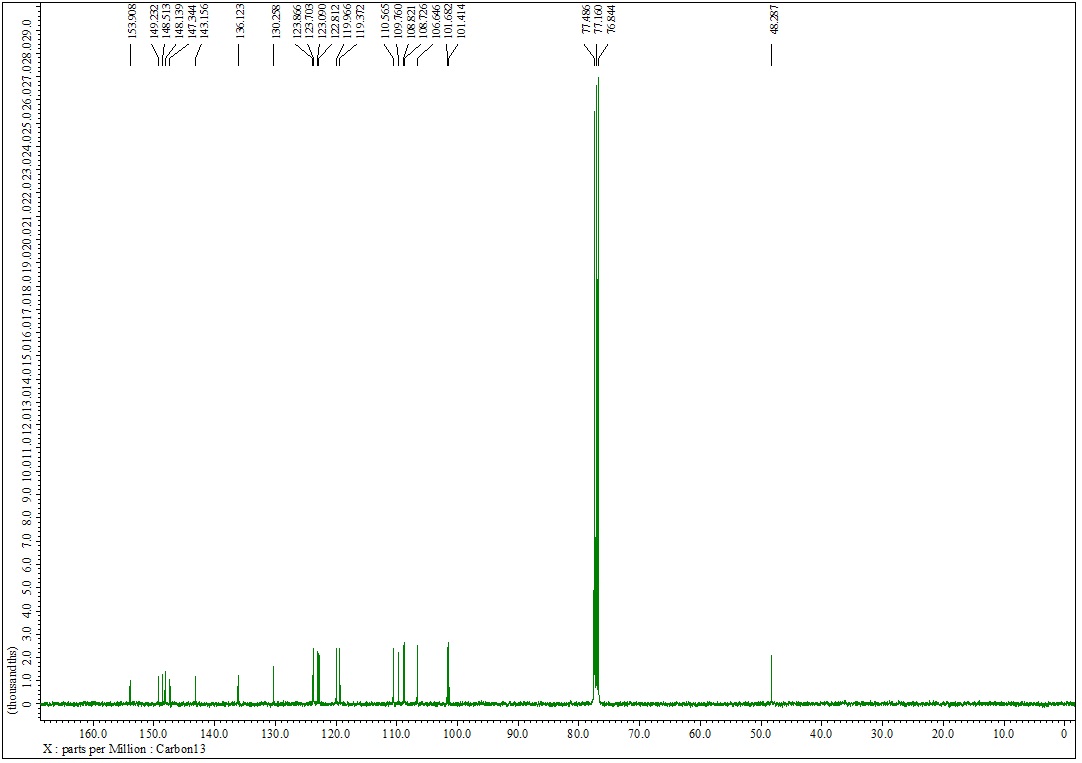


^1^H-NMR of compound **3n** (400 MHz)


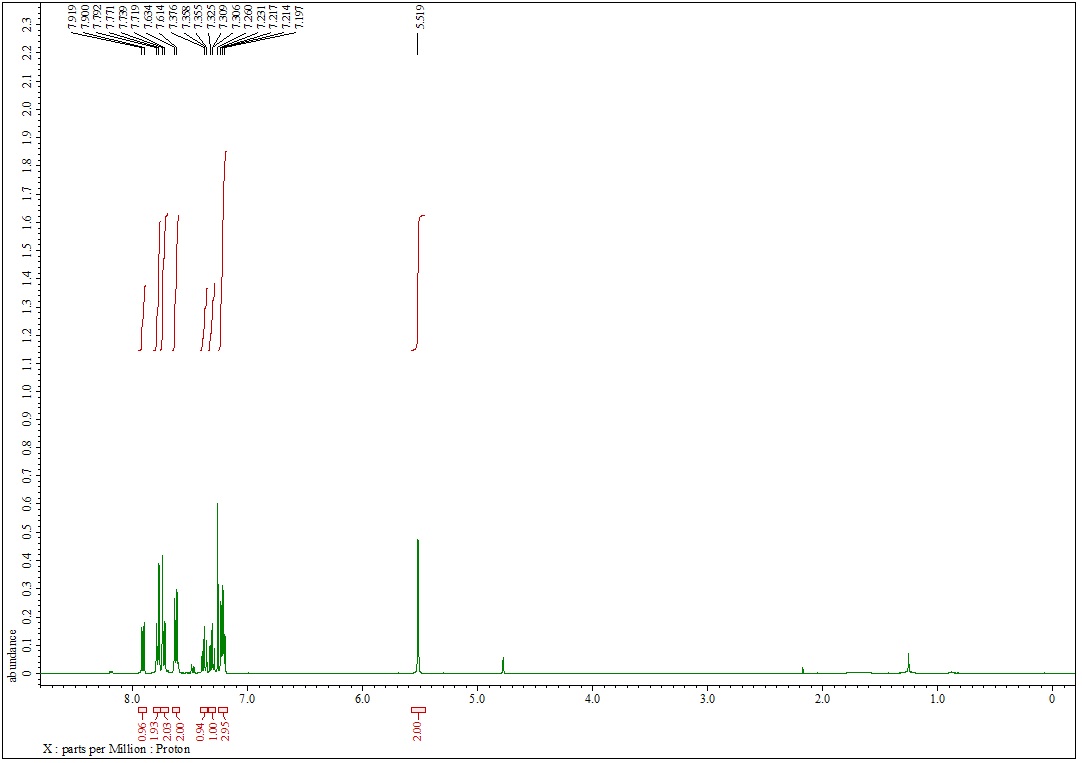


^13^C-NMR of compound **3n** (100 MHz)


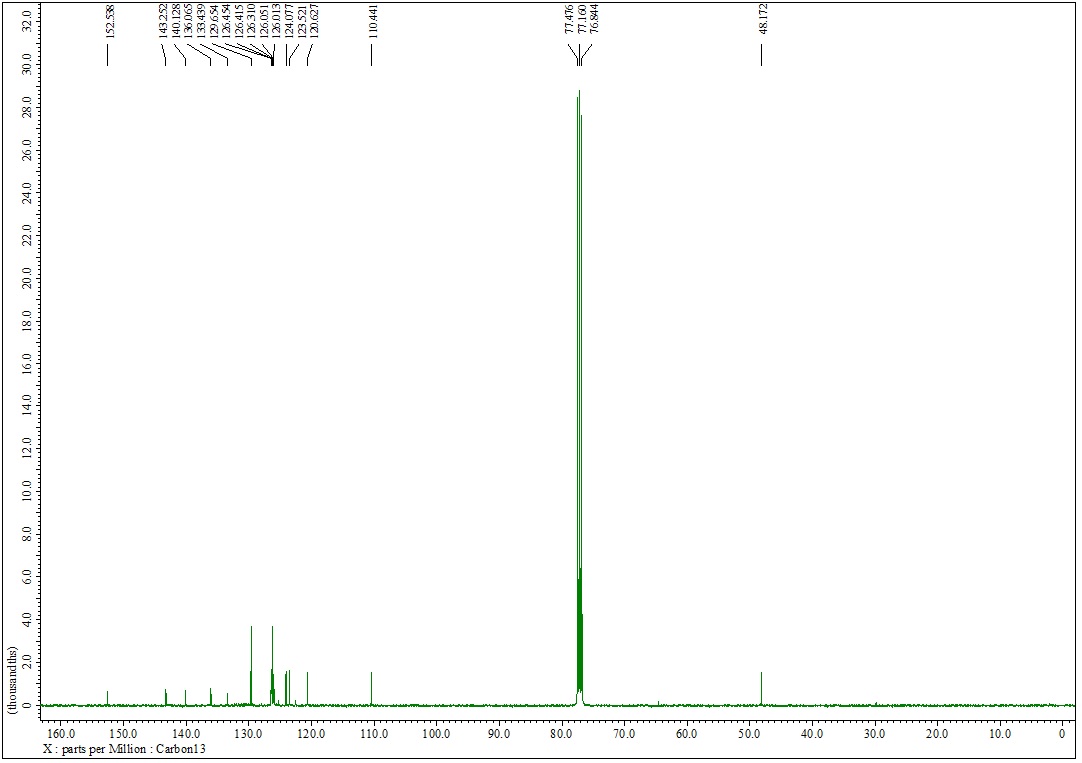


^1^H-NMR of compound **3o** (400 MHz)


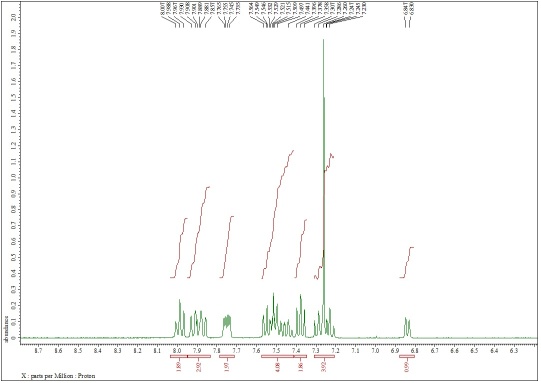

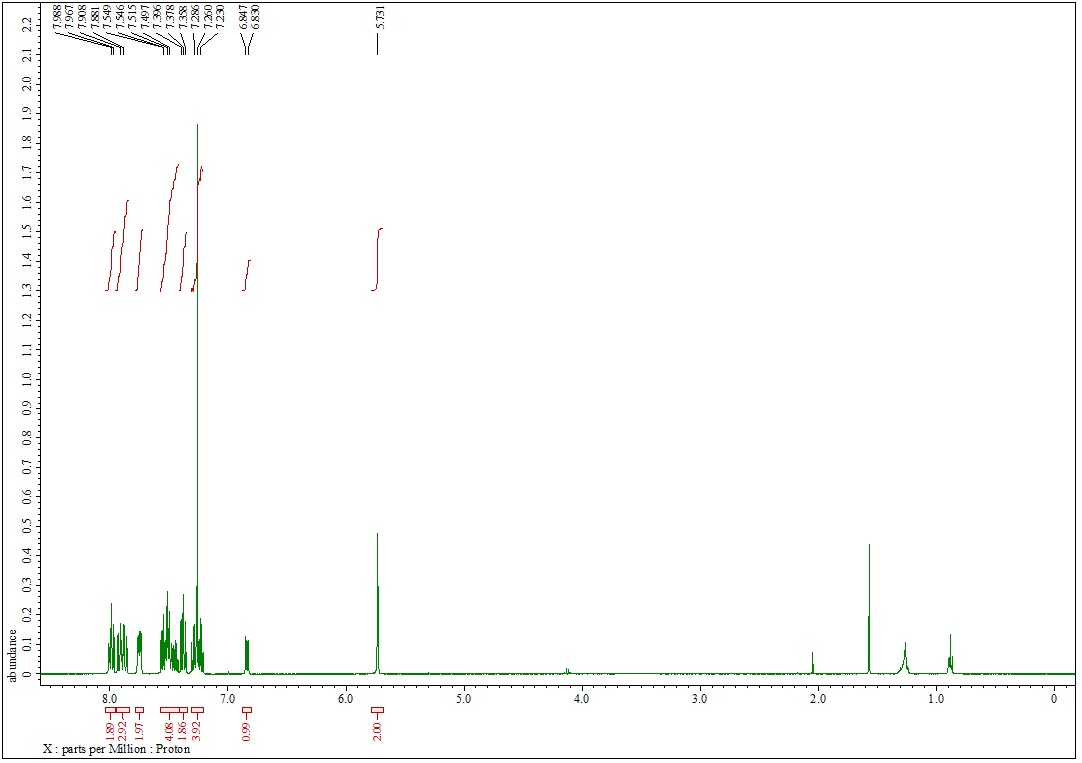


^13^C-NMR of compound **3o** (100 MHz)


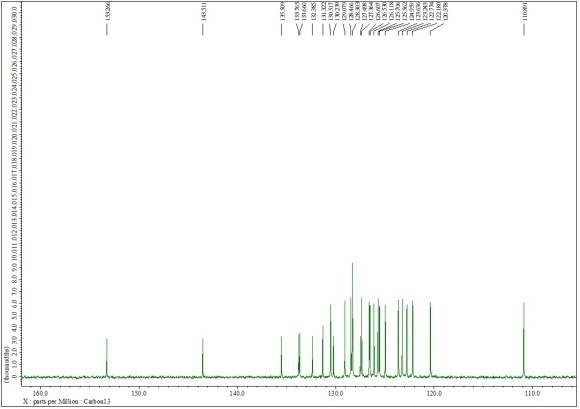

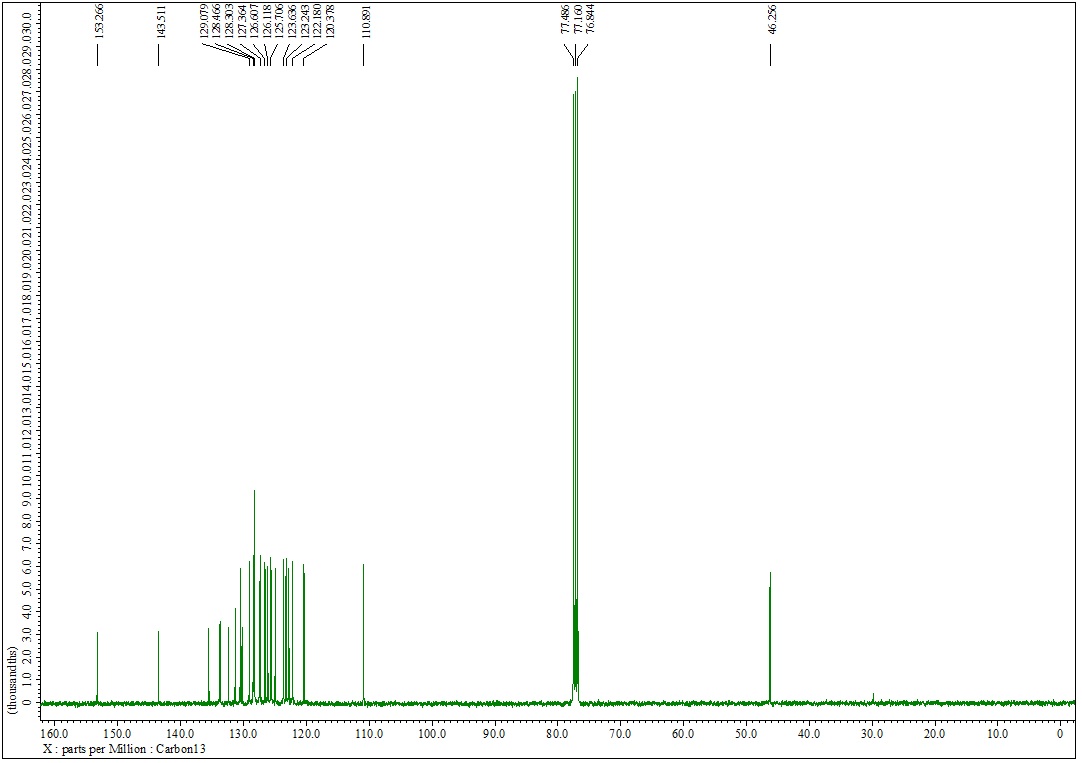


^1^H-NMR of compound **3p** (400 MHz)


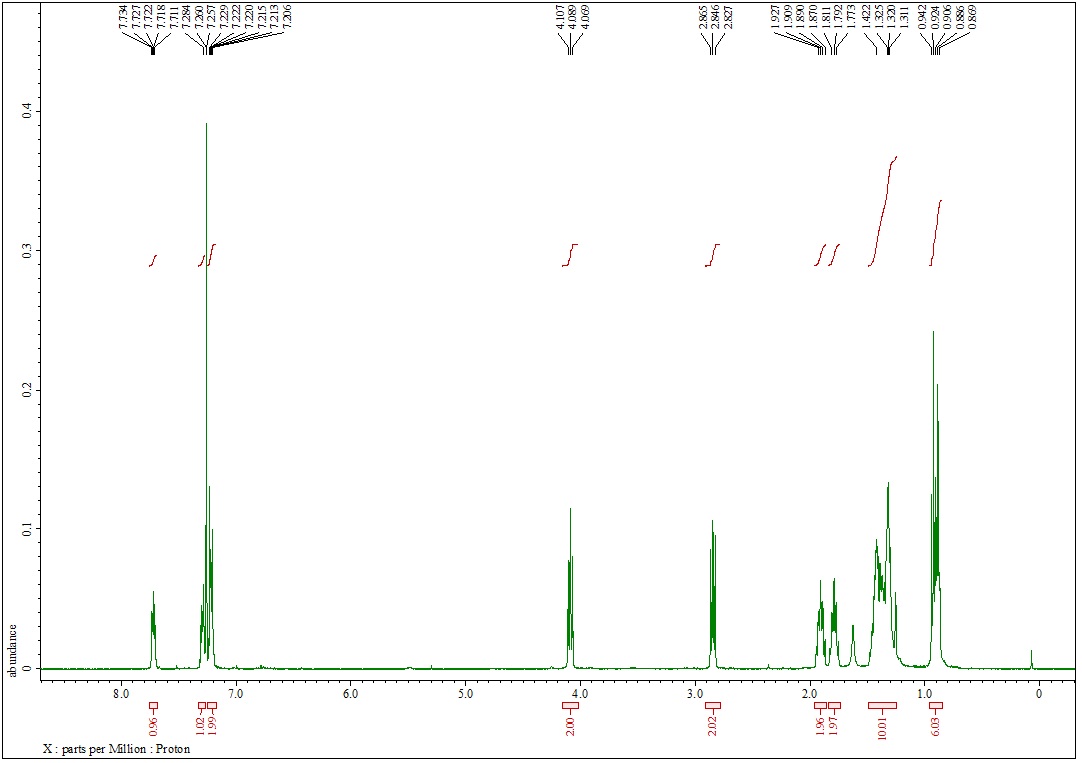


^13^C-NMR of compound **3p** (100 MHz)


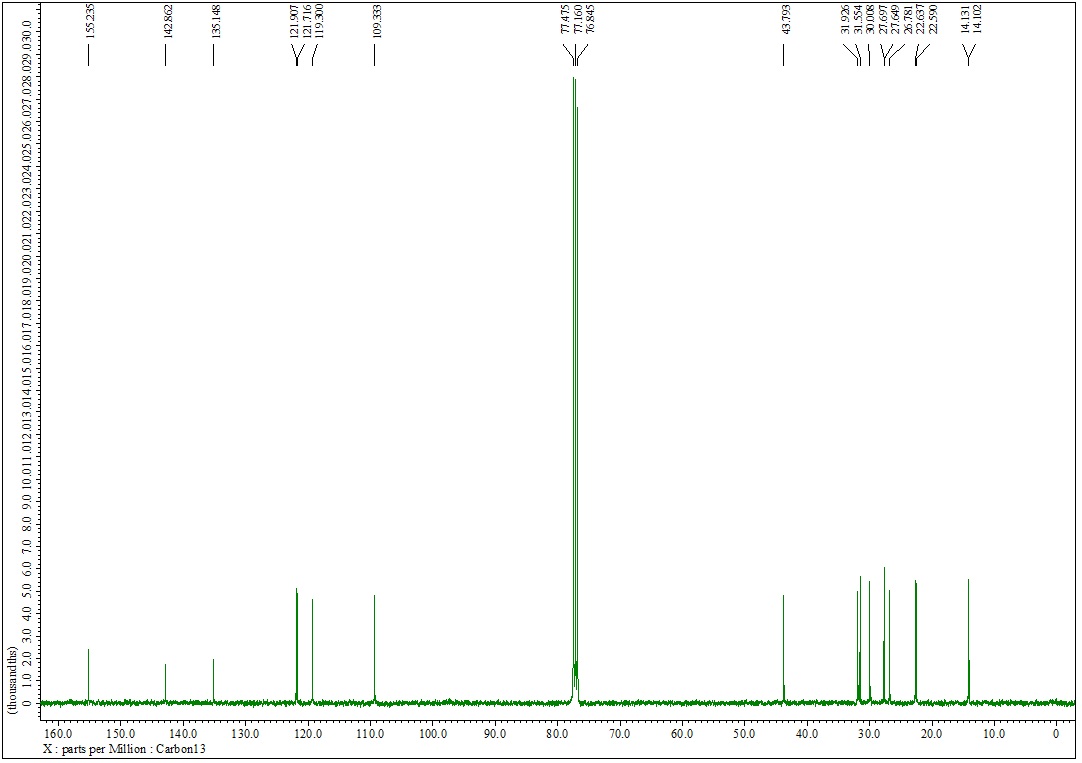


^1^H-NMR of compound **3q** (400 MHz)


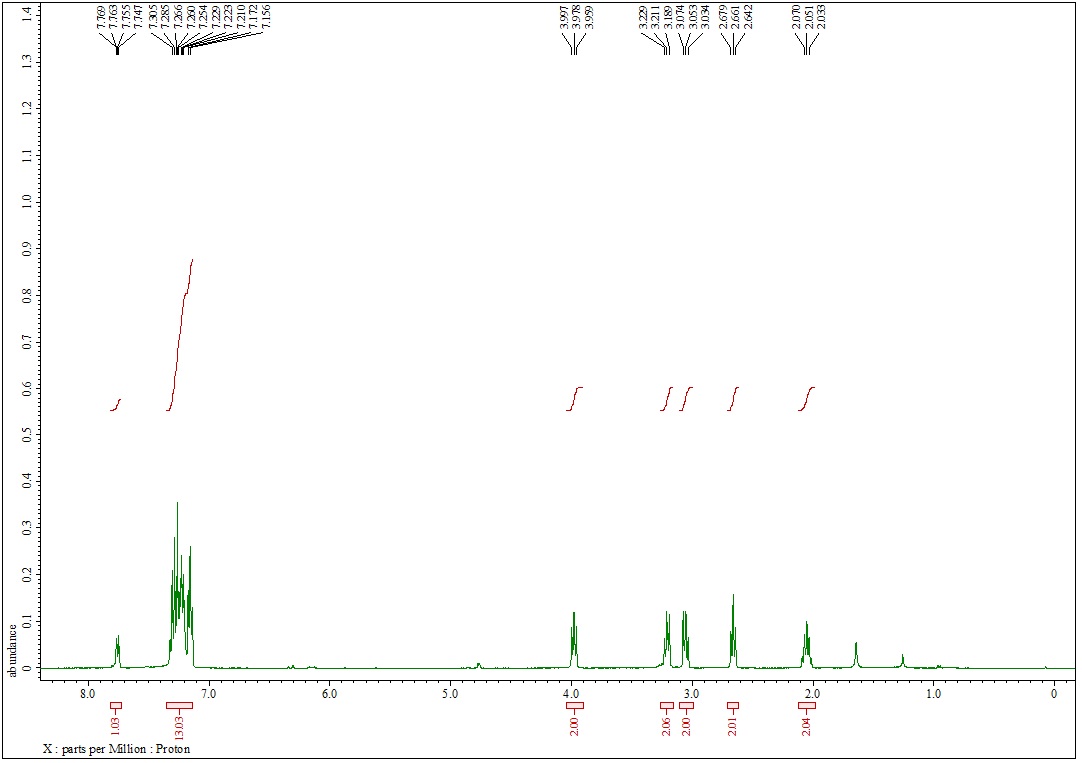


^13^C-NMR of compound **3q** (100 MHz)


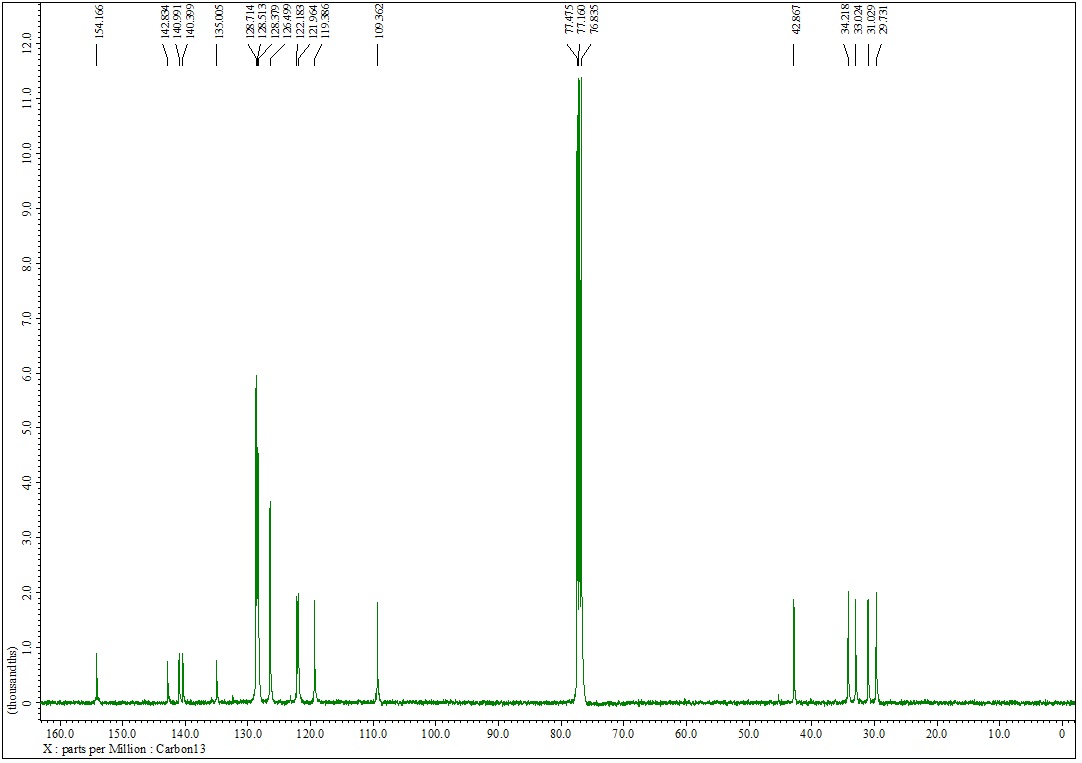


^1^H-NMR of compound **4** (400 MHz)


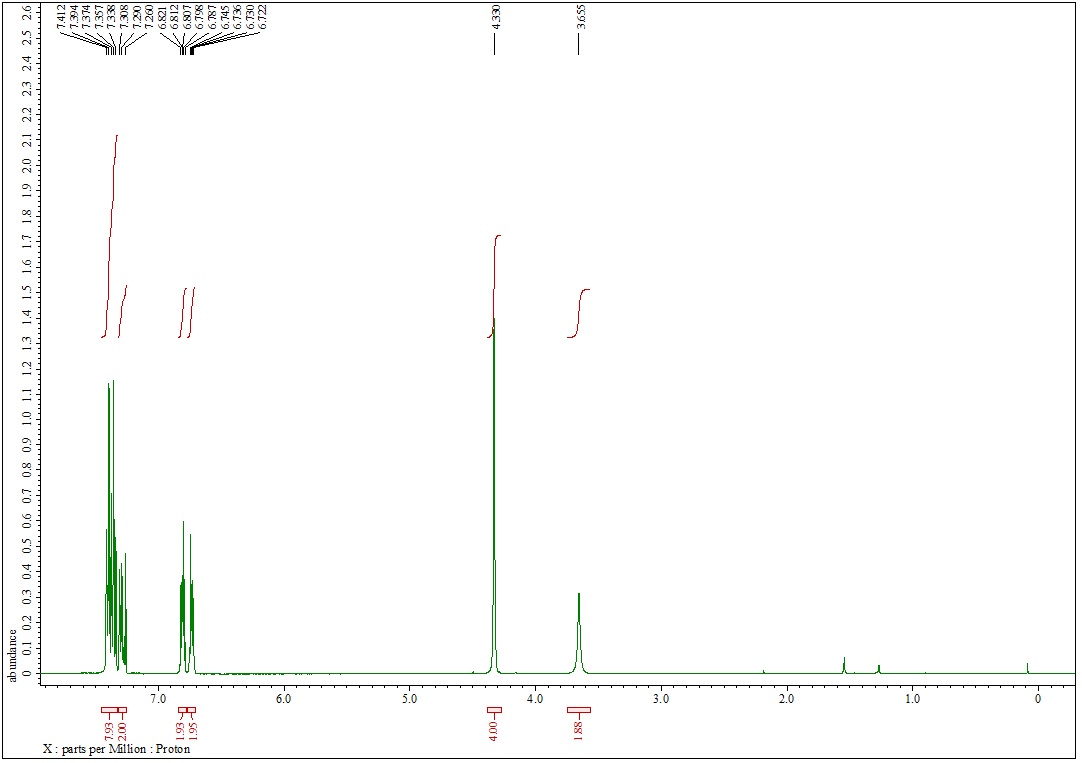


^13^C-NMR of compound**4** (100 MHz)


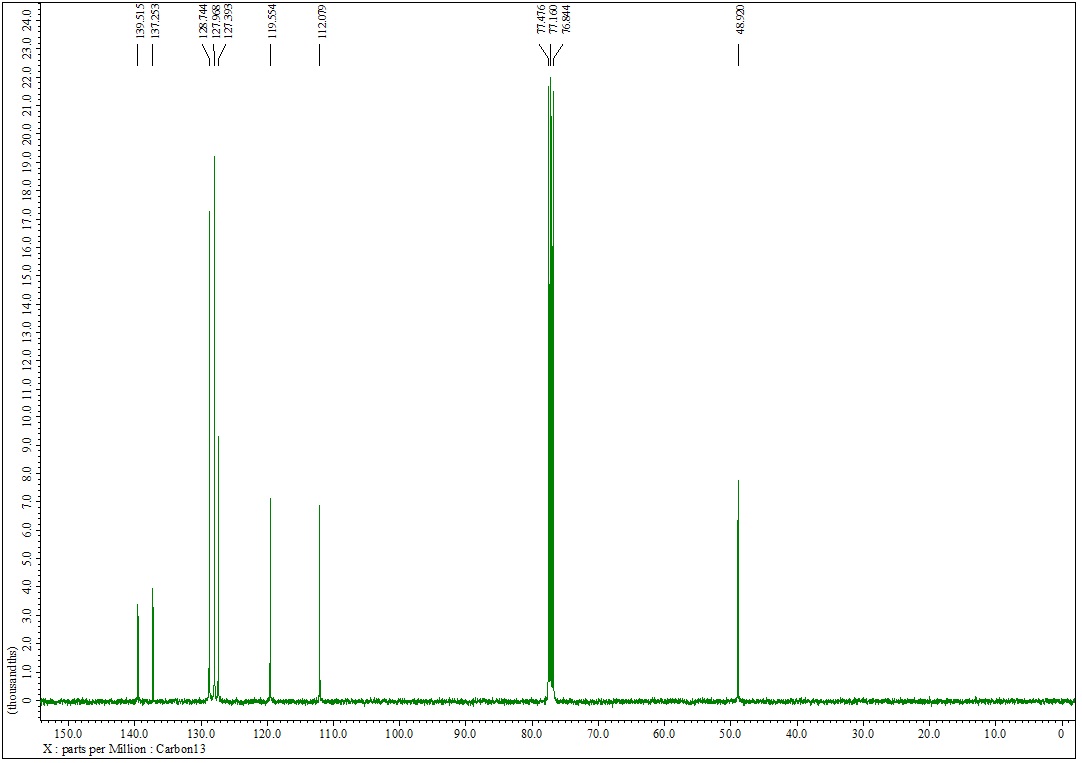


^1^H-NMR of compound **5a** (400 MHz)


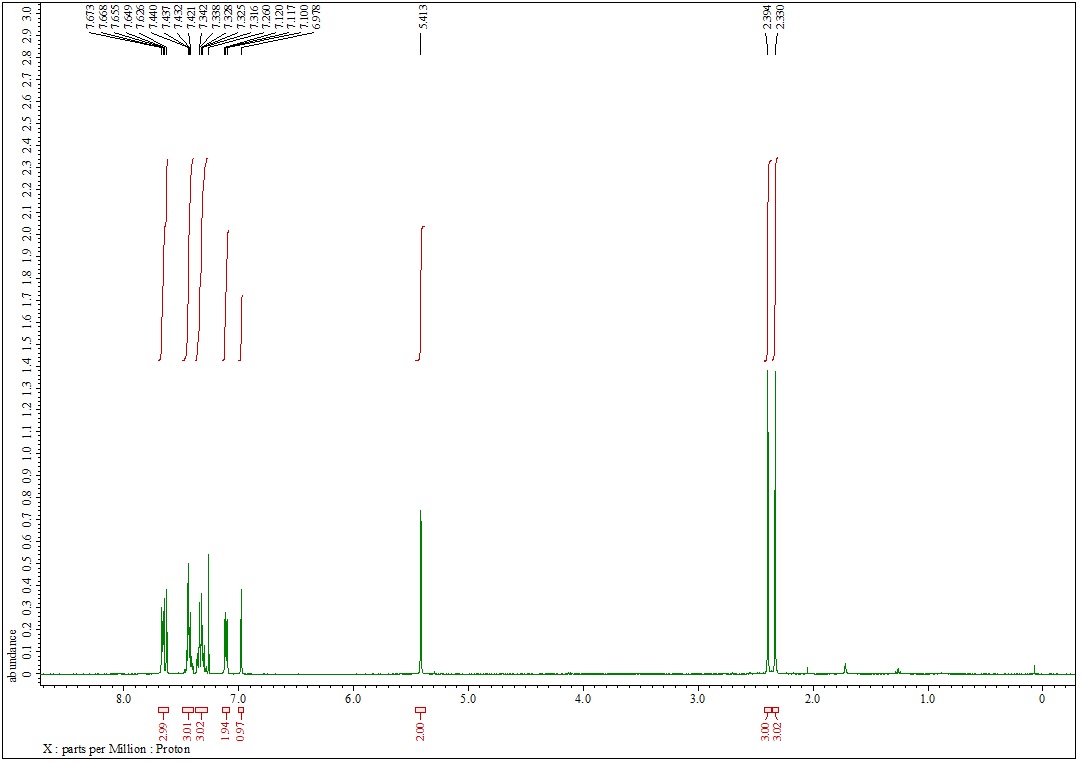


^13^C-NMR of compound**5a** (100 MHz)


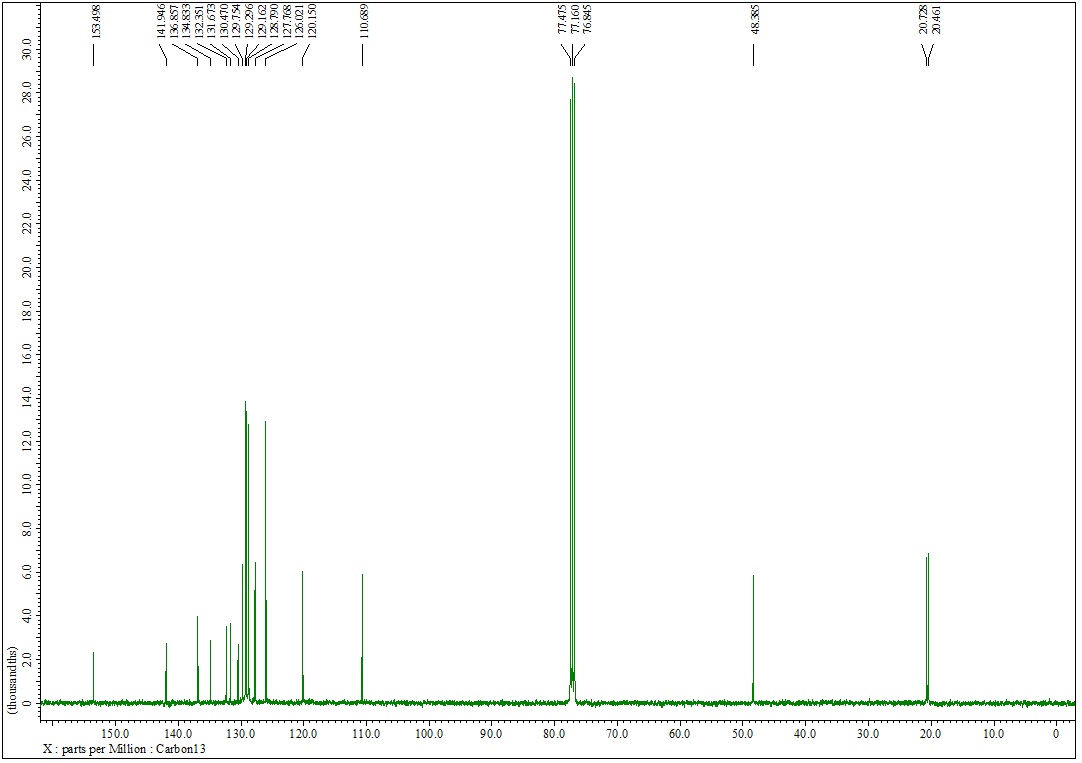


^1^H-NMR of compound **5b** (400 MHz)


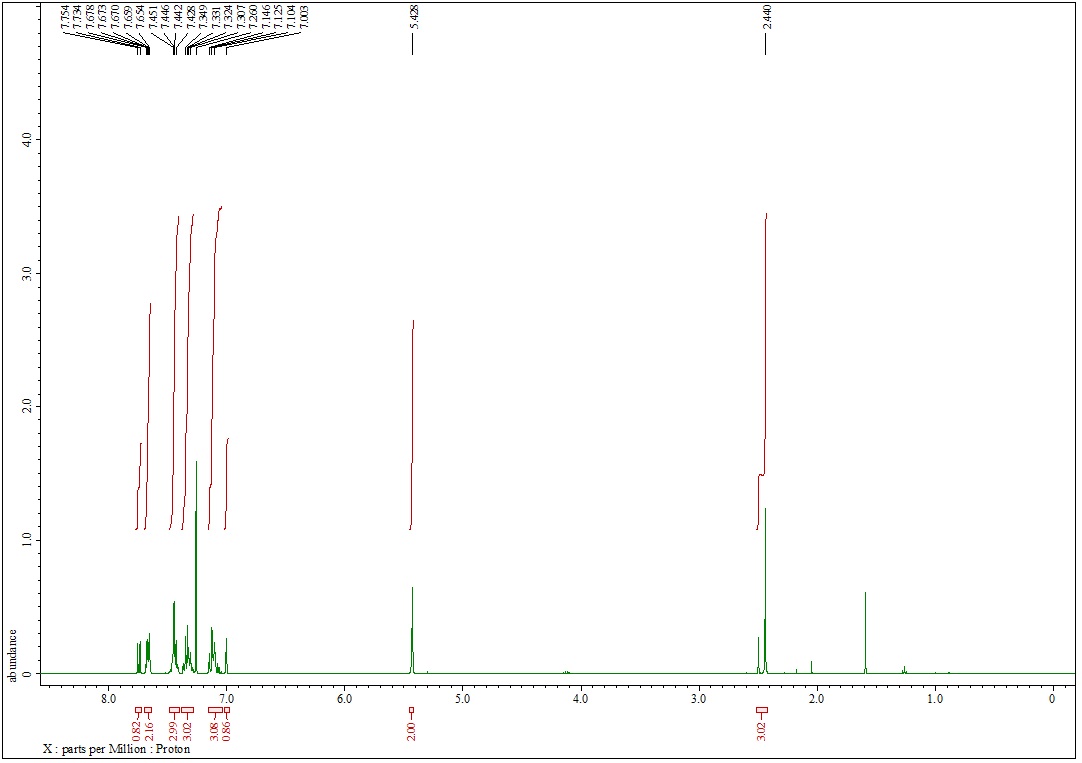


^13^C-NMR of compound**5b** (100 MHz)


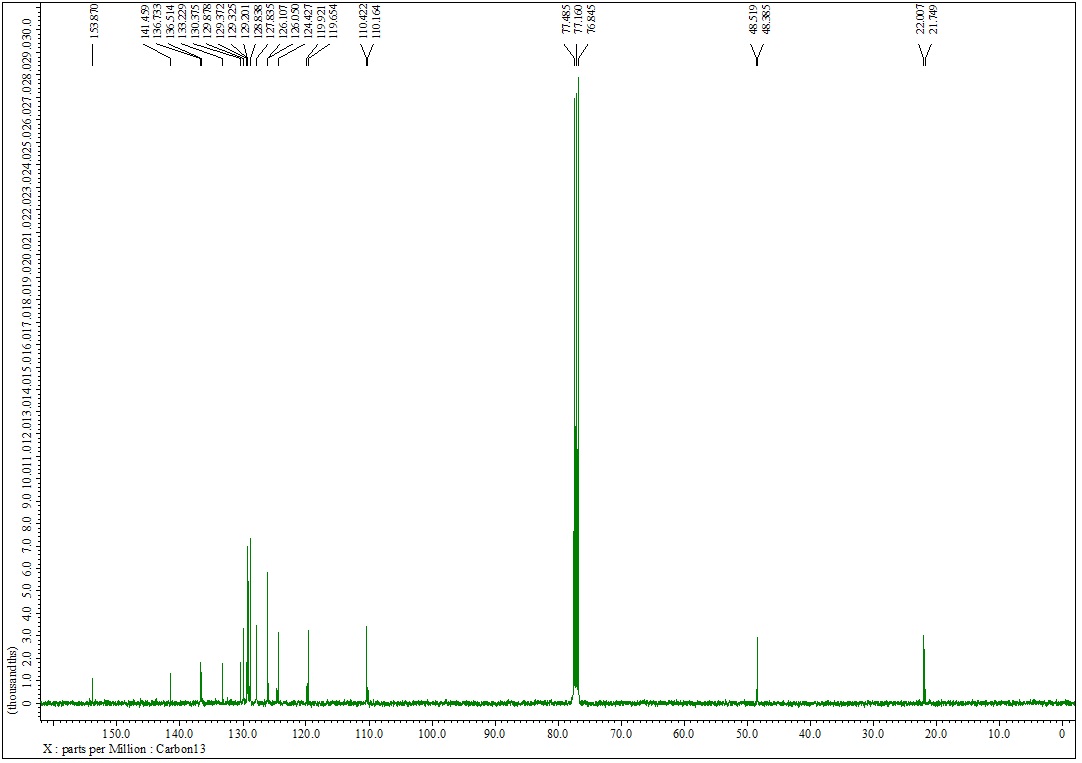


^1^H-NMR of compound **5c** (400 MHz)


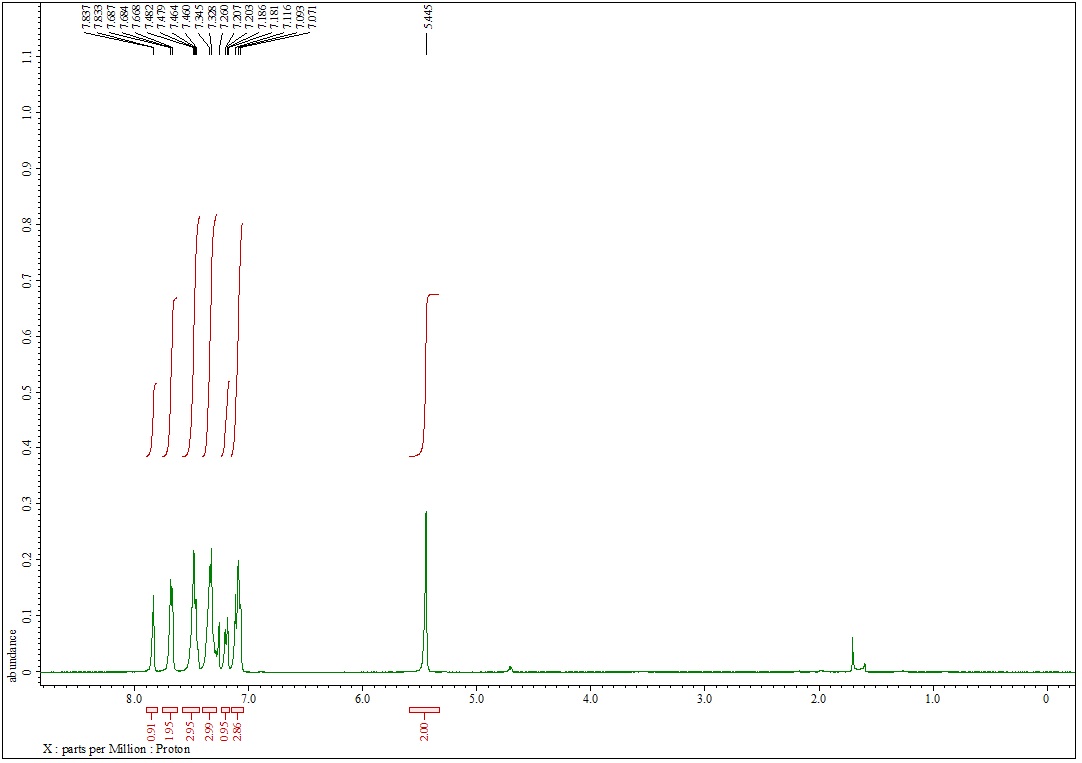


^13^C-NMR of compound**5c** (100 MHz)


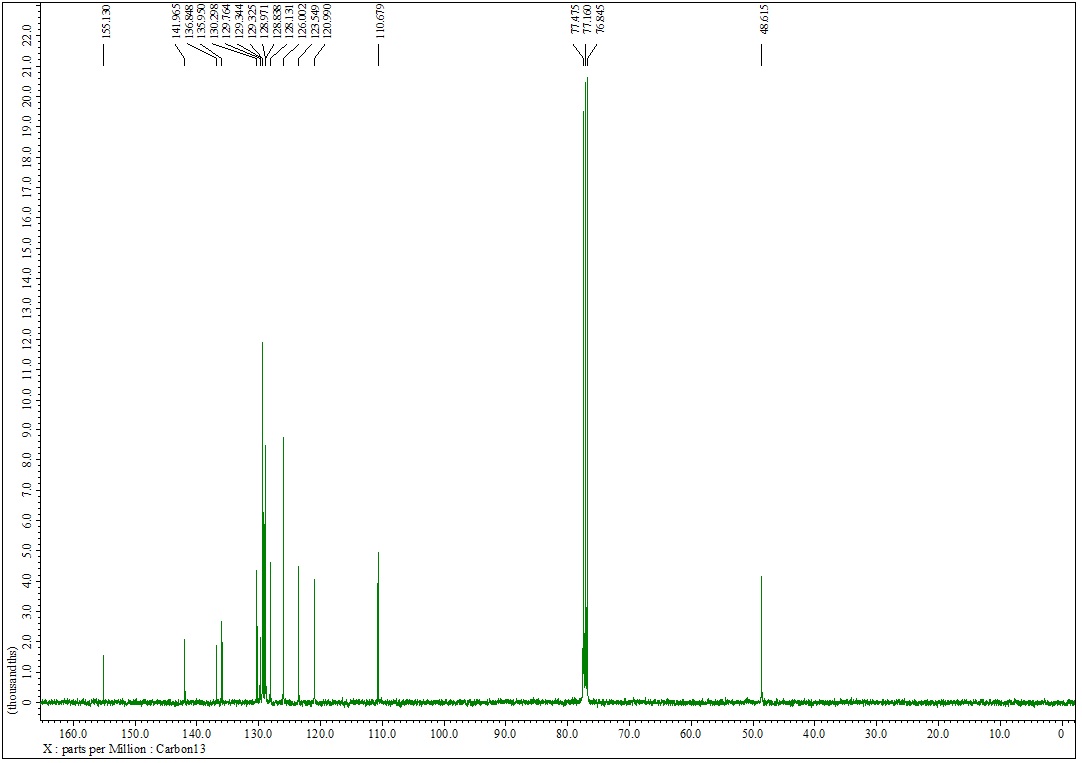


^1^H-NMR of compound **5c`** (400 MHz)


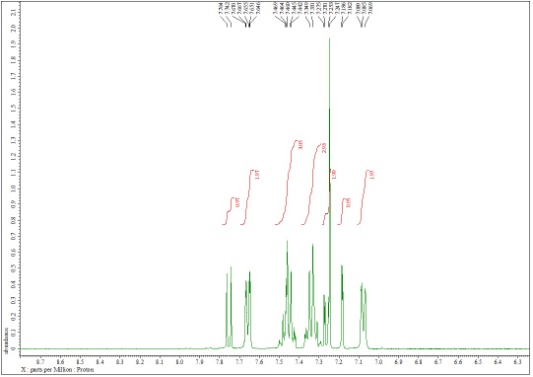

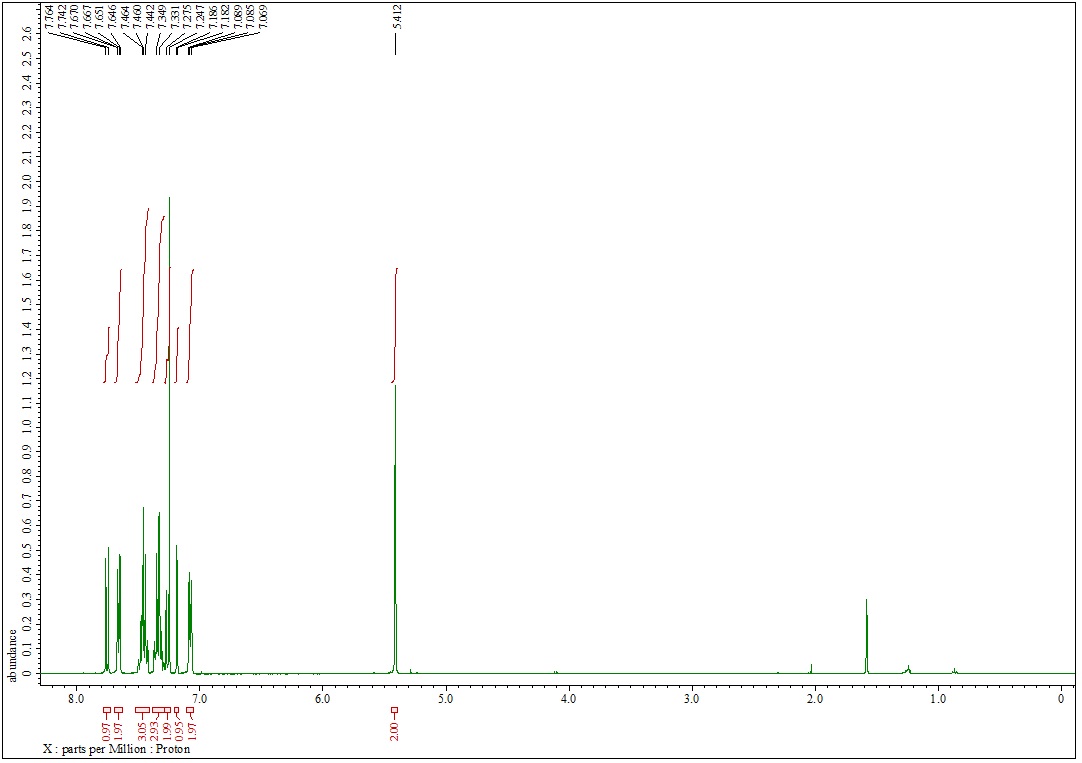


^13^C-NMR of compound**5c`** (100 MHz)


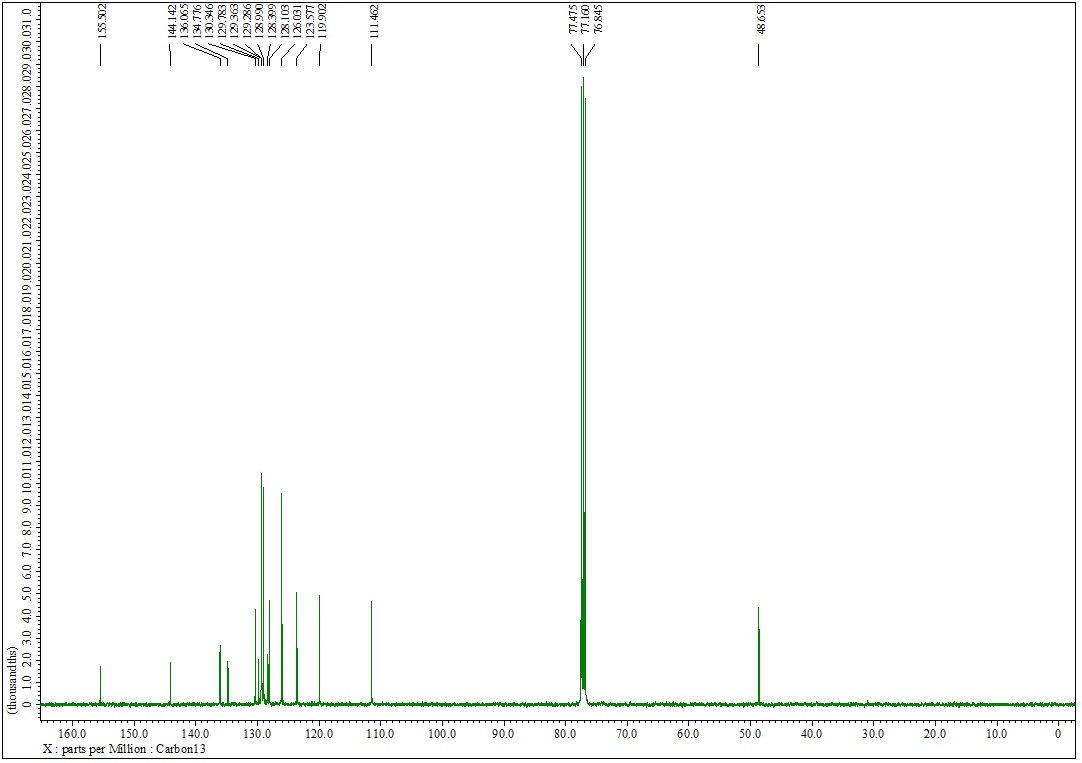


^1^H-NMR of compound **5d** (400 MHz)


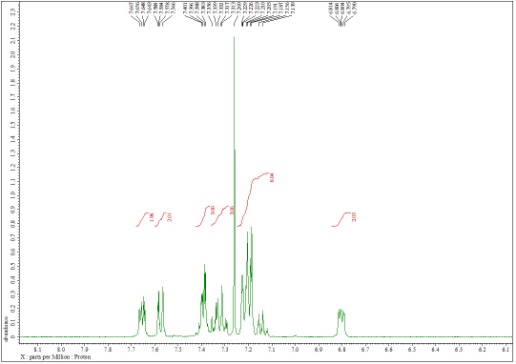

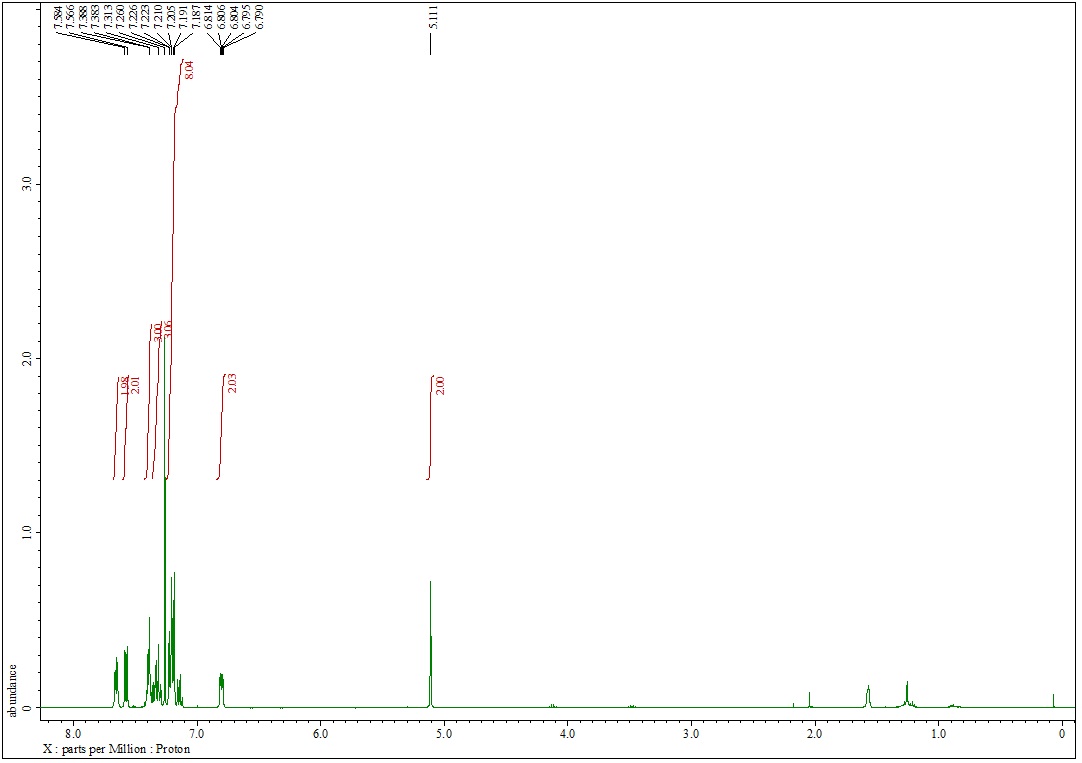


^13^C-NMR of compound**5d** (100 MHz)


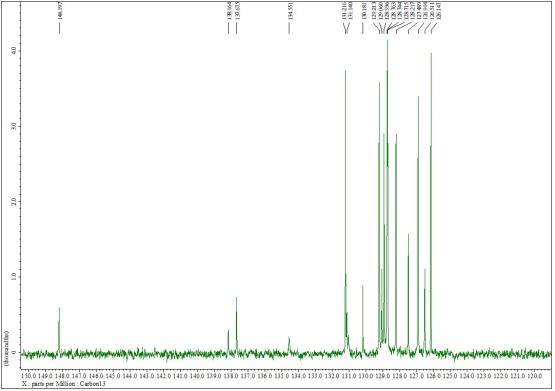

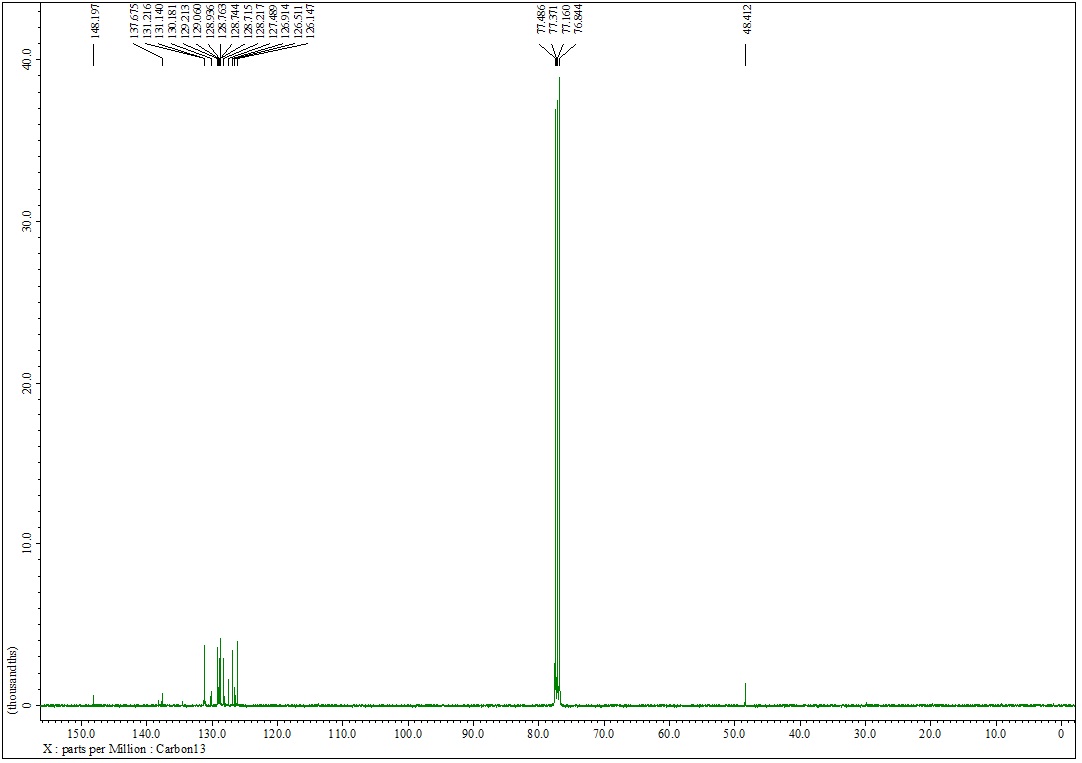


^1^H-NMR of compound **6** (400 MHz)

^
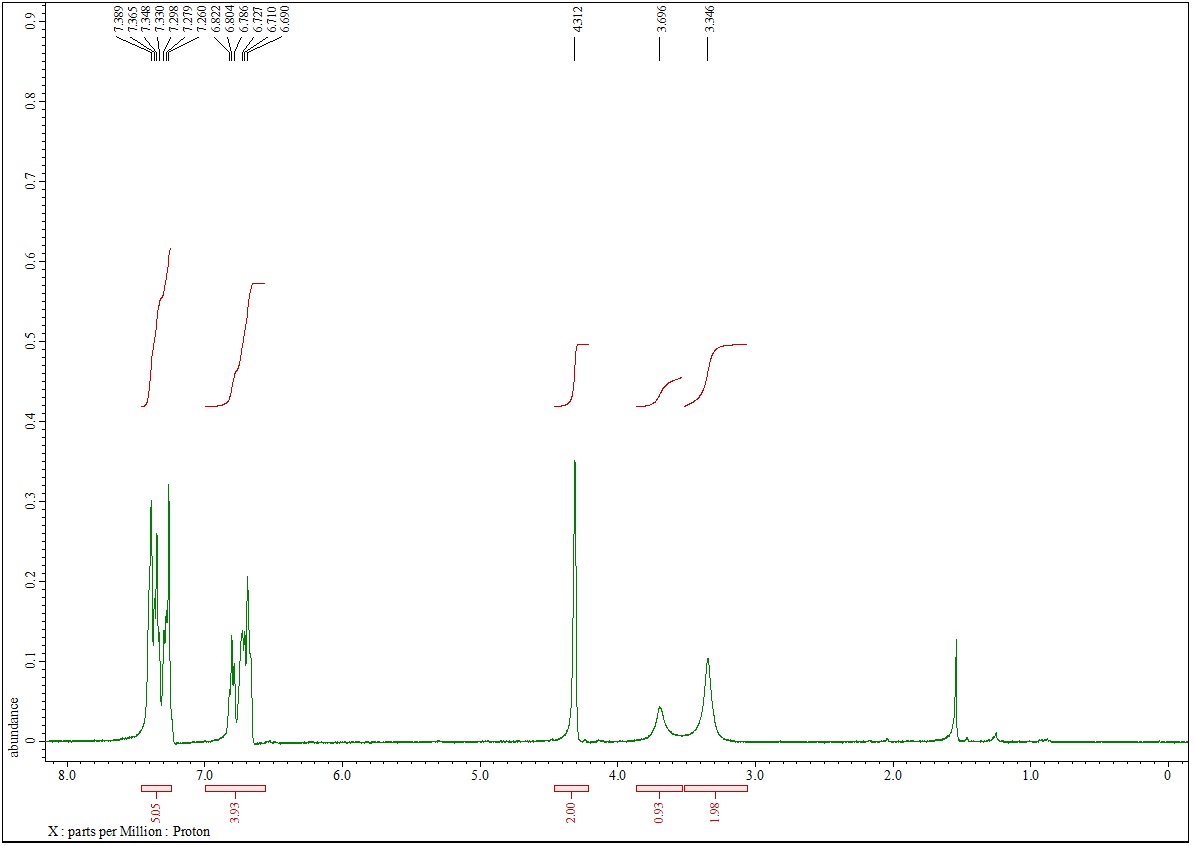
^

^1^H-NMR of compound **7a** (400 MHz)


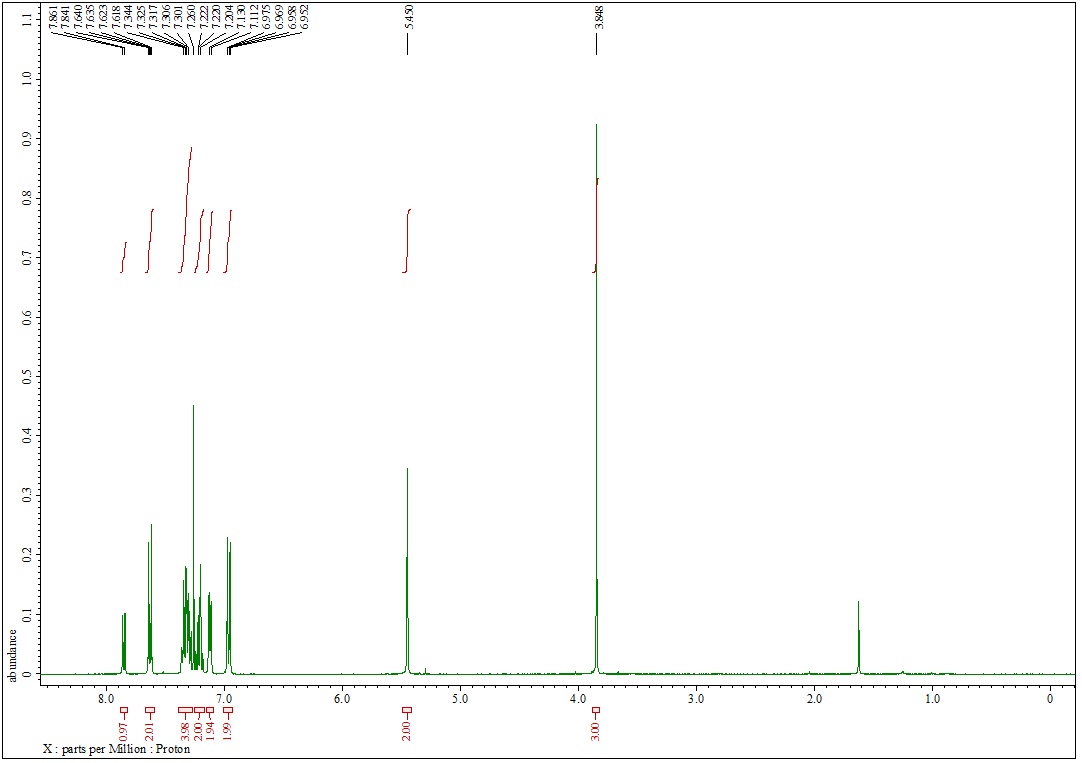


^13^C-NMR of compound **7a** (100 MHz)


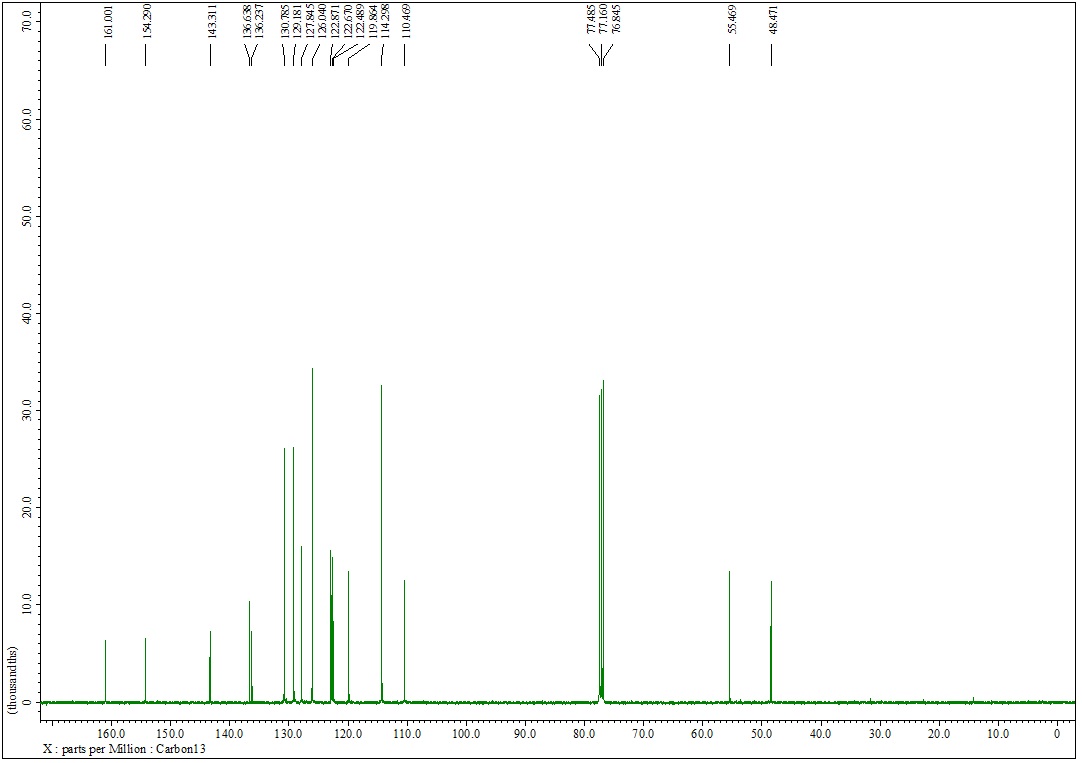


^1^H-NMR of compound **7b** (400 MHz)


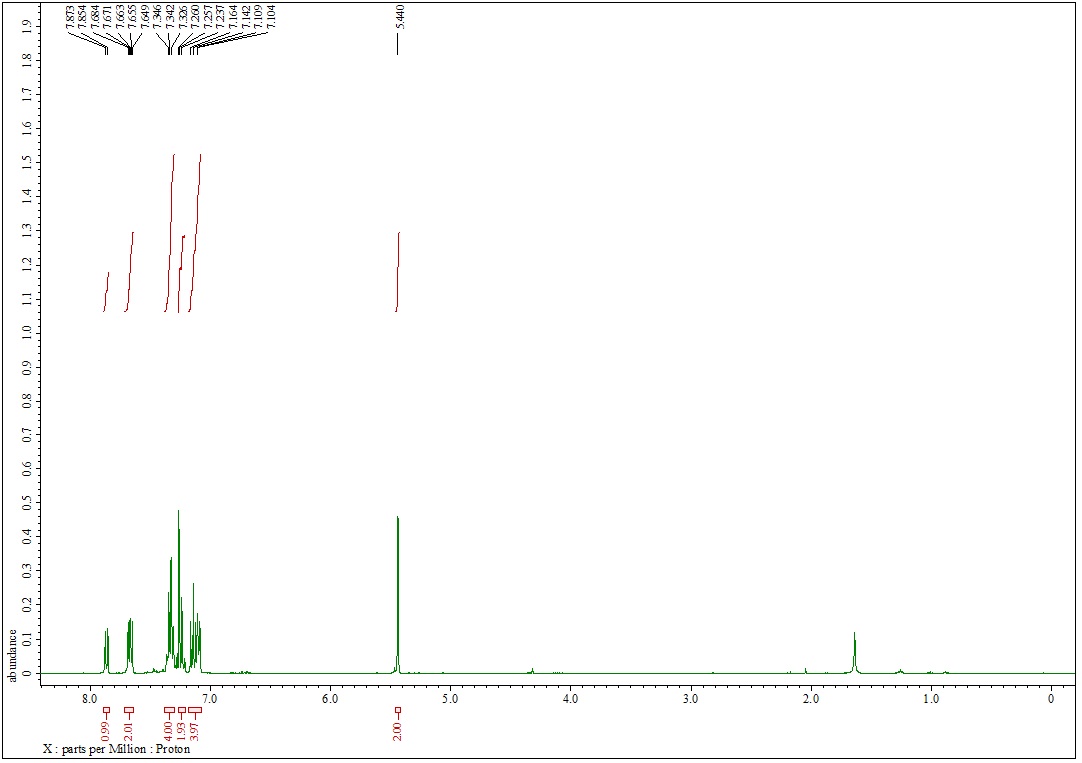


^13^C-NMR of compound **7b** (100 MHz)


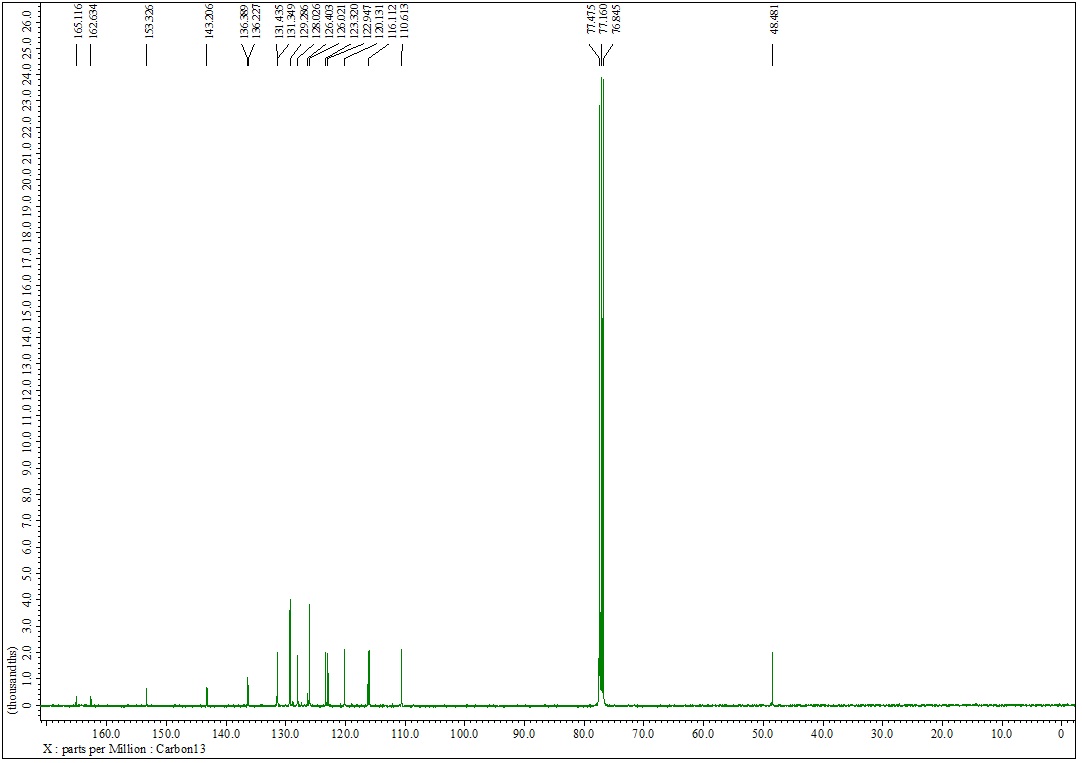


^1^H-NMR of compound **7c** (400 MHz)


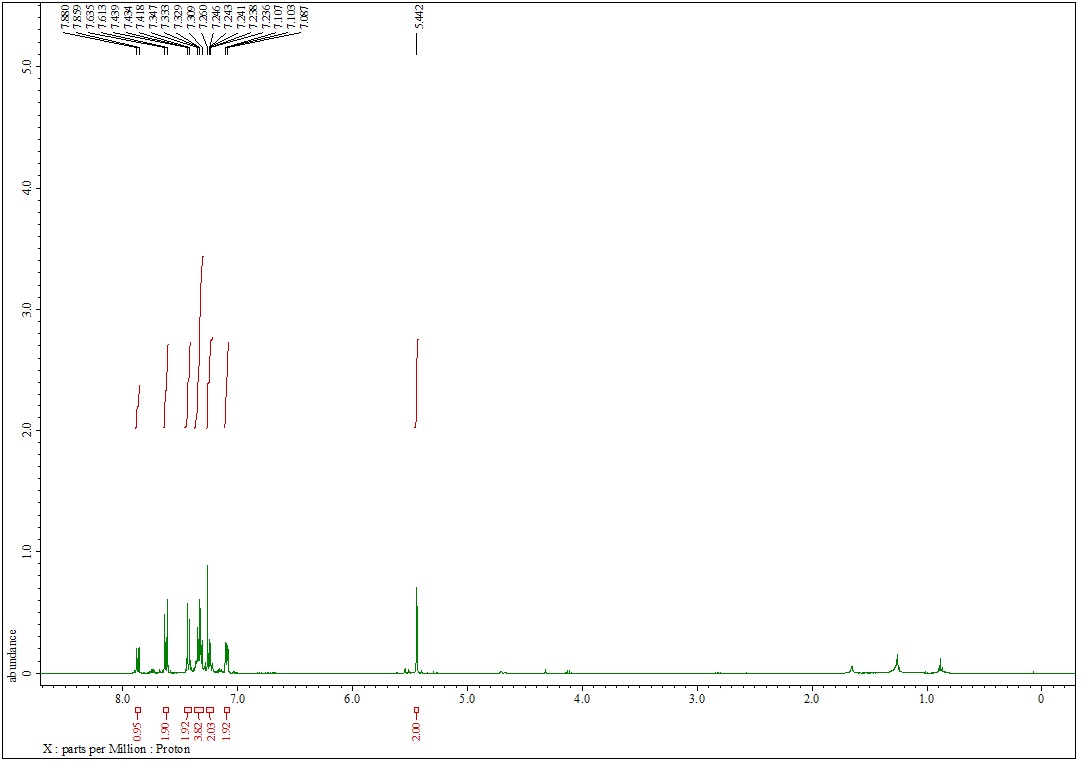


^13^C-NMR of compound **7c** (100 MHz)


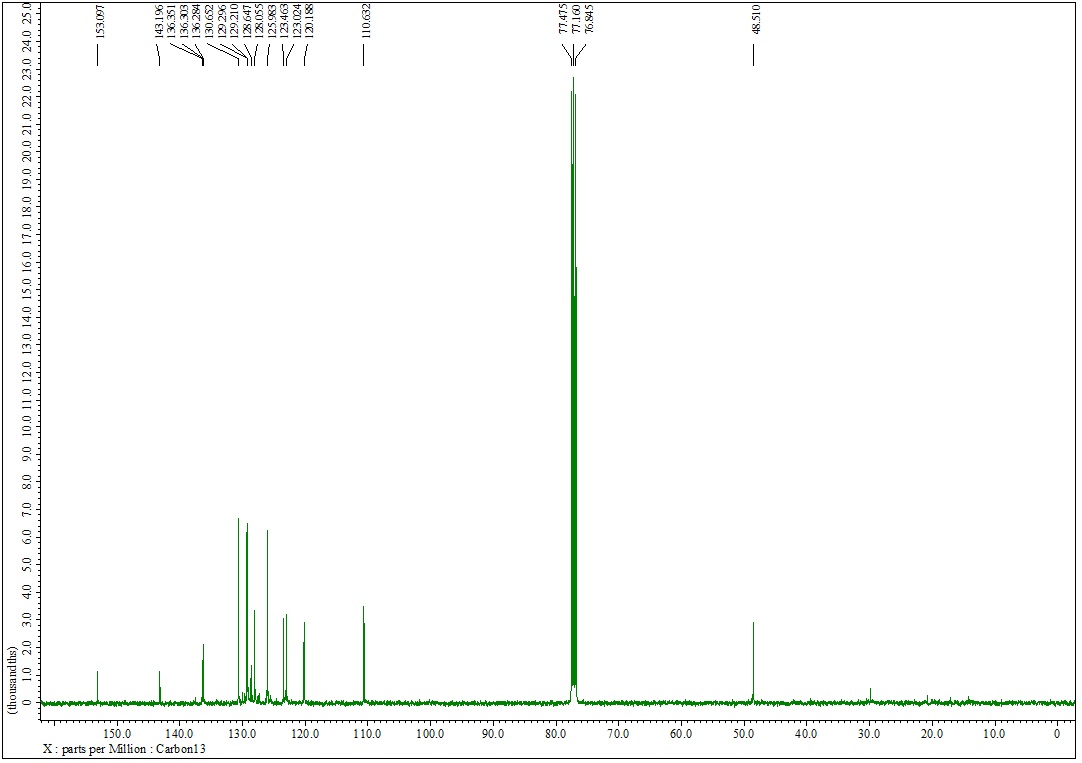


^1^H-NMR of compound **7d** (400 MHz)


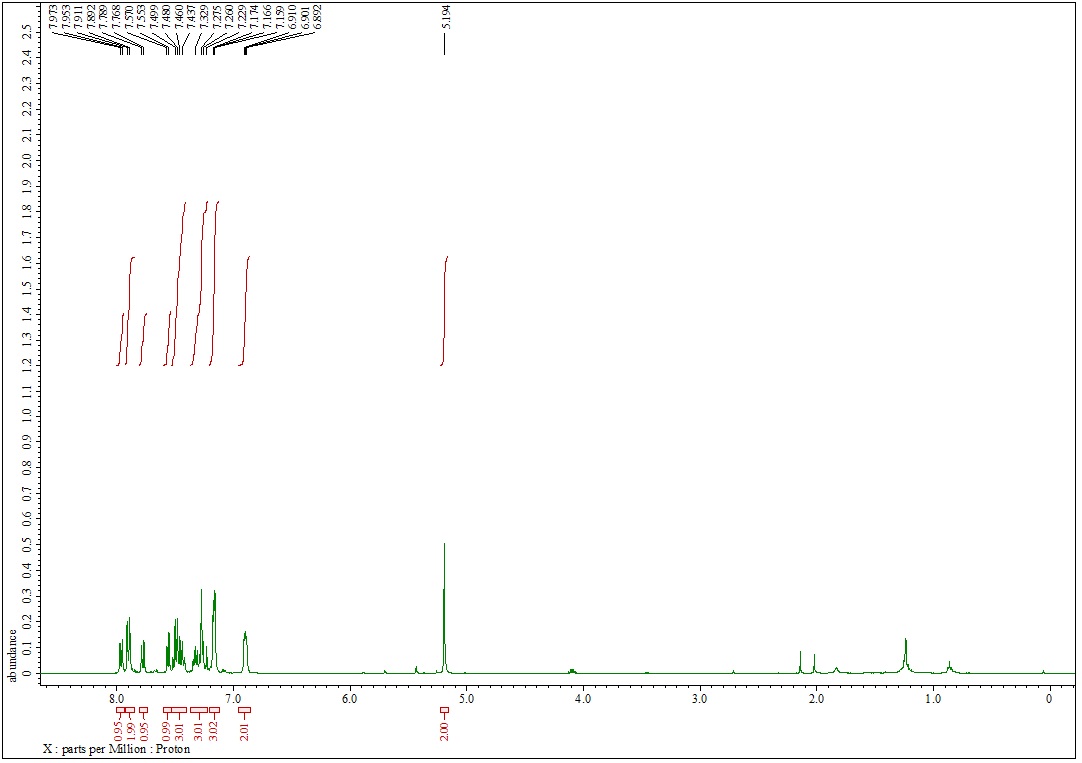


^13^C-NMR of compound**7d** (100 MHz)


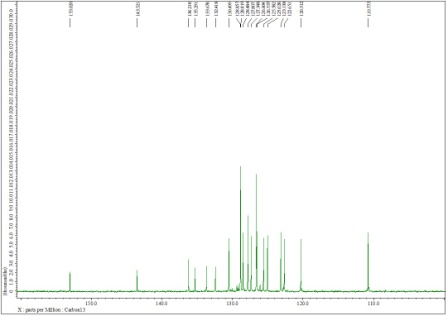

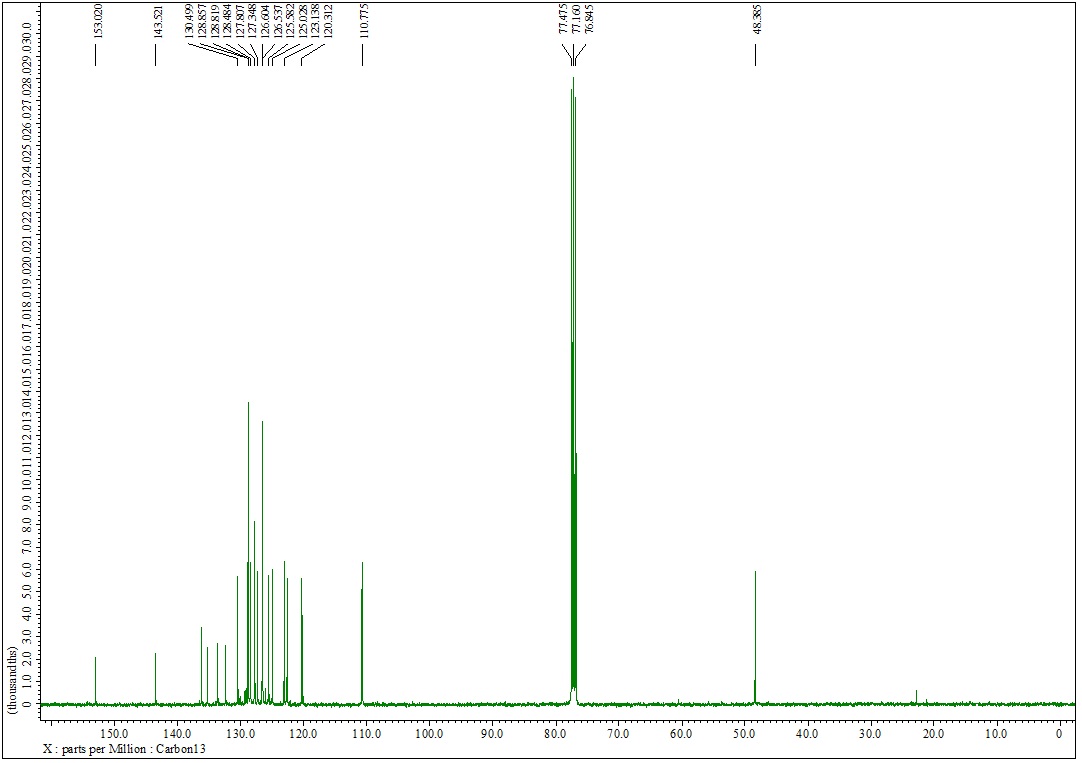


^1^H-NMR of compound **7e** (400 MHz)


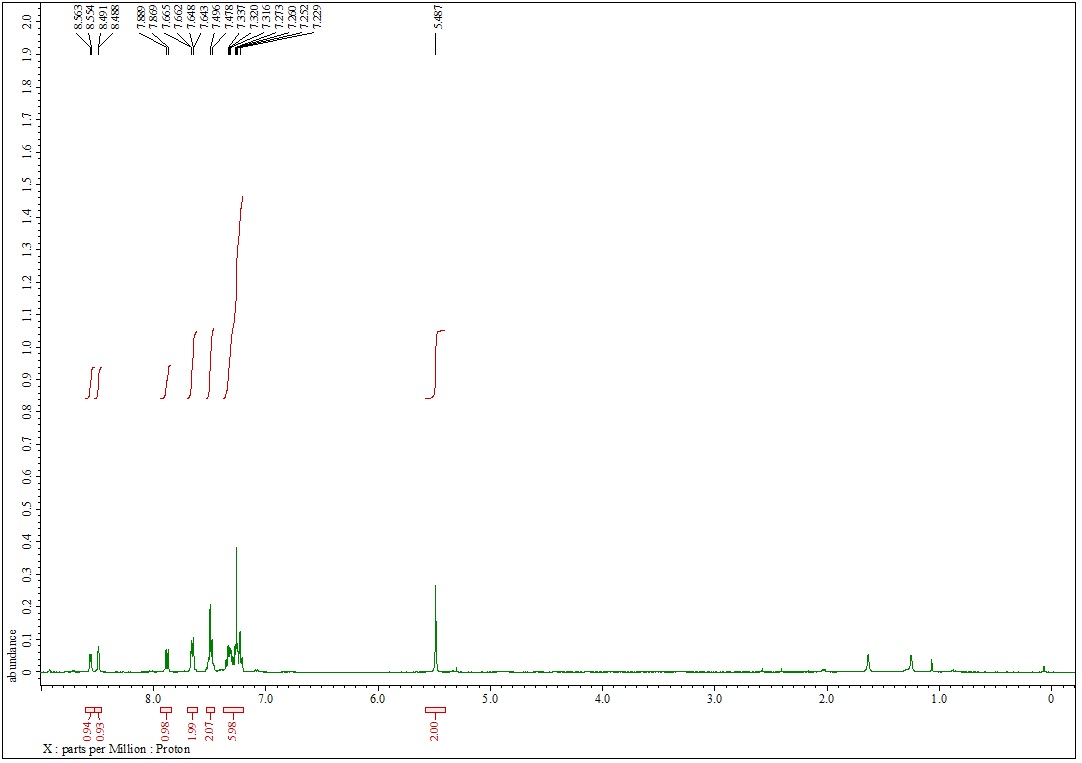


^13^C-NMR of compound **7e** (100 MHz)


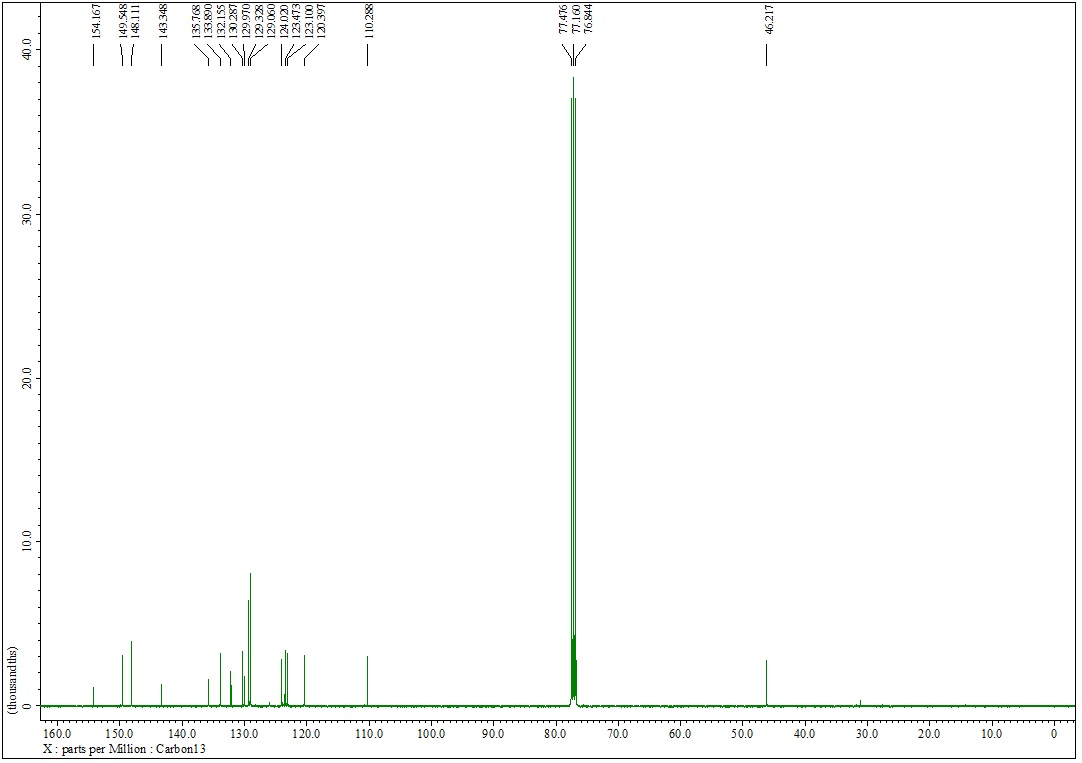


^1^H-NMR of compound **7f** (400 MHz)


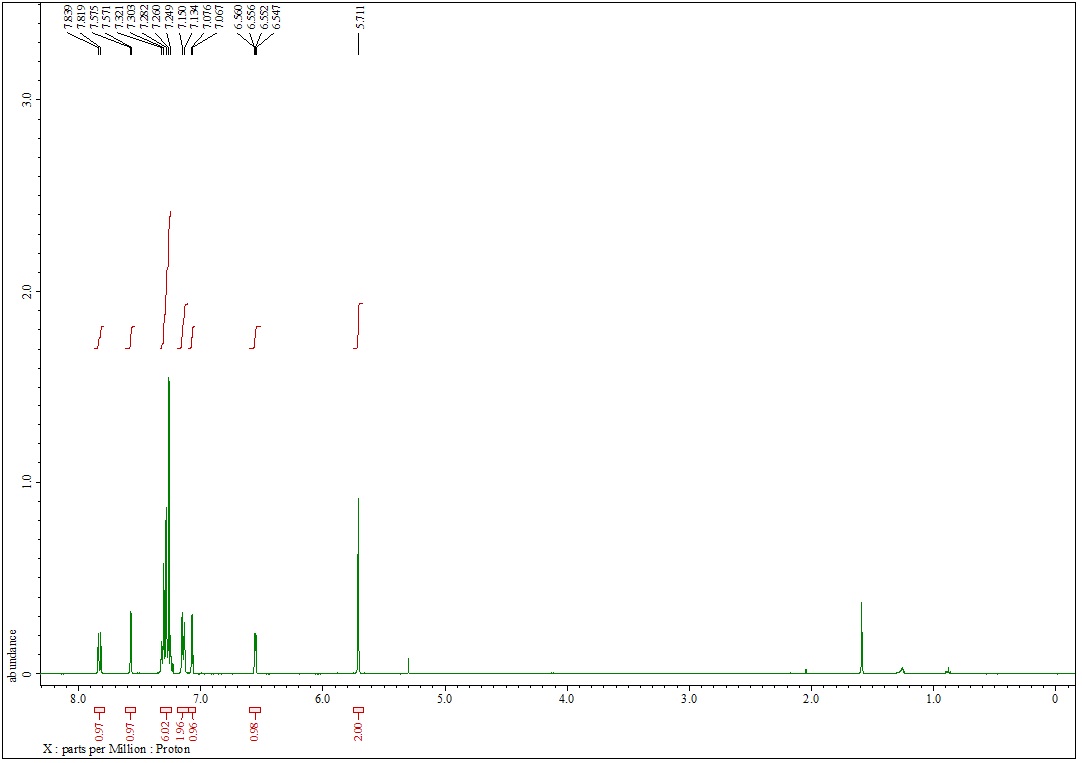


^13^C-NMR of compound **7f** (100 MHz)


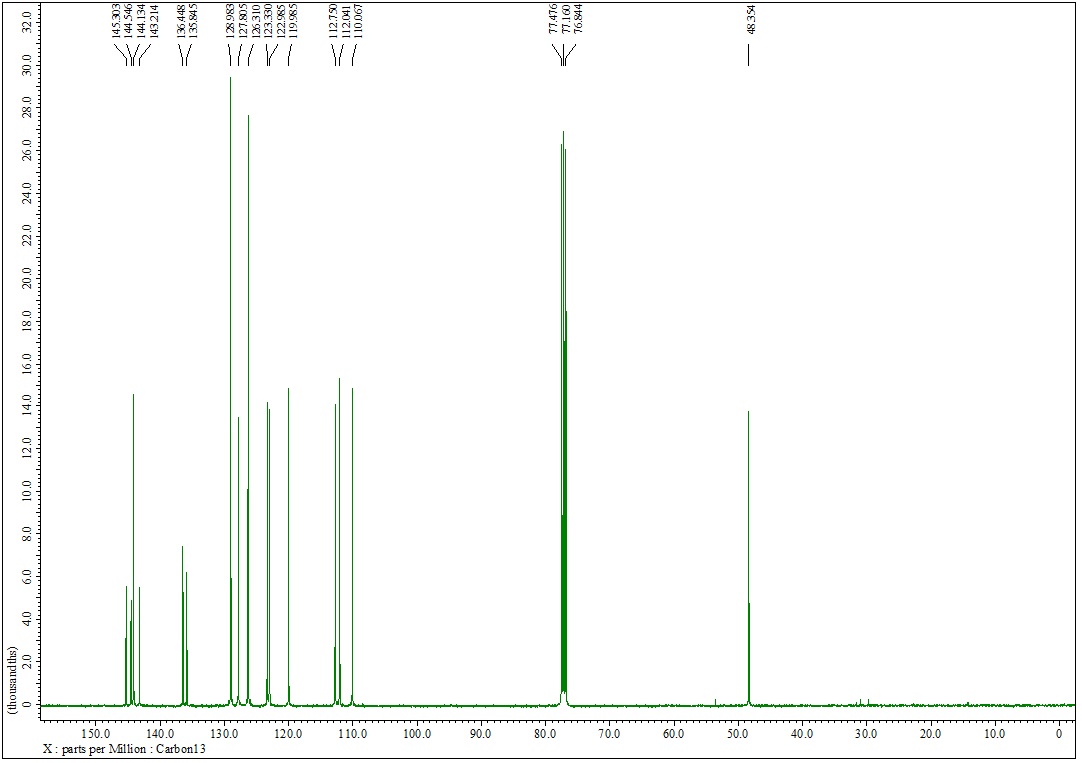


^1^H-NMR of compound **7g** (400 MHz)


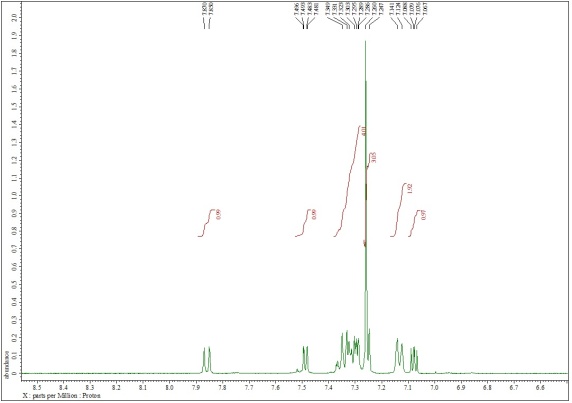

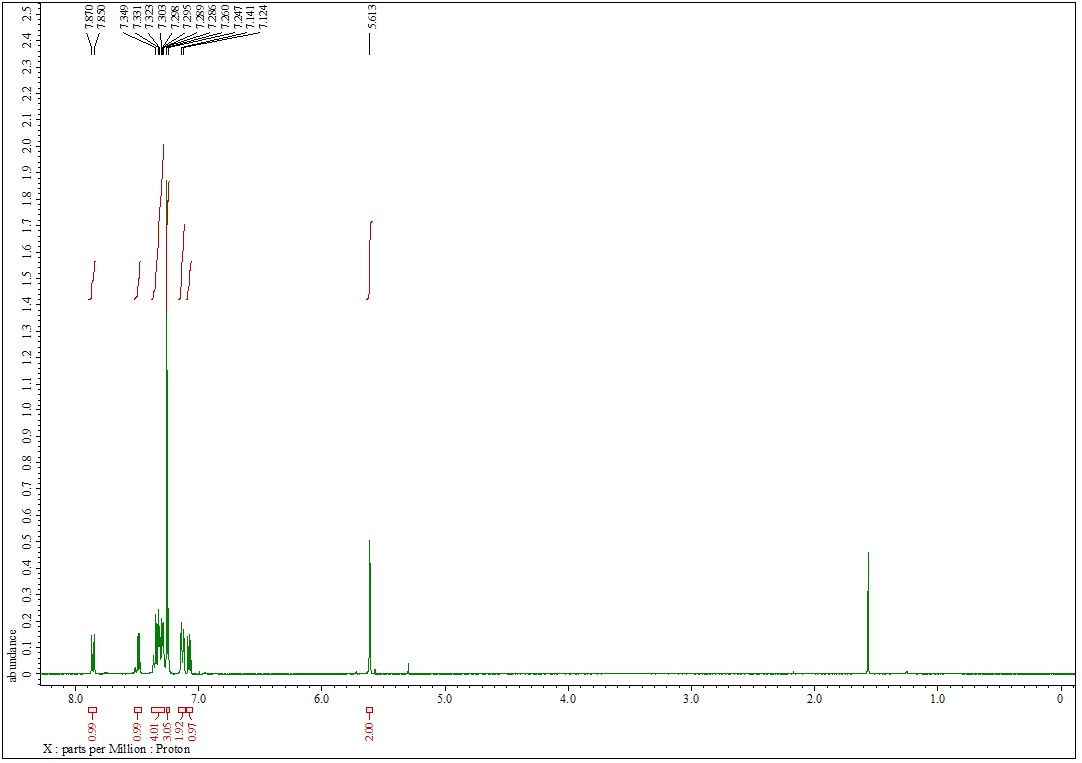


^13^C-NMR of compound **7g** (100 MHz)


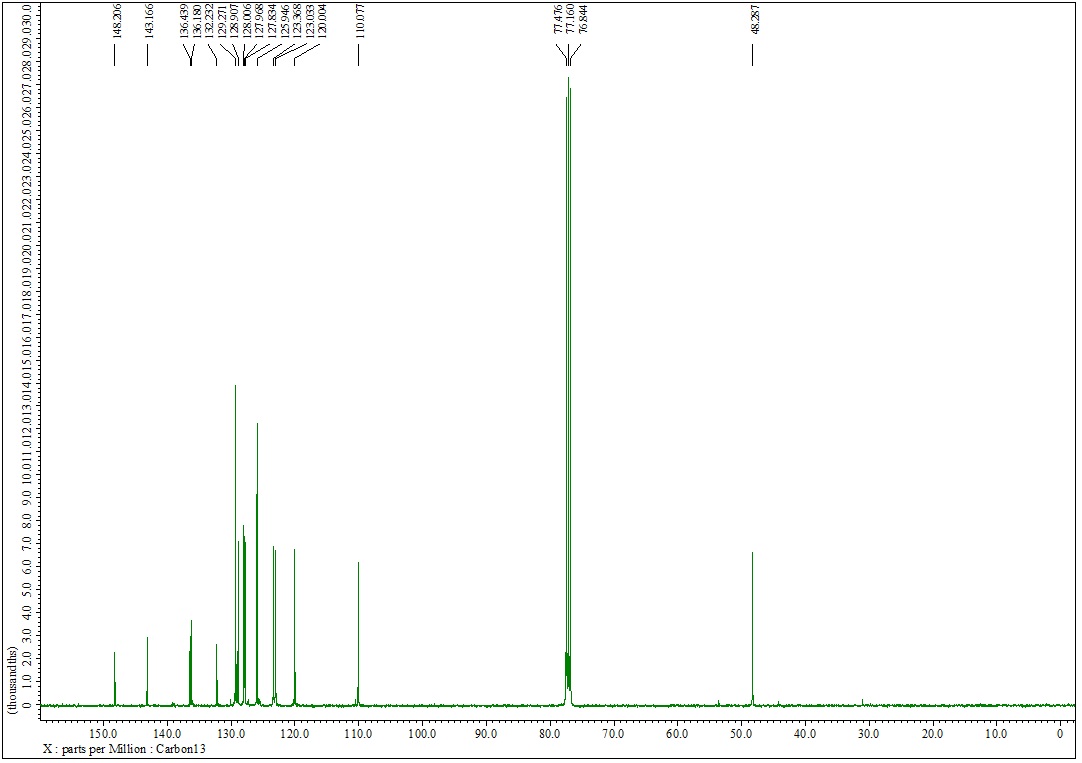


^1^H-NMR of compound **10** (400 MHz)


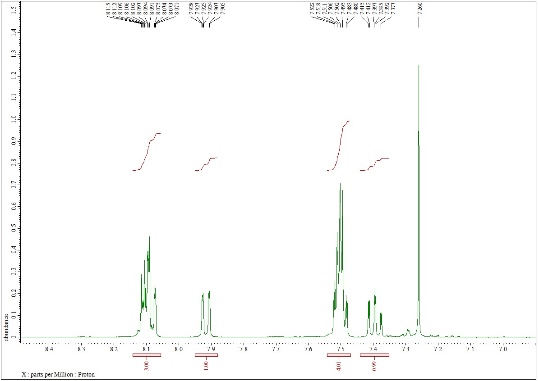

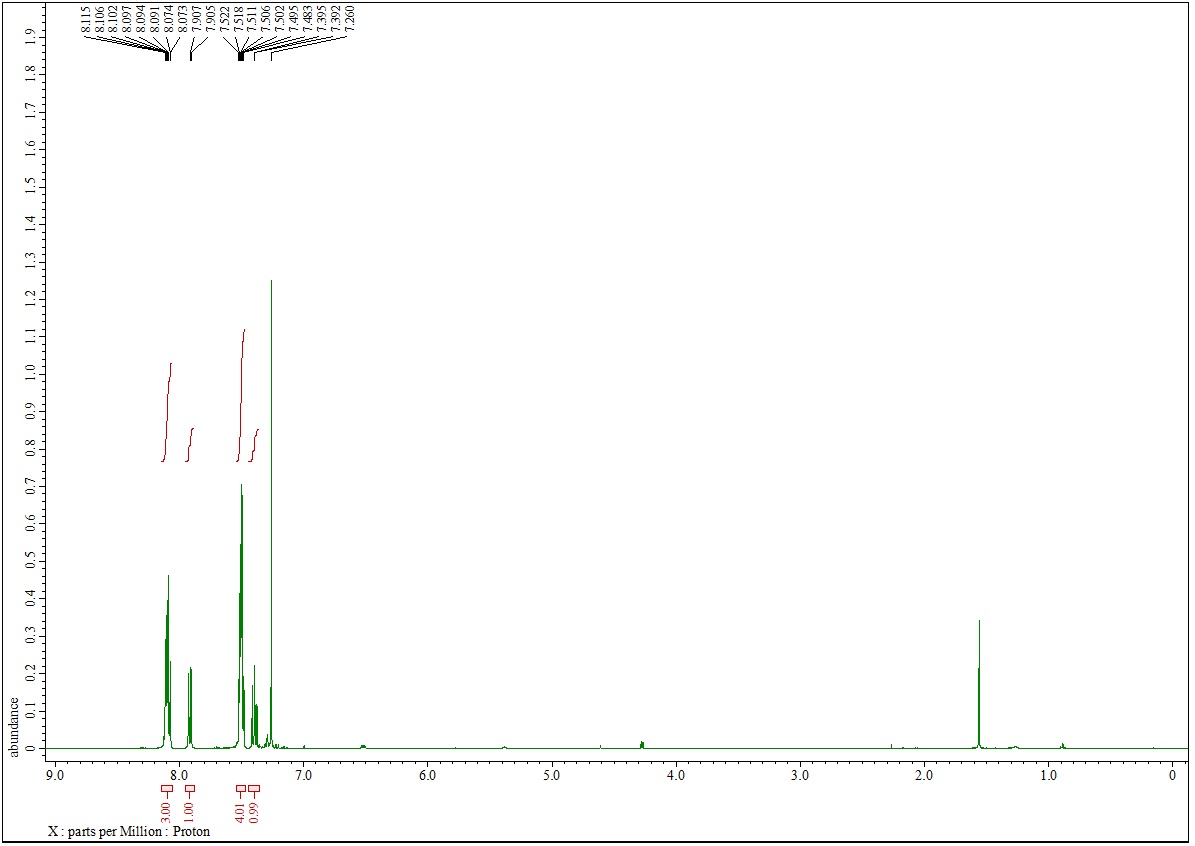


^13^C-NMR of compound **10** (100 MHz)


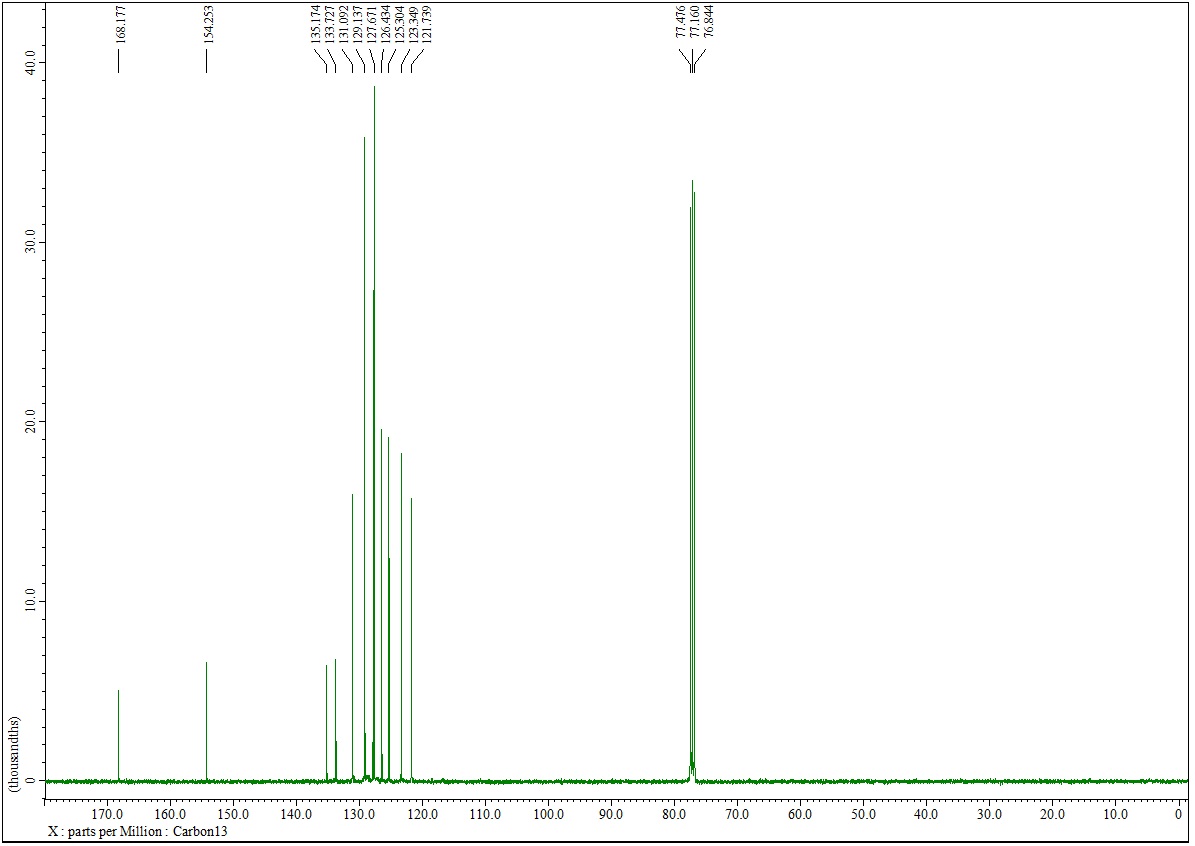

Supplement: Supplementary file 2 [file Table_1.DOCX]
